# Biomaterials’ Role in Improving Patient Care from Drug Testing and Delivery to Theragnostics and Regenerative Medicine

**DOI:** 10.3390/jfb17050214

**Published:** 2026-05-01

**Authors:** Sabina Cristiana Badulescu, Emma Adriana Ozon, Adina Magdalena Musuc, Manuela Diana Ene, Rica Boscencu

**Affiliations:** 1 Faculty of Pharmacy, “Carol Davila” University of Medicine and Pharmacy, 6 Traian Vuia St., 020956 Bucharest, Romania; sabina-cristiana.serbu@drd.umfcd.ro (S.C.B.); rica.boscencu@umfcd.ro (R.B.); 2 Biotehnos SA, 3-5 Gorunului St., 075100 Otopeni, Romania; 3 Institute of Physical Chemistry “Ilie Murgulescu”, Romanian Academy, 060021 Bucharest, Romania; amusuc@icf.ro; 4 Faculty of Medical Engineering, “Politehnica” National University of Science and Technology, Gheorghe Polizu 1-7 Street, 011061 Bucharest, Romania

**Keywords:** smart biomaterials, intelligent biomaterials, polymer database, theragnostics, nanomedicine, drug-delivery system, drug release, bioprinting, tissue engineering, regenerative medicine

## Abstract

Over the past 200 years (1820–2020), global life expectancy has nearly tripled, increasing from 26 to 72.91 years, due to factors such as poverty reduction and public health initiatives. Today, society faces different challenges than it did centuries ago. In patient care and healthcare system priorities, the goal is to develop smart, feasible, long-lasting, cost-effective, readily available, adverse-reaction-free, adaptable, and personalized solutions that minimize patient discomfort, reduce caregiver effort, and decrease hospitalization duration and costs. In this context, biomaterials serve as versatile tools capable of performing a wide range of diagnostic, therapeutic, and theragnostic functions. Thanks to their biocompatibility, biodegradability, surface chemistry, and responsiveness, biomaterials are currently addressing issues such as patient compliance (through controlled drug-delivery systems and smart wound dressings), long transplant waiting lists, transplant rejection, non-adaptable prosthetics (artificial organs), oncology treatment efficacy (nano-formulations for theragnostics and multiple tumor targeting), and inconsistent in vitro drug-testing models (organs-on-a-chip). In this review, we focus on biomaterials’ smartness, then explore databases for efficient product design, and finally highlight their applications in the biomedical field, especially in drug delivery, tissue engineering, and regenerative medicine.

## 1. Introduction

Over the past two centuries, humanity has optimized several life parameters in its favor: living standards, education, and economy, as well as public health and patient care (including clean water, nutrition, sanitation, antibiotics, vaccines, medicines for chronic and rare diseases, technologies for new drug design, and methods for improving patient conditions, such as neonatal, geriatric, or personalized healthcare). This progress has led to an almost threefold increase in life expectancy [1]. A longer life means the human body must be supported for endurance rather than a sprint. In today’s more sedentary, mentally stressed, and aging society (by 2030, one in six people worldwide is expected to be over 60, and by 2050, this age group is projected to reach 22% [2]), with approximately 17% of the global population being smokers [3,4] and 16% obese [5], thus at risk of developing cardiovascular, pulmonary, and/or musculoskeletal conditions. One in three people lives with multiple chronic conditions [6,7], about one in five develops cancer in their lifetime [8], and 2% experience chronic wounds, including difficult-to-heal injuries [6,9]. There is a pressing need for smart, long-term, cost-efficient, feasible, and effective medical solutions that maximize patient benefit regardless of age (from newborn to elderly), support caregivers—preventing them from becoming patients due to the strain of caregiving—and enable the medical system to provide simple, durable, and adaptable yet personalized solutions. To meet this need, several directions can and have already been addressed to some extent, including: designing smart products for rapid and non-invasive diagnostics and self-monitoring (biosensors); creating medicines and medical devices that require less frequent administration or replacement and can maintain a constant plasma or local drug concentration with minimal adverse reactions (improving patient compliance); combining treatment and diagnostics within the same method or product (reducing costs); offering grafts, prosthetics, and artificial organs that integrate seamlessly and adapt with the recipient’s body, eliminating the need for transplants or the risk of rejection; and building more accurate in vitro drug-testing platforms to minimize the preclinical trial phase of new drugs. All these solutions share several key features: they are intended not to disturb the body’s homeostasis, to mimic human tissue characteristics and aid regeneration, and to be as biocompatible and targeted in their action as possible. To achieve these goals, the materials used in these medical products must closely resemble our own cellular and tissue microenvironment. The only class of materials that possesses these characteristics, apart from our own body’s molecules and cells, is biomaterials.

## 2. Methodology

This study’s design involved three main steps:

1. Advanced search in the Web of Science Core Collection database
  The main objective of this step was to establish a core framework for the study’s direction. A secondary objective was to identify research trends in biomaterials (related to tissue engineering, regenerative medicine, personalized medicine, drug testing and delivery, and theragnostics), as well as potential research gaps.
2. Search in other databases and web pages for in-depth, explanatory, and complementary information
  The purpose of this step was to supplement the framework from step 1 with relevant information and to address the identified research gaps.
3. Integration and new output
  The focus of this step was to generate original content that researchers can use as a tool, featuring an easy-to-use interface.

1. Advanced search in the Web of Science Core Collection database

The advanced search of the literature was performed in the first step using a unique database: Web of Science Core Collection (WoS), which was accessed through the E-nformation platform—login via an institutional profile (https://www.e-nformation.ro/profil-acces, accessed on 10 January 2026) in order to have access to the Advanced Search menu option from WoS. In the Advanced Search tab, the Query Builder was used for all the searches, because it offers complete control over the syntax that is built (keywords, operators, filters), and it saves any generated result as a new “set” (a set of bibliographic results) at the bottom of the page. This function allows us to easily compare the results (bibliographic sets) that we obtain based on the query and further refine our search.

We performed our study in “All Editions” of the Web of Science Core Collection database, and adopted a search based on topic (TS), which means that the query is applied in the following metadata: title, abstract, and document keywords, which we found relevant enough. We used the star symbol (*) after individual keywords or syntagms used in the query, because this way we ensured that all similar terms, spellings, and forms of the word, differing by any number of characters from ours, were included (e.g., biomaterial* = biomaterial, biomaterials, bio-material, bio-materials, etc.).

We used different queries until we were satisfied with the following one: TS = (biomaterial* and (tissue engineering OR TE or regenerative medicine* OR RM OR TERM OR personalized medicine* OR drug test* OR drug delivery* OR theragnostics* OR bioprint* OR 3D print* OR 4D print*)), which initially generated 52,580 results. Through this syntax, we wanted to identify all bibliographic resources that contained in the previously mentioned metadata the keyword biomaterial(s) AND at least one of the other keywords separated through the “OR” operator (theragnostics, drug delivery, regenerative medicine, bioprint, etc.) because we were interested in the relationship between biomaterials and these terms and how the niches created by the combination of these fields interpenetrate. We also added the abbreviations TE (tissue engineering), RM (regenerative medicine), and TERM (tissue engineering regenerative medicine) to the search syntax because they are sometimes found in the abstract, even if the title or keywords selected by the authors do not include these terms in extenso.

After applying a publication date range filter for a custom period (1 January 2021 to 31 December 2025, the last five complete years), the obtained database contained 21,611 results. We refined this by including only review articles (filtered by document type), which resulted in 7251 results. Further inclusion criteria were applied: highly cited papers (326 results)/hot papers (13 results)/highly cited and hot papers (11 results). It is important to know that:

- Highly cited papers represent those articles that rank in the top 1% of citations for their specific field and publication year. Citations are compared within 22 specific research fields and against papers published in the same year, adjusting for differences in citation habits. Only papers published in the last 10 years are considered.
- Hot papers represent those articles that receive a large number of citations soon after publication, relative to other papers of the same field and age. They are papers published in the past two years that received a number of citations in the most recent two-month period, placing them in the top 0.1% of papers in the same field.

**Figure 1:**
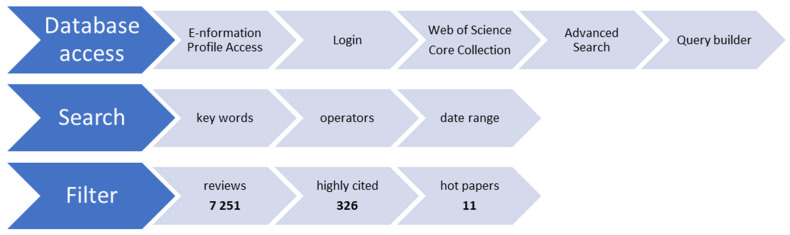
Advanced search process diagram.

The last inclusion criteria or filter applied (highly cited and hot papers) generated our core-frame bibliographic selection (Supplementary Figure S1). The step-by-step process of this advanced search can be followed in the diagram presented in Figure 1.

All the databases were downloaded both as Excel and plain text files, using the Full Record option in terms of metadata and the “Records from 1 to … (last item)” option to download every entry; for Db7251, eight files were downloaded for each extension, since one file can contain a maximum of 1000 items. The Excel files were used to identify the publication names and authors, but no further refinement was carried out with them. The plain text files were used for analysis in the VOSviewer software.

Although the core database (11 highly cited and hot review papers published between 2021 and 2025—Table S1, Supplementary Materials) offered us a picture of the present-day main research and funding directions, the last three databases obtained after each inclusion criterion (filter) were analyzed in order to obtain a clearer view of the existing studies over collateral research trends, and especially over potential research gaps.

First of all, a general preview was obtained using the “Analyze results” and “Citation report” functions in the Web of Science platform for each of the above-mentioned generated databases, which we define as: Db7251, Db326, and Db11. Using the “Analyze results” functionality, we obtained 3 bar-charts and, respectively, 3 tree-map charts that show the top 10 WoS Categories (specific fields of study) that the included papers in each database are subscribed to. The functionality allows selection between 5, 10, 15, 20, and 25 results per chart/map, but we considered that 10 items are sufficiently relevant and easy to visualize. These graphs can be found in Supplementary Materials (Figures S2–S7). For better integration of these data, we designed Table 1, which presents the dynamics of these granulated research areas (WoS categories) between the 3 databases. It is obvious that the narrower the search, the more niche and more expensive domains (Nanoscience, Polymer Science, Cell Biology) are included or assigned higher ranks, while other more general areas disappear (Physics Applied, Material Science Multidisciplinary, Pharmacology Pharmacy) or are assigned a lower position in the hierarchy (Material Science Biomaterials). These observations indicate that there are probably notable relevant results in niche domains regarding our search that we should investigate further than Db11, but also which broad and narrow academic fields are connected to the study of biomaterials.

**Figure 2:**
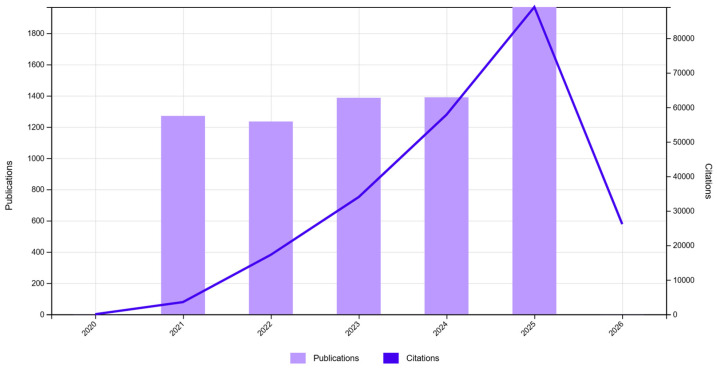
Evolution of publication numbers and citation trends for Db7251 in the 2021–2025 interval.

**Figure 3:**
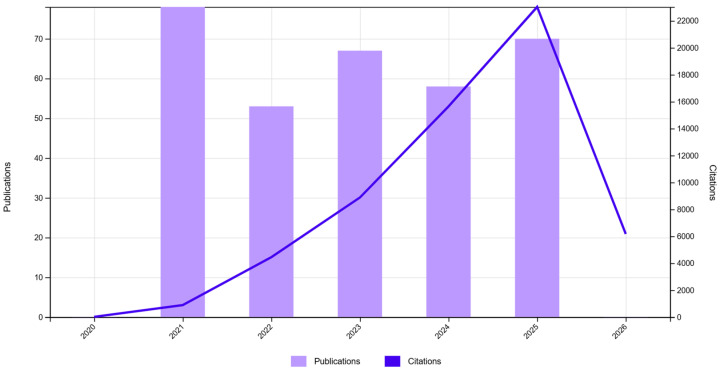
Evolution of publication numbers and citation trends for Db326 in the 2021–2025 interval.

**Figure 4:**
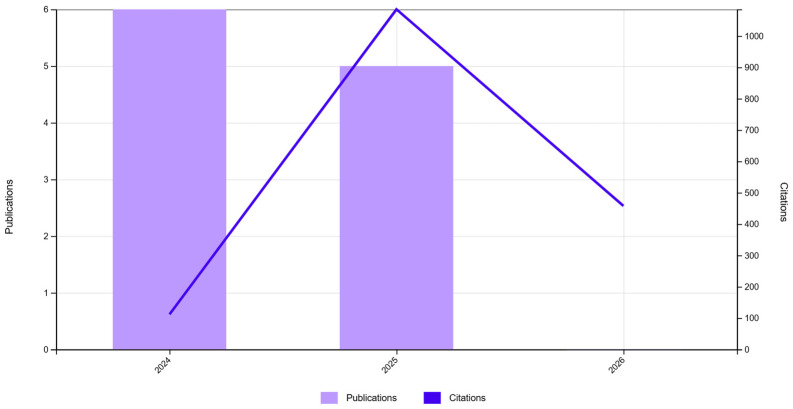
Evolution of publication numbers and citation trends for Db11 in the 2021–2025 interval.

The “Citation report” functionality shows, simultaneously, the evolution of scientific production and that of the citation index in the selected time interval (2021–2025) for the three databases. As one can infer from Figure 2, Figure 3 and Figure 4, the scientific production in the biomaterials field grew significantly in 2025 compared to the 2021–2024 period (Figure 2), and since the same cannot be observed for reviews (Figure 3), this could mean that this was a prolific time interval for experimentation with biomaterials in the scientific world, which could mean that many advancements were made in obtaining viable applications. The graph for Db11 (Figure 4) does not offer much information, except for the fact that until 2024, it appears that there were not too many highly cited papers in this domain, which gives us a starting point of relevant, probably applicable, scalable, or translational results bursting into the biomaterials field.

In the last part of this bibliometric study, we used the VOSviewer software (version 1.6.20) to generate keyword co-occurrence maps for each of the three databases. The first attempts to obtain such bibliometric maps led us to an intermediate curation step that involved generating a thesaurus file in order to avoid duplicates (different spellings, singular–plural forms, synonyms, or syntagms with the same meaning, e.g., nanotechnology–nanoparticles–nanotubes–graphene oxide–nanofibers–nanocarriers = nano). Each map was generated by selecting the following options from the VOSviewer software:

- Create a map based on bibliographic data.
- Read data from bibliographic database files.
- Select the .txt files with the database, downloaded from WoS.
- Apply the thesaurus file.
- Apply a threshold (minimum number of occurrences of a keyword in the files from the respective database).
- Determine whether or not to keep all the keywords that meet the threshold.

We present below the obtained co-occurrence bibliometric maps, which were quintessential in determining what points the present review should address in order to cover some gaps or under-discussed subjects in the chosen field of research.

**Figure 5:**
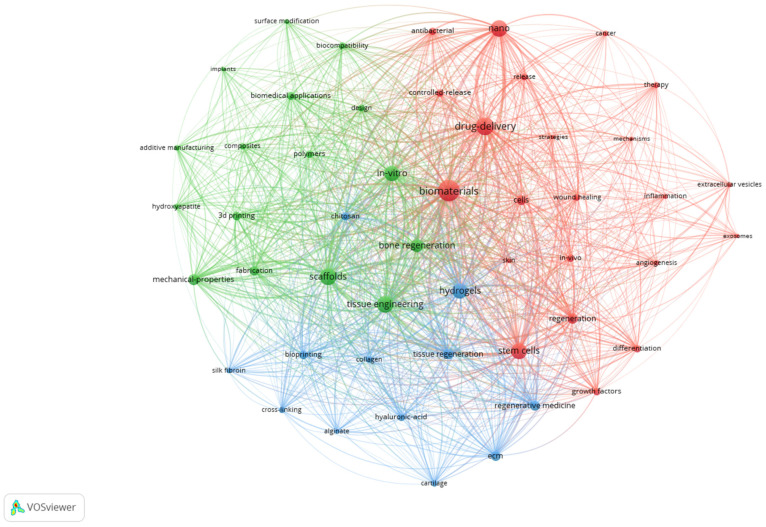
Keyword co-occurrence network map for Db7251.

For the 7251 publications in the database, the keyword co-occurrence threshold was set to 138, because we aimed to have a maximum of 50 keywords in each map for better visualization and interpretation. Using the 138 threshold in this case, out of 23,479 keywords, exactly 50 met the co-occurrence threshold. This setting generated a network map with 50 items grouped in 3 clusters with 1193 links (Figure 5). The automated view was rotated 180° for better visualization.

Cluster 1 (red) in the below network map contains 22 items revolving around the drug-delivery, stem cells, and nano concepts, so it is consistent with the biomedical–pharmaceutical direction.

Cluster 2 (green) contains 16 items referring mostly to scaffolds, materials, material properties, and technologies, so it is an engineering-oriented cluster.

Cluster 3 (blue) contains 12 items focused on hydrogels, natural polymers, and regeneration, so it is a bridge between a chemical and a biological approach to the subject, which is consistent with its position between Cluster 1 (biology-oriented) and Cluster 2 (chemical engineering-oriented).

The network map is useful for observing the connections between different items (keywords) and the strength of their link.

**Figure 6:**
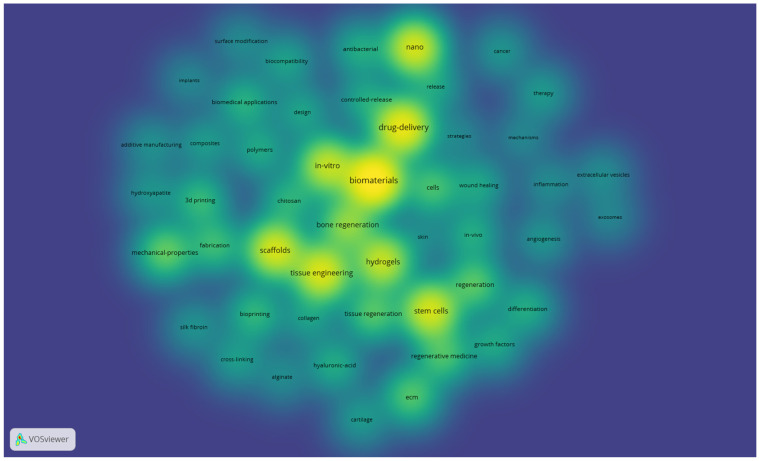
Item density map for Db7251.

**Figure 7:**
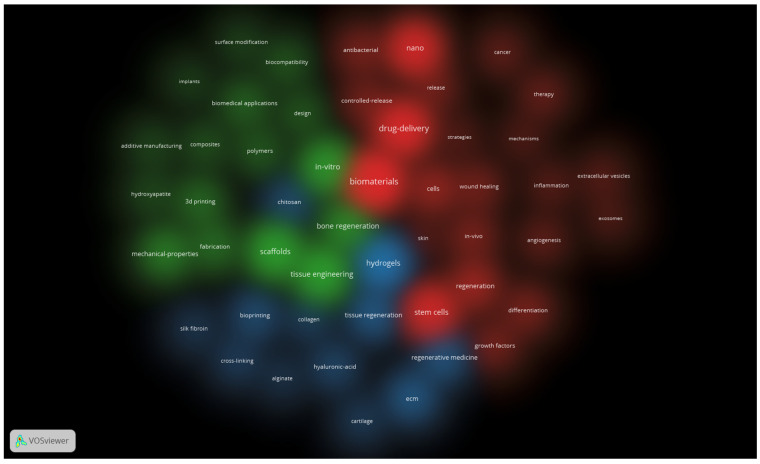
Cluster density map for Db7251.

The same settings also generated an overlay map (not presented) and 2 density maps: one for items and one for clusters (both presented below—Figure 6 and Figure 7). These density maps are useful to observe the proportionality of occurrence between the terms (the more intense, the higher the occurrence of the keyword in the item density map) and the distribution of the clusters (how much a certain cluster takes up in the entire map, which indicates which field of study is addressed more and which one is still in its infancy).

Figure 6 shows that tissue engineering, drug delivery, nanotechnologies, scaffolds, stem cells, and hydrogels are quite common subjects in the selected literature, whereas bioprinting, cancer, therapy, regenerative medicine, and other related subjects are not as developed.

Figure 7 allows us to observe that the biological approach has the biggest proportion, followed by the engineering one, and that intersecting domains like tissue regeneration–regenerative medicine are just beginning to emerge. It is interesting to see how the term “chitosan”, although integrated into the third cluster by its links, is positioned inside the second cluster, showing how a certain concept can cross the barrier of one particular domain.

Similar maps were generated for Db326 and for Db11 and are presented below.

For consistency, all maps were generated using the same thesaurus file, and in each case, we aimed to include a maximum of 50 keywords for better visualization and to facilitate the comparison process between the same type of map generated for different databases.

**Figure 8:**
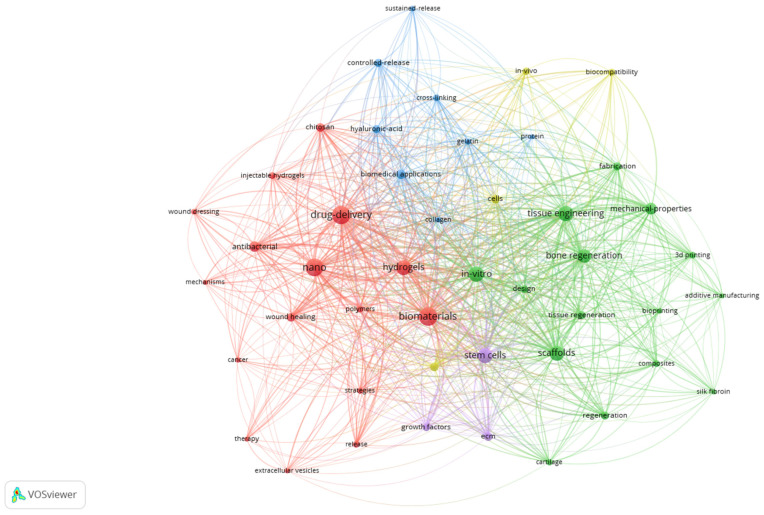
Network co-occurrence map for Db326.

For the Db326 network map (Figure 8), the co-occurrence threshold was set to 8, thus obtaining 46 items that met this condition, out of 2276 identified keywords. The 46 items are grouped in 5 clusters and present 730 links.

It appears that, as the database becomes more restricted (filtered), more specific keywords appear, and thus more clusters define niche subjects connected to the central theme.

Cluster 1 (red) contains 16 items and is focused, as in the case of Cluster 1 from Db7251, on drug delivery and nanotechnology. However, what is different from the first database here is that this cluster refers to more advanced concepts, such as wound healing, cancer, injectable hydrogels, and mechanisms (so it is an in-depth approach compared to Cluster 1—Db7251).

Cluster 2 (green) contains 15 items grouped around scaffolds, in vitro, tissue, and bone regeneration, so it maintains the engineering approach, but it also brings in biomedical applications, compared to Cluster 2 from Db7251.

Cluster 3 (blue) contains 8 items referring to materials and their properties, and it definitely creates a material/chemical-oriented bridge between Cluster 1 and Cluster 2.

Cluster 4 (yellow) contains 4 quite dispersed items that cross over into the biological approach: cells, biocompatibility, regenerative medicine, and in vivo.

Cluster 5 (purple) contains only 3 items: stem cells, extracellular matrix (ECM), and growth factors, and it seems to be a very specific subsection of the large biomaterials domain, connecting (by its position) all the above clusters of Db326.

**Figure 9:**
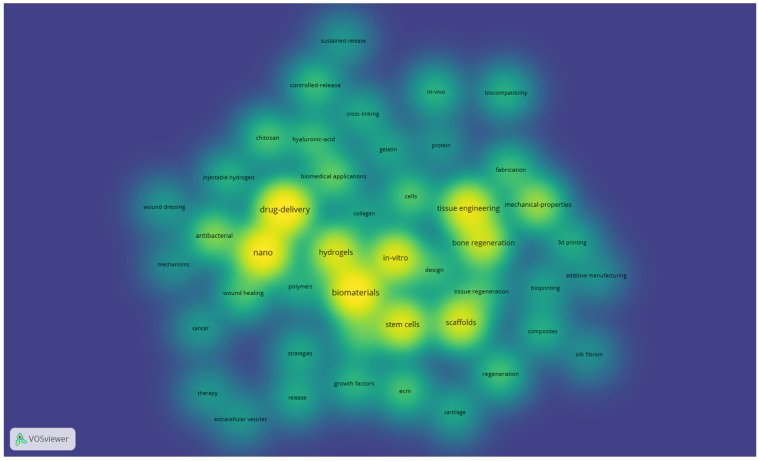
Keyword co-occurrence item density map for Db326.

**Figure 10:**
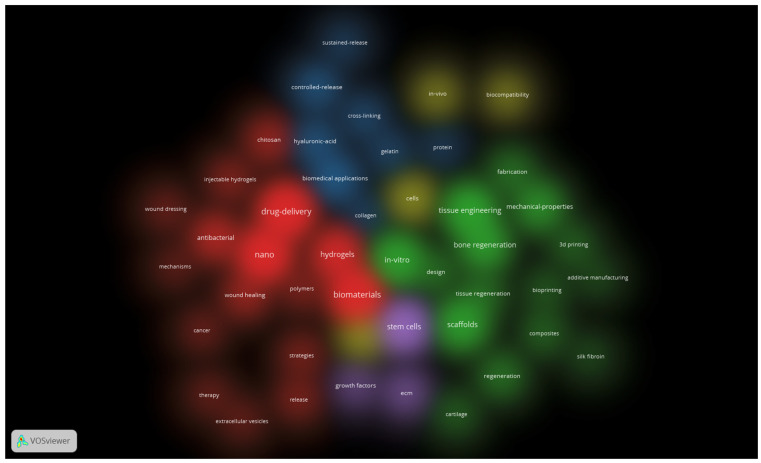
Keyword co-occurrence cluster density map for Db326.

The item density and the cluster density co-occurrence maps for Db326 show, respectively, a tissue-engineering–bone regeneration–drug-delivery–nano focus (Figure 9) and the fact that the emerging research trend seems to be directed towards regenerative medicine and in-depth biological studies of biomaterials connecting engineering and cell biology techniques (Figure 10).

**Figure 11:**
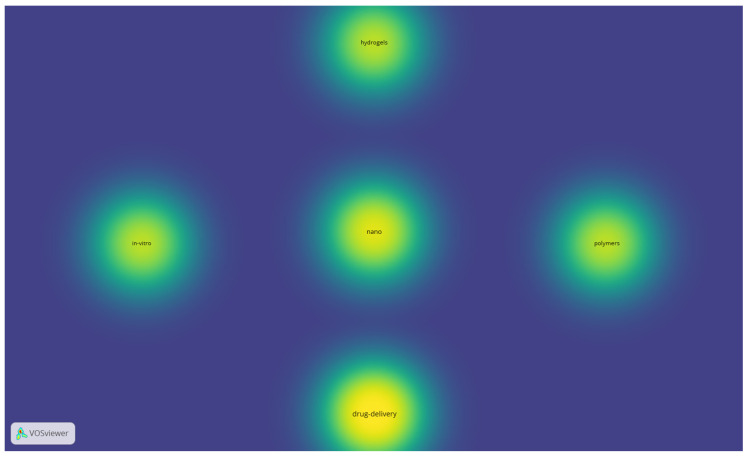
Keyword co-occurrence item density map for Db11.

**Figure 12:**
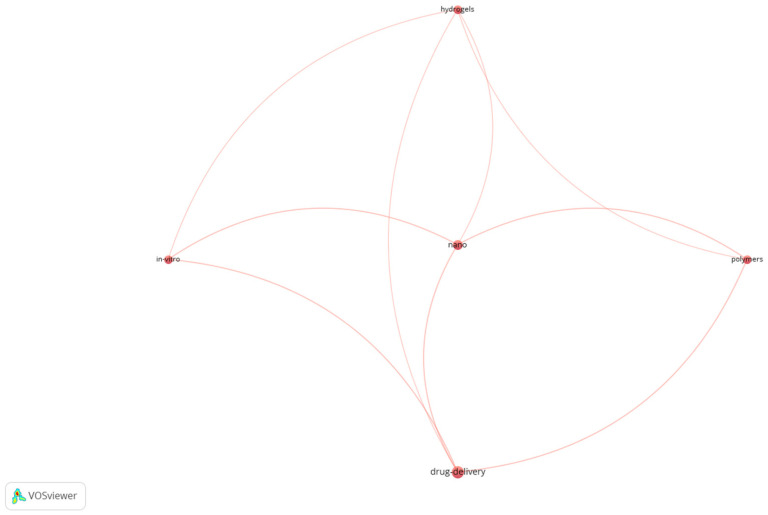
Network map for Db11.

Last but not least, the keyword co-occurrence bibliometric maps for Db11 indicate the terms that keep occurring even in extremely niche searches regarding the biomaterials’ domain, including drug delivery, nano, polymers, hydrogels, and in vitro (the terms were enumerated from the highest to the lowest occurrence based on the item density map—Figure 11). The connections that link these items can be observed in Figure 12.

For Db11, the co-occurrence threshold was 3 (default), and out of 112 identified keywords, only 5 met the condition, generating only one cluster, which is why the item density and the cluster density maps look the same (we present only the item density map).

Summarizing the above bibliometric study, one can identify well-developed academic research areas in the biomaterials field, such as nanotechnology, hydrogels and polymers, stem cells, scaffolds, drug delivery, in vitro studies, and bone regeneration, but also insufficiently addressed areas, such as regenerative medicine and the emerging TERM domain, theragnostics, regeneration of other tissues besides the bone system, wound healing and wound dressing (in relation to biomaterials), cancer, drug testing, therapies and personalized medicine, drug delivery in relation to the cellular microenvironment, tissue engineering components, and bio-fabrication technologies, as well as in-depth biological approaches to the above-mentioned fields. This distinction represents the conceptual foundation upon which the present analysis aims to systematically and coherently highlight the less visible scientific reference points that underpin the field of biomaterials. The methodological approach involved organizing relevant information into analytical categories, critically evaluating the specialized literature, and integrating the findings into an interpretive framework that facilitates a deeper understanding of the mechanisms and principles governing the development and application of biomaterials.

2. Search in other databases and web pages for in-depth, explanatory, and complementary information

**Figure 13:**
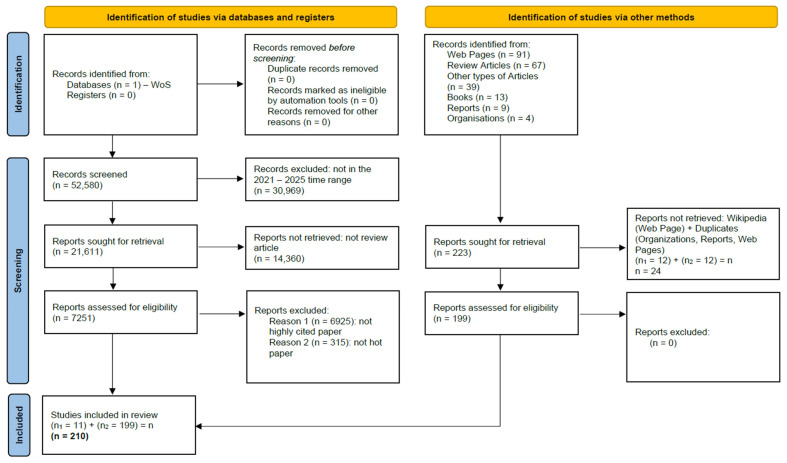
PRISMA flow diagram.

As we present in the PRISMA flow diagram below (Figure 13), for an in-depth approach and to shed light upon topics insufficiently developed in the bibliometric study of selected literature, we conducted an extensive search in databases other than WoS (PubMed, Elsevier, etc.) and in other electronic resources, such as research or health organizations’ web pages, product brochures from companies in the field, original data articles and other review articles in the biomaterials domain, and reports from market research companies that address our proposed subject. The number of identified, screened, and included studies based on specific criteria, both from the WoS database and outside of it, can be observed in the diagram; for studies from WoS, the inclusion criteria were presented previously in this chapter, and no other exclusion criteria were applied, whereas for studies outside the WoS database, exclusion criteria were applied, since the search was very specific. Wikipedia as a source and also duplicates of web pages reached from different searches were excluded. Overall, 220 bibliographic resources were used (included) in the present review: 11 from the WoS-generated bibliographic database and 209 from other specific searches.

3. Integration and new output

The main goal of this research was to generate a material that can be used as a research tool. Therefore, by integrating the previously mentioned studies, we generated comprehensive tables in which any researcher, whether now approaching the field of biomaterials for the first time or already having extensive experience, can find categorized and filterable information to use either as an answer to targeted questions or as a starting point for other studies or comparisons. In addition, the chapters that we have defined emphasize the identified research gaps, trying to extract as much information as possible from the existing literature on these less addressed topics.

## 3. Biomaterials and Computational Material Science Databases

In the process of using biopolymers for various biomedical applications, the first step is to identify the optimal molecules. Searching for and comparing the different properties of these materials, either virtually or in the laboratory, is a challenge. In recent years, databases with biomaterials have been developed, which are summarized in Table 2 and detailed in the Supplementary Materials (Table S1). All the included databases are currently active (2026), and for each, it is indicated whether artificial intelligence (AI) or machine learning (ML) have been integrated into its design.

The databases provide comprehensive and detailed collections of properties for a wide variety of polymers, both natural and synthetic. Typical applications and processing grades for each main polymer are easily accessible, making the databases valuable tools for researchers in both small laboratories and industry. More complex structures, such as multilayer films, nanomaterials, electro-optical, and electronic devices, are being developed in contemporary polymer science and engineering. These architectures require more advanced testing to evaluate end-use performance. Additionally, researchers can use the available databases to design and customize polymers and complex polymeric structures to satisfy certain end-use performance requirements by modulating the structure–property interactions of polymeric materials.

In addition to databases with ready-to-use materials, such as those mentioned above, the digital era also provides platforms for computational simulation of materials, in silico development, and digital testing in virtual environments that simulate, at the molecular level, the thermodynamic conditions and chemical interactions of real environments. These platforms (examples are listed in Table 3—all currently active) are emerging products of computational chemistry and computational materials science. They now serve as essential tools for developing advanced materials with product-targeted properties, for drug discovery and drug design, and for education.

The benefit of dynamic visualization tools is that they make it easy to obtain various significant analyses from the prospective data. This facilitates the exploration and exploitation of opportunities for innovation and knowledge management, as well as the appropriate communication of analyzed information to researchers.

## 4. Important Applications of Biomaterials in Modern Medicine—Smart Products for Diagnostics, Treatment, and Theragnostics

Biomaterials have proven to be highly valuable not only in diagnostic platforms—such as biosensors designed to detect and quantify specific analytes, including those used in blood glucose monitoring devices—but also in therapeutic and theragnostic applications addressing a broad spectrum of clinical conditions, including post-traumatic tissue loss defects, complex post-surgical reconstructions, and tissue damage resulting from severe infections or necrosis.

### 4.1. Wound Dressings

Wound healing is a complex, dynamic process that researchers and clinicians worldwide strive to control as effectively as possible to prevent the consequences of interruption or cessation at any stage, which can result in loss of life or severely diminished quality of life. Skin diseases affect several million people globally, with 1–2% in developed countries suffering from chronic wounds that are difficult to heal. Currently, there are about 3000 wound care products available. Conventional dressings (bandages, gauze) are not effective for healing complex wounds (such as extensive burns, diabetic foot ulcers, or infected wounds), which is why new solutions are being developed. These new approaches combine biomaterials (biopolymers), bioactive molecules, and cells within the framework of skin tissue engineering [46,47]. These solutions can be broadly classified as acellular or cellular (allogeneic or autologous) skin substitutes, each further categorized as temporary (removable), temporary (biodegradable, bioresorbable), or permanent (integrating graft). Supplementary Material Table S3 presents a list of such products, already marketed or in clinical trials worldwide. This table was inspired by Boyce et al. [48] and Snyder et al. [49]. Acellular scaffolds can also be combined with both allogeneic and autologous cells, with the latter combination being the most effective.

**Figure 14:**
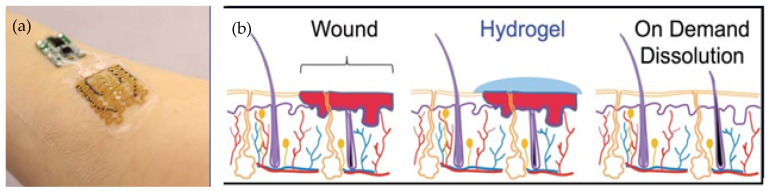
(**a**) Slim and flexible smart wound dressing with a range of pH sensors, thermo-receptive drug vectors, and a controller. Source: Khademhosseini Lab, Harvard–MIT. (**b**) Dissolvable hydrogel for burns, which promotes wound healing. Source: Grinstaff Laboratory, Boston University [50].

Regarding the acellular skin substitutes, there are also others worth mentioning:

- Smart dressing for the treatment of chronic diabetic ulcers (Figure 14a): it provides oxygen and trophic biochemical factors to blood vessels while monitoring healing. This is a theragnostic solution because it combines treatment with healing monitoring. By combining electronics, wound healing, microfabrication, biomaterials, and drug delivery, the dressing integrates sensors and actuators in close contact with the skin [50,51,52].
- Dissolvable dressing for burn treatment (Figure 14b): a self-dissolving hydrogel dressing that provides a barrier against infections and promotes healing, preventing tissue trauma caused by repeated removal of traditional dressings. As it dissolves into safe by-products in a controlled manner, the hydrogel allows the dressing to be removed on demand and the wound to be re-exposed without mechanical debridement or cutting, resulting in easier and less traumatic treatment [50,53,54].

As can be observed, recent advances in biomaterials have profoundly transformed the management of acute and chronic wounds, offering increasingly sophisticated solutions that overcome the limitations of traditional dressings. Bioactive materials, smart hydrogels, nanostructures, and skin substitutes provide not only mechanical protection but also targeted therapeutic interventions, stimulation of tissue regeneration, and continuous monitoring of the healing process. The integration of advanced technologies such as flexible microelectronics, controlled-release nanocarriers, and stimuli-responsive biomaterials opens new directions for designing biomaterials with superior biocompatibility, biodegradability, and intelligence, capable of dynamically interacting with the wound microenvironment and providing truly personalized solutions in regenerative medicine. Although these developments are remarkable, the specialized literature emphasizes the need for robust clinical studies, standardized protocols, and optimized production costs to facilitate large-scale adoption. These innovations promise to reduce complications, shorten healing times, and improve the quality of life for patients with complex wounds.

### 4.2. Nanocarriers and Other Solutions for Theragnostics

**Figure 15:**
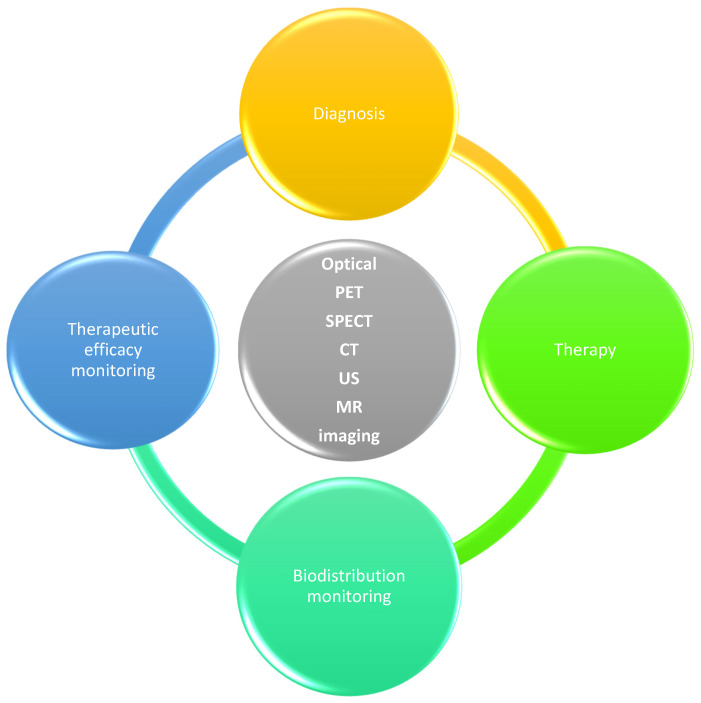
Ensemble of biomedical imaging techniques used in theragnostics. Positron emission tomography (PET), single-photon emission computed tomography (SPECT), computer tomography (CT), US (ultrasound), and magnetic resonance (MR).

In simplified terms, theragnostics refers to the use of a single, selective method to detect, monitor, and treat a condition [55]. In its modern definition, theragnostics is a medical approach that combines, in the same system, diagnostic capability (imaging), targeted and selective therapeutic delivery, monitoring of the therapeutic response (imaging) [56,57], and assessment of the biodistribution of the therapeutic agent (imaging), as illustrated in Figure 15.

This concept was first developed in nuclear medicine [58], when an isotope of iodine was used both to detect thyroid cancer (iodine accumulates in thyroid tissue through normal metabolic processes) and to destroy cancer cells through irradiation. Since then, theragnostics has evolved into complex platforms based on specific ligands that serve as therapeutic targets (for example, iodine: radioactive iodine can be monitored through imaging by NMR), activatable contrast agents that release therapeutic agents in response to a stimulus (such as ultrasound), monoclonal antibodies conjugated with superparamagnetic iron oxide nanoparticles, and other nanostructures capable of transporting and releasing therapeutic agents in a controlled manner. In these systems, the imaging molecule and the therapeutic agent act synergistically: the antibody ensures specificity (specific binding at the site of action), while the nanoparticle serves as both a carrier of the active substance and as a tracer for magnetic imaging, enabling real-time monitoring of therapeutic efficacy.

**Figure 16:**
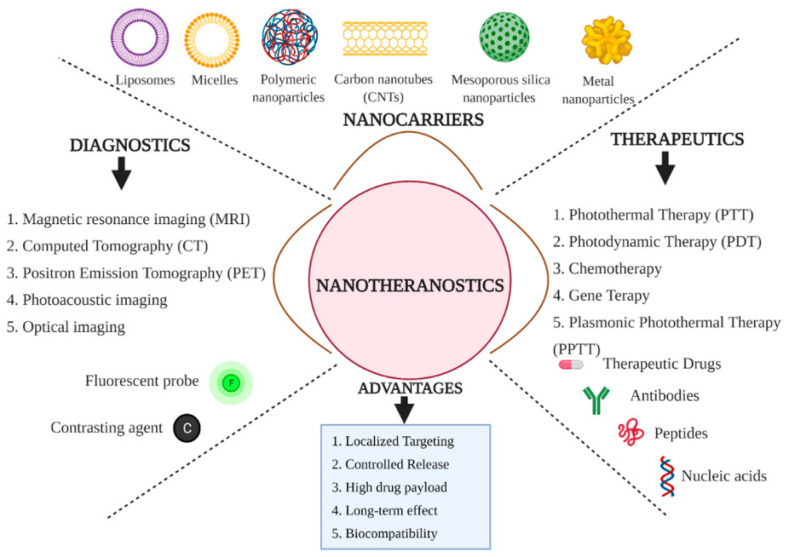
How nanocarriers can be used for both diagnostic and therapeutic purposes, as a single platform, which is the core principle of theragnostics. Reproduced with permission from [69].

Imaging techniques used in nuclear medicine for theragnostic applications include nuclear magnetic resonance (NMR), positron emission tomography (PET), and single-photon emission computed tomography (SPECT). Recent advances have expanded theragnostics beyond nuclear imaging to include photoacoustic and optical imaging [56]. In dermatology, for example, theragnostics involves the use of substances with specific binding to precancerous or cancerous cells and monitoring of the affected area using visible light upon UV illumination (photodynamic therapy) [59]. Optical imaging offers notable advantages: high sensitivity, real-time application, low cost, and safety due to the absence of ionizing radiation [60]. The defining feature of theragnostics is the targeted delivery of the therapeutic agent–imaging molecule combination. When a carrier vehicle is needed, this may be a nanoparticle (polymeric, silica, or metal), micelle, liposome, or carbon nanotube, typically surface-modified for specific binding. Theragnostics has proven effective in treating thyroid cancer and neuroblastoma, and in addition to oncology, it is now used in cardiovascular diseases, immunotherapy, neurology, and gene therapy. Co-delivery of diagnostic and therapeutic agents within a single system enables real-time validation of therapy through continuous, non-invasive monitoring [61,62,63,64,65,66,67,68]. This allows clinicians to rapidly assess treatment efficacy at the molecular or cellular level and adjust therapeutic strategies without waiting for a macroscopic effect, such as tumor shrinkage [60]. Molecular probes and nanoparticles used in cancer imaging and molecular therapy are relevant examples of theragnostic products incorporating biopolymers [50], and the diversity of carriers, therapeutic strategies, and diagnostic methods is summarized in Figure 16.

Overall, as shown in Figure 16, nanotheragnostics is one of the most promising directions in modern medicine because it integrates diagnosis, treatment, and monitoring into a single functional framework. The evolution from early applications in nuclear medicine to current platforms based on nanotechnology, advanced imaging, and targeted delivery demonstrates the remarkable potential of this approach to transform medicine into a more predictive, efficient, and patient-centered field. Therefore, theragnostics is a key step toward truly personalized medicine and the optimization of therapeutic outcomes in numerous pathologies.

### 4.3. Tissue Engineering (TE)

The most evident role of biomaterials in tissue engineering is to provide an architectural framework similar to the extracellular matrix (ECM) to promote cell growth and tissue regeneration. They generate the porous scaffold where the cells are seeded. Therefore, biomaterials must be able to deliver populations of cells and therapeutic agents, provide a structural scaffold with optimal mechanical properties for the tissue, present a (bio)degradation rate as similar as possible (ideally, identical) to the new tissue formation rate, and adapt to the growth and development of the surrounding tissue [70].

In turn, tissue engineering finds its applicability in regenerative medicine and in personalized medicine (the two specialties sometimes overlap), in the development of smart biomaterials, in creating drug formulations with targeted delivery and/or controlled or sustained release, in building reliable solutions for in vitro drug testing, and in manufacturing high-complexity wound healing products.

The applications of biomaterials and tissue engineering overlap, support, and determine each other, as can be inferred from the graphical abstract.

To obtain products representing TE applications, various strategies are used, based either on cell therapies, on support scaffolds that mimic the extracellular matrix (ECM) or the tissue’s microenvironment—the extracellular fluid (ECF)—or on a combination of the two. These strategies can be used for the design and production of therapeutic products in vitro for subsequent transplantation, or for their generation in situ, directly in the body (in vivo). The manufacturing of these products is accomplished either through a bottom-up or a top-down approach, depending on the destination of the therapy and the technological, financial, and know-how resources. During the last few decades, tissue engineering has revealed positive results in solving regenerative, functional recovery, and treatment problems for most systems and organs.

Since biomolecules, cells, tissues, organs, and the entire human body have complex structures capable of storing and transmitting information (DNA, RNA) and performing fine, carefully tuned functions based on feedback and biochemical communication both locally and at a distance, it goes without saying that a simple artificial prosthesis cannot fully and accurately replicate the abilities of the replaced part. Therefore, fabricating a precise substitute requires incorporating, as much as possible, all the relevant natural elements into its manufacturing. Consequently, our ability to integrate not only the macro-components (cells, matrix, signaling molecules) but also the structural and informational cellular microenvironment (micro-and nano-topography, sensitivity to physical and chemical stimuli, the combination of vascularization and multi-stratified tissue, and allographic gene expression) is crucial for a successful product [71,72]. This accurate integration is the current goal in the field of tissue engineering. However, this field serves as an umbrella that unifies the technical skills developed by researchers in many other disciplines. Advances in micro- and nanofabrication, as well as in genetic engineering techniques, contribute extensively to the development of TE products that integrate and evolve much more naturally with host tissue than traditional prostheses.

The tissues and renewable organs developed so far in this field include skin, bone, periodontal tissue, cartilage, trachea, blood vessels (arteries, capillaries), cardiac tissue, nervous tissue, endocrine tissue, lung tissue, liver, pancreas, and limbal epithelium (eyes) [70,73,74,75]. For the success of these products, collaboration among specialists in related fields is essential. The domains that tissue engineering integrates are engineering and bioengineering, materials science, digital technology, chemistry, molecular biology and cell biology, medicine, and genetics [73,75]. The defining characteristics of the TE field are precision, personalization (individual-specific results), responsiveness, effectiveness, innovation, interdisciplinarity, integration of science with technology, resilience, and researchers’ creativity [73].

**Figure 17:**
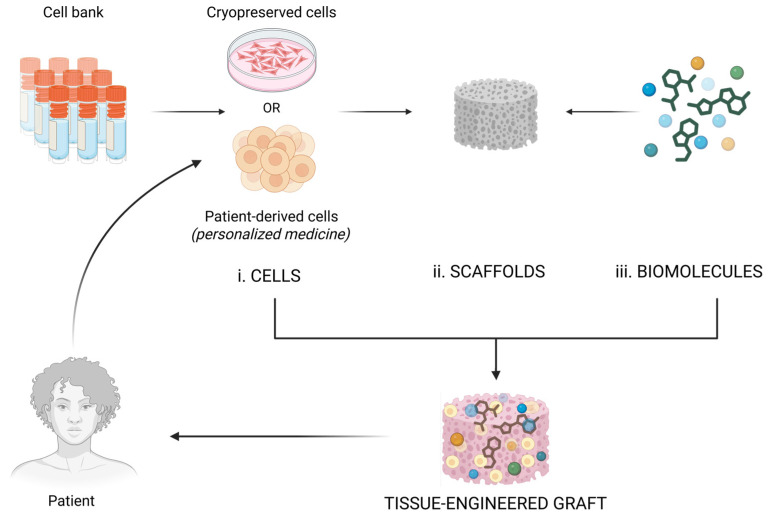
Tissue-engineered graft components. Created in BioRender. Badulescu, S. (2026) https://BioRender.com/6oq5ayt (accessed on 15 April 2026).

The fundamental elements necessary for the fabrication of tissues by tissue engineering are summarized below and schematically represented in Figure 17:

  i. Cells: from cell banks or patient-derived;
 ii. Scaffolds: for directing cell multiplication and organization (generally made of biopolymers);
iii. Biomolecules: growth factors, genetic reprogramming factors (including their delivery systems) [70,75,76].

**Figure 18:**
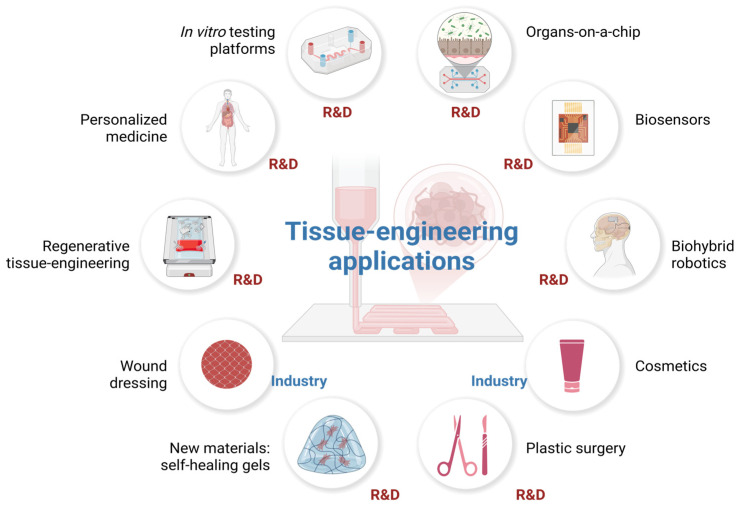
Tissue-engineering applications, from both research and industry areas. Created in BioRender. Badulescu, S. (2026) https://BioRender.com/hgk0wim (accessed on 15 April 2026).

**Figure 19:**
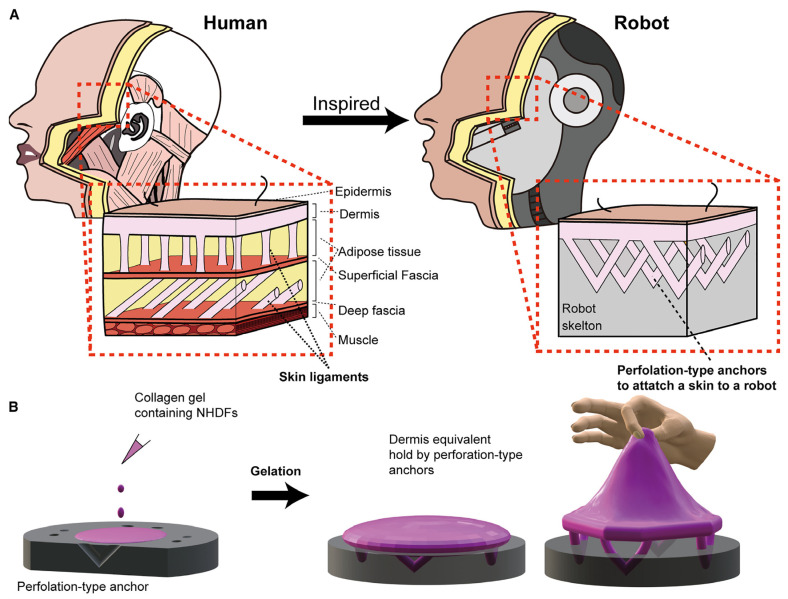
Multilayered skin obtained by tissue engineering. How the skin adheres to the complex structure of the robot, inspired by ligaments in human tissues [78]. https://creativecommons.org/licenses/by-nc/4.0/ (accessed on 15 April 2026).

The concept of tissue engineering has emerged in response to the challenges faced by modern global medicine: the inability to fully replicate the functions of natural organs, the inefficiency in controlling the progression of tissue damage when using mechanical devices (prostheses) in surgical reconstruction, the incompatibility and lack of adaptability of traditional prostheses to changes in the human body (such as growth and aging), the shortage of organs and tissues needed for transplants [75,77], transplant rejection due to immunological incompatibility, and the absence of previous solutions for irreparably damaged tissues for which traditional transplantation is not an option [73]. Driven by these urgent needs, the required solutions are, in start-up terminology, “pain-killers” (must-have) rather than merely “vitamins” (nice-to-have). The field has developed continuously, keeping pace with societal advancements and even anticipating future needs. Currently, the solutions offered address both research and industrial sectors, with a particular focus on biomedical applications, as shown in Figure 18. These solutions encompass both types of products and services mentioned above: those that address urgent problems and those that introduce innovations to improve or enhance human life:

- Wound healing;
- Regenerative tissue engineering and personalized medicine—to a large extent still experimental, expensive, and difficult to reproduce (transplantation of organs or tissues with a very high degree of biocompatibility or tissue regeneration in situ);
- Generation of flexible, modular, modifiable, controllable, accurate, and physiologically representative platforms for in vitro testing models;
- Organs-on-chips/tissue chips for pharmacokinetic, toxicological, and pharmacodynamic testing of potential new active substances;
- Biosensors using modified tissues for the detection of chemical or biological agents;
- Biohybrid robotics (biology and mechanical engineering): biohybrid android robots that move using biological muscle tissue, 3D printed flesh, skin with self-healing properties, attached to skin-ligament mimetic structures, which provide the flexibility and adhesiveness that are necessary for rendering facial expressions but also for preventing damage or detachment from the mechanical components (Figure 19) [78];
- Cosmetics and plastic surgery: developing materials—tissues that can best mimic the natural component—and identifying challenges in their fabrication (e.g., for skin: sterilization to avoid infection, addition of dry glands, pores, blood vessels, nerves, and fat to obtain thicker, wrinkled skin, ensuring fluent movement with the help of sophisticated actuators, addition of biosensors for quality control of the environment, and improved interactive capabilities) contribute significantly to the development of actual solutions in cosmetics (anti-aging products, corrective products) and in plastic surgery [78].

In tissue engineering, there are two general approaches, bottom-up and top-down, each with its own advantages and disadvantages [70]. For each application, depending on available data and resources (budget, equipment, expertise), the optimal method is selected.

In the bottom-up approach, mini-component parts of the final tissue or organ (cells, micro-tissues, scaffold materials, biomolecules) are brought together and assembled to generate the complex structure. This technique relies on the self-assembly and self-organization properties of the component parts and allows for precise design from the molecular or cellular level. The advantages of this approach are that the micro-architecture and composition of the resulting tissue are very well controlled and that heterogeneous, complex structures can be created. The main disadvantage is the difficulty of scaling to larger tissue constructs [70].

**Figure 20:**
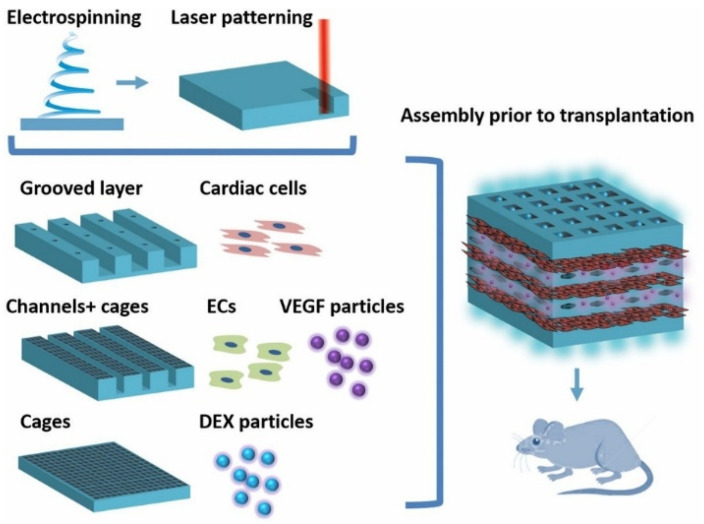
Steps and technologies used in the bottom-up approach to assemble a functional heart patch. Reproduced with permission from [79].

An example of the bottom-up methodology is provided by Fleischer et al. [79], who used it in cardiac tissue engineering to create functional cardiac patches (Figure 20). The group assembled a modular tissue consisting of several layers with distinct structures and functions to closely mimic the morphology and functionality of the heart and to integrate the patches with the target organ. Several biopolymeric porous scaffolds were treated differently to generate the aforementioned layers. The first layer, a matrix of albumin–electrospun fibers, was laser-shaped to create microgrooves that generate aligned fragments of heart tissue capable of propagating the anisotropic electrical signal. For the second layer, the scaffold was modeled with microchannels in which endothelial cells were seeded to form closed lumens. In the last layer, cage-shaped structures incorporating poly (lactic-co-glycolic acid) microparticle systems (PLGA) and delivery vectors that release vascular endothelial growth factor (VEGF), a vascularization promoter, or dexamethasone, an anti-inflammatory agent, were modeled into the scaffold. The structure, morphology, and function of each layer were characterized, and the tissue layers were grown separately under their optimal conditions. Before transplantation, tissue layers and microparticles were integrated into an ECM-like biological adhesive to form thick 3D heart patches. Finally, the patches were transplanted into rats, and their vascularization was evaluated. Due to the simple modularity of this approach, the authors believe that, in the future, it could be used to assemble other multicellular, thick, 3D, functional tissues.

The top-down approach uses a pre-existing complex material or scaffold modeled on the characteristics of the target tissue. Decellularized tissues or organs are often used, preserving the original ECM architecture and sufficient mechanical properties of the original tissue. This scaffold is repopulated with cells from the patient to restore tissue function and avoid rejection by the body.

Techniques used in the top-down approach include the electrospinning technique, which produces nanofibrous matrices that mimic the fibrous structure of the ECM and provide a conductive environment for cell proliferation, and the freeze-drying technique, which generates porous matrices with a large contact surface to promote nutrient diffusion and cell infiltration.

With the top-down approach, it is easier to obtain constructs that closely mimic the original tissue. Its main advantage is the use of natural scaffolds with inherent biological cues (molecular guides), while the main disadvantage is the difficulty in precisely controlling the microenvironment and integrating different types of cells and materials into the scaffold [70]. When considering the types of products that tissue engineering generates and the materials it uses, several technologies applied by this “pilot industry” can be distinguished [70], which are summarized in Table 4 below. For a better understanding of the principles and methods that characterize the main technology used in tissue engineering, namely bioprinting, we subsequently provide a detailed description.

Overall, the comparative analysis of biofabrication technologies (described in Table 4) highlights that there is no universally superior method, but rather a set of complementary tools, each optimized for a certain level of structural, functional, or biological complexity. Cell-based techniques—from the use of stem cells to organoids and self-assembly—offer high biological fidelity but limited geometric control, while additive biofabrication methods, such as 3D/4D bioprinting or laser-assisted techniques, allow for precise architectures but depend on biomaterials and may affect cell viability. In parallel, scaffold processing technologies (electrospinning, decellularization, freeze-drying, TIPS/DIPS, SC/PL) offer a wide range of options for obtaining porous, layered, or biomimetic structures, but with trade-offs between microstructural control, scalability, and shape complexity. Micro- and nanofabrication, along with lithography and microfluidics, complete the landscape with the ability to create extremely precise micro-architectures, which are essential for advanced tissue models or organ-on-chip systems. The differences between the techniques become much more evident when studied side by side: for example, organoids and self-assembly more closely replicate natural cellular organization processes than 3D bioprinting, but bioprinting enables large-scale structures with controlled geometry that are impossible to achieve through self-assembly. Similarly, electrospinning generates submicron fibers similar to the extracellular matrix but cannot reproduce complex macro-architectures, such as those obtained by stereolithography or FDM. TIPS and DIPS allow fine control of porosity, but SC/PL offers larger and interconnected pores, which are useful for bone implant engineering, while gas-foaming produces porous structures without solvents, but with less uniform pore distribution. Laser-assisted bioprinting (LAB) techniques outperform conventional bioprinting in cellular resolution, but microfluidics offers superior control over the dynamic environment and biochemical gradients. The technique of removing cellular components from tissues remains superior to any other when it is necessary to preserve the native architecture of an organ but is inferior to microfabrication techniques when the reproduction of specific microstructures is required. Therefore, the choice of the optimal technique depends on the necessary balance between biological fidelity, architectural control, scalability, and the final function of the construct, emphasizing the integrative and interdisciplinary character of modern tissue engineering.

Three-dimensional printing is a process that creates materials layer by layer. The process is automated, guided by CAD (computer-aided design) software, and belongs to the family of additive manufacturing technologies, in which objects are formed by adding material, in contrast to subtractive manufacturing, where material is removed by cutting, drilling, perforating, or grinding. The main difference between “additive manufacturing” and “3D printing” lies in their scale of application: the former is used at the industrial and commercial level and involves more operational processes beyond the actual manufacturing of the object, such as design, material traceability, workflow management, and quality control. The latter is applied on a small scale, at the prototype level, and is intended for individual users and start-ups, at most reaching pilot scale. The CAD software contains the digital model of the object to be printed and transmits information to the printer regarding the location and quantity of material to be deposited. Most 3D printers use polymers, but some also support metals or ceramics. The technologies used in 3D printing include fused deposition modeling (FDM), selective laser sintering (SLS), stereolithography (SLA), and PolyJet. For additive manufacturing, applicable methods include powder bed fusion (PBF), fused deposition modeling (FDM), and material jetting (MJ) [122,123,124,125].

Three-dimensional bioprinting (3DBP) is a form of 3D printing that uses biomaterials and biologically functional components, such as cells and biomolecules or signaling molecules. Printing is performed in a predefined framework, layer by layer, resulting in living tissue. Through 3DBP, microphysiological systems (MPS) are created, including organoids, organs-on-chips, tissues, and their combinations, which are used as implantable or ex vivo devices to improve function, replace lost tissues, study physiology, or test chemicals and drugs. Their use can serve therapeutic, diagnostic, or prognostic purposes. Micropathological systems (MPtSs) are MPS used to study diseases instead of normal physiology. They are biomimetic systems that accurately recapitulate the biological microenvironment [91].

Four-dimensional printing uses the same techniques as 3D printing but employs responsive materials, allowing the resulting structure to change its properties, shape, or even functions over time in response to applied stimuli. This makes it a dynamic construct, with time as the fourth dimension considered in its name [91].

Four-dimensional bioprinting involves the precise deposition of biomaterials and biologically active components into constructs that can undergo deformations and conformational or functional transformations over time when exposed to various stimuli. These constructs mimic the dynamic changes naturally found in the human body or alter their architecture to enable controlled release of encapsulated drugs, making them excellent for drug delivery. They are also ideal for bioactivation, wound healing, biosensing, and biorobotics [91,126].

Thus, 4D bioprinting produces dynamic bio-constructs that respond to stimuli and transform over time (smart), whereas 3D bioprinted structures remain static [127].

Tissue engineering applications have multiple facets that depend on each other, much like elements of an ecosystem. Scaffolds, advanced drug-delivery systems, regenerative medicine, and personalized medicine can each serve as either a component or the entire product in efforts to improve human health.

As (bio)materials used in tissue engineering, polymer matrices have three general functions [89]: they act as space-filling agents, delivery vehicles for bioactive molecules, and 3D structures (scaffolds) that organize and signal cells for tissue production or cell delivery (Table 5).

Within the last listed function (tissue scaffold), the matrices direct cellular behavior, tissue formation, and integration within the host organism [89]. To fulfill these roles, the scaffold (finite product) and the biomaterials composing it (biopolymers) must meet specific physical characteristics (mechanical resistance, crosslinking dynamics, porosity), physicochemical characteristics (degradation rate, surface chemistry, molecular diffusion, active molecule delivery: transport and release), and biological characteristics (biocompatibility, non-toxicity) tailored to each application or environment for which the scaffold is designed. For example, regarding mechanical properties, soft scaffolds are used for nervous and adipose tissue, while hard scaffolds are used for bone and cartilage tissue. Mechanical resistance influences mechanotransduction (the transformation of mechanical stimuli into biological signals), affecting gene expression and cell behavior. Scaffolds created for cell encapsulation must crosslink without damaging the cells, be non-toxic to both the cells and the tissue where they will be implanted, allow adequate diffusion of nutrients and metabolites to and from the encapsulated cells and surrounding tissue, and exhibit sufficient integrity and mechanical strength to withstand all manipulations necessary for implantation and in vivo operation. The degradation rate must match the rate of tissue formation to prevent collapse of the targeted tissue and to ensure proper integration and function. Peptides sensitive to specific enzymes can be incorporated to balance the durability of the scaffold’s architectural support with its degradation rate. The degradation products, like the biopolymers themselves, must be non-toxic, non-inflammatory, and easily absorbed and excreted by the body, as in the case of polylactic acid (PLA) and polyglycolic acid (PGA). The surface chemistry of biomaterials is critical because it influences protein adsorption, cell adhesion, and the medium- to long-term behavior of cells. For example, materials with binding domains such as RGD (arginine–glycine–aspartic acid) stimulate cell adhesion and, consequently, tissue regeneration. Surface modifications can be achieved by binding peptides or growth factors, which promote specific cellular activities; growth factors, in particular, should be released in a controlled manner to support natural processes of cell adhesion, growth, proliferation, and regeneration. Surface characteristics, such as topography and chemical functionality, influence the adhesion and incorporation of biomolecules (growth factors), and their optimization leads to improved cell–matrix interactions. Nanoscale scaffolds (scaffolds with nanoscale engineered surface characteristics) provide ECM-like topographic signals and have a higher surface-to-volume ratio, better supporting natural cellular behavior. In contrast, microscale scaffolds (scaffolds with microscale surface properties) better facilitate cell infiltration, nutrient diffusion, and vascularization, thus supporting tissue formation. The key to obtaining tissue that is both accurate and easily integrated is the harmonious combination of these two types of surface structures [70,77,128,129,130,131]. Many tissue engineering scaffolds initially fill a space occupied by natural tissue and then provide a framework for tissue regeneration. In this context, the physical properties of the material are essential to the success of the scaffold [77].

Examples of polymeric materials used as 3D supports (scaffolds) in tissue engineering [77,128,132]:

(a) Poly(lactic-co-glycolic) acid (PLGA) is an FDA-approved copolymer for medical devices and for use inside the human body. Its main disadvantages are hydrophobicity and processing in harsh conditions, which make it difficult to incorporate living cells and growth factors into the scaffold it generates.
(b) Poly glycerol-sebacate (PGS) is a polyester (biocompatible and biodegradable synthetic polymer) prepared by polycondensing glycerol with sebacic acid. It has a low synthesis cost, which makes it promising for the growing tissue engineering industry [133].

In addition, the mechanical properties and degradation kinetics of PGS can be tailored by controlling crosslinking time, crosslinking temperature, reactant concentration, and acrylate degree (in PGS acrylate) to meet application requirements. Due to the flexible and elastomeric nature of PGS, its biomedical applications are primarily focused on the replacement and engineering of soft tissues, such as heart muscle, blood vessels, nerves, cartilage, and retina. However, PGS applications have expanded to include drug delivery, tissue adhesives, hard tissue (bone) regeneration, and the design and manufacture of devices for applications that mimic native physiological conditions. New PGS scaffold designs range from honeycomb microstructures folded like an accordion to cardiac patches (PGS can generate porous, elastomeric 3D scaffolds with controllable stiffness and anisotropy; accordion-like folded honeycombs overcome the main structural and mechanical limitations of previous scaffolds, namely the inability to recapitulate cardiac anisotropy and to adjust flexibility, thus promoting the formation of aligned heart cell grafts and achieving mechanical properties more similar to native myocardium [134]), to gecko-inspired surfaces for tissue adhesives (gecko lizards can climb flexibly on a variety of natural surfaces due to the finely layered structure of their feet, which provides adhesion to any material—a structure that the scientific community has mimicked by creating dry adhesive surfaces [135], such as those based on PGS), and to PGS nanofibers for ECM-mimicking constructs [133].

(c) Hydrogels: The structural integrity of hydrogels depends on the crosslinks formed between the polymer chains, whether through chemical bonds or physical interactions. The advantages of hydrogels are that they are structurally similar to the extracellular matrix of many tissues, can be processed under gentle conditions (including physiological conditions), and can be administered in minimally invasive ways. In tissue engineering, they are used for delivering drugs and growth factors and for manufacturing tissue replacements.

A drug-delivery system is like a “prepaid molecular taxi” that transports active compounds safely, protecting them from degradation and releasing them at targeted sites. This enhances their efficacy and protects the body from additional adverse reactions that would occur if the medicine were not delivered specifically to its destination tissue. These properties address the typical drawbacks of conventional formulations, which lack specific targeting mechanisms and rely solely on the drug’s properties and its biodistribution process. For example, chemotherapeutic agents are highly toxic to every cell in the body, not just the tumor, and are generally small-molecule drugs that are rapidly cleared from circulation, making it difficult to maintain a therapeutic concentration at the site of action [136]. Overall, drug-delivery systems (DDS) have two major objectives, with two consequential effects: spatial and temporal control of drug delivery to maximize efficacy and minimize adverse reactions. Optimal dosing, controlled drug release, prolonged systemic circulation, and low toxicity are four characteristics of a successful drug-delivery system [137]. The controlled timing and location of drug release are achieved by modifying the drug’s kinetic and dynamic properties through the DDS formulation process, resulting in a more effective and safer outcome [136].

Drug-delivery systems (DDS) and tissue engineering (TE) are two interconnected fields. TE technologies can be used to tailor DDS by building scaffolds or multilayered constructs for controlled release, or by printing the entire system with encapsulated therapeutic agents. Meanwhile, DDS principles improve the results of TE products (scaffolds, tissues, or organs) by transforming them into platforms that respond to the environment and release growth factors, antibiotics, and other bioactive molecules that help the artificial construct integrate better into the body or serve a dual function: prosthetic (replacement) and therapeutic (antitumoral, regenerative, healing, anti-inflammatory, and so on).

In tissue engineering, the sustained, extended, and controlled release of active principles, signaling molecules, or growth factors is generally preferred, as these approaches can accelerate the regenerative process by maintaining a constant concentration of the desired molecule at the affected site over an extended period. They can also provide a timed release that aligns with the phases of cellular differentiation or stages of wound healing, while minimizing potential undesired systemic effects. The main concern and challenge is achieving an optimal balance between the release rate and the structural properties of the scaffold, which should not degrade before the new tissue has stabilized. Additionally, release patterns vary depending on the type of tissue: soft or hard. In soft tissues (such as liver, renal, cutaneous, skin, ocular, and others), high vascularization leads to rapid drug absorption and clearance, necessitating sustained or extended release. In contrast, hard tissues (bones, teeth) require controlled and/or localized (targeted) release to support processes like bone regeneration. Controlled release often employs nanoparticles or scaffolds to rapidly achieve an initial therapeutic concentration, followed by sustained release to maintain plasma concentration [136]. As previously mentioned, controlled release can also refer to targeted release [138], based on specific molecular interactions such as ligand-receptor binding, or to stimuli-based release (mechanical, pH, temperature, light, etc.), which can be achieved if the drug vehicle is formulated with a stimuli-responsive biomaterial. Controlled release considers both timing and location. In different contexts, it refers to drugs released over a specific period to ensure constant plasma concentrations, drugs released at a particular moment, or drugs released at a specific site identified by surface or immunological interactions—such as ligand–receptor biochemical bonds, antibody–antigen bonds (active targeting), or the enhanced permeability and retention (EPR) effect in tumors (passive targeting). These strategies enhance drug accumulation at the target site and improve therapeutic efficacy while reducing systemic side effects [136]. Targeted drug delivery, or smart drug delivery, refers to formulations that direct active substances to accumulate at desired locations in the body, primarily represented by nanomedicine. Nanotechnology has paved the way for developing new drug vehicles capable of transporting a wider variety of substances and delivering them with greater specificity and controlled release patterns [139].

Drug-delivery systems can be (A) hydrogel-based, (B) scaffold-based, (C) nanoparticle-based, or (D) cell-based, and they can also be completely natural, synthetic, or have elements from both areas.

i. Hydrogel-based DDS

One essential criterion affecting the capacity of polymeric materials (e.g., natural: cellulose, chitosan, hyaluronic acid (HA); synthetic: polycaprolactone (PCL), poly(lactic-co-glycolic acid) (PLGA)) to function as scaffolds in drug-delivery systems (DDS) is the drug’s release rate, which depends on their degradation mechanisms. Combining a polymer with a lipid improves the stability and controlled release of the delivery system [139]. Biodegradable polymers are promising gene delivery vehicles because they do not accumulate and have no toxic effects on targeted cells and tissues. Hydrogel-based dressings can deliver antibiotics and growth factors to enhance wound healing, and injectable hydrogels can deliver growth factors and cells to improve cardiac function.

ii. Scaffold-based DDS

Collagen-based scaffolds can deliver drugs, proteins, and genes. For example, Collatamp^®^-G is a collagen-based gentamicin delivery vehicle that provides sustained local delivery of antibiotics. Septocoll^®^ offers prolonged delivery by incorporating two gentamicin salts with different solubility rates. Collagraft^®^ is a biodegradable synthetic bone graft formulated as a composite of a collagen matrix and biphasic calcium phosphate. PerioCol^®^-CG and GDT PerioFocus are biodegradable chlorhexidine chips used in periodontics to provide sustained local delivery of the antimicrobial substance. The main drawback of collagen-based scaffolds is the immunogenic reaction to antigenic sites in the natural collagen molecule. Other disadvantages include the risk of infection when collagen is collected from xenogeneic sources and the degradation properties of the scaffolds. Genetically engineered collagen from Escherichia coli or yeast could address these problems, but this must become an industrially feasible solution.

Hyaluronic acid (HA) is known for its ability to promote wound healing through mesenchymal cell differentiation and its attachment to various cell receptors. Scaffolds made from this molecule can be used to deliver anti-inflammatory and antitumor drugs in a controlled and targeted manner.

Similarly, chitosan-based scaffolds are frequently used in the development of wound-healing, controlled-release applications due to the natural antimicrobial properties of the molecule. Hyaluronic acid and chitosan are also cationic molecules, so the release of substances included in their matrices can be pH-dependent.

Preclinical studies showed that hydroxyapatite-based 3D-printed scaffolds can deliver growth factors such as bone morphogenic protein (BMP) or platelet-rich plasma (PRP) and enhance bone regeneration. The same effect was observed with a hydroxyapatite/chitosan scaffold doped with bone marrow mesenchymal stem cells (BMSCs).

Synthetic polymers can also serve as starting points for drug vehicles that offer modified release, with advantages such as easier processing, improved mechanical properties, and customizable pore sizes and geometries of the fabricated scaffolds. For example, polycaprolactone (PCL) scaffolds have slow degradation rates, while polylactic acid (PLLA) and polylactic-co-glycolic acid (PLGA) degrade quickly. These two types of scaffolds are sometimes combined in different proportions to modulate the erosion rate or achieve a two-phase release profile (immediate release and sustained release). PLGA scaffolds are effective in delivering vaccines, antibiotics, and anti-inflammatory drugs due to their rapid degradation [139].

iii. Nanoparticle-based DDS

Nanomedicines are pharmaceutical agents that use nanoscale materials (1–200 nm) to deliver therapeutic small-molecule drugs to the site of action through targeted, smart, or controlled drug delivery. Nanomaterials enter cells via endocytosis, usually mediated by cell-surface receptors. Nanomaterials are typically positively charged and are therefore easily attracted to cells, which are negatively charged. Because endocytosis is mediated by lysosomes, nanomaterials must be resistant to low pH and lysosomal activity [137].

Polymeric nanoparticles (NPs) can be produced using techniques such as emulsification, ionic gelation, and nanoprecipitation from natural or synthetic materials and can take two forms: polymerosomes and micelles. Drugs can be encapsulated within the nanoparticle core, chemically conjugated to the polymer, or simply bound to the particle surface. The composition and surface charge of the particles determine the efficacy of drug loading and release [139].

Polymerosomes are artificial vesicles with membranes made of amphiphilic copolymers, offering excellent stability and cargo-retention efficiency, which are highly desirable for drug-delivery systems (DDS). They can also function as nanoreactors, protecting enzymes or proteins in nanometer-sized compartments and preserving their in situ functionality.

Polymeric micelles are composed of amphiphilic copolymers with a hydrophobic core and a hydrophilic shell, making them ideal for transporting hydrophobic drugs through aqueous environments. Their production involves dissolving the polymer in a solvent, followed by dialysis or precipitation using another solvent. Direct dissolution, solvent evaporation, and dialysis are methods for introducing drugs into polymeric micelles [139].

Both types of polymeric nanoparticles can have their surfaces modified or functionalized to enhance targeting capabilities and optimize bioavailability; however, their main drawbacks are the risk of particle aggregation and associated toxicity [139].

NPs constitute a significant portion of clinically approved nanomedicines due to their biocompatibility, stability, biodegradability, and multifunctionality, as well as their utility in targeted and controlled drug delivery. Examples of polymeric nanoparticles used in cancer therapy include Oncaspar (Asparaginase), a polymeric conjugate used in acute lymphoblastic leukemia; Eligard (Leuprolide acetate), a polymeric matrix used in advanced prostate cancer; Nanoxel (PTX), polymeric nanoparticles used in breast cancer, pancreatic cancer, and NSCLC; and Docetaxel PM (Docetaxel), polymeric micelles used in breast cancer, gastric cancer, NSCLC, ovarian cancer, prostate cancer, and squamous cell cancer [140]. Targeting specific sites of action can be achieved either passively or actively. Passive targeting involves coating the nanoparticle with a macromolecule that attracts water molecules through hydrogen bonding (PEG is often used), making the particle hydrophilic. As a result, the reticuloendothelial system (RES) does not process the nanoparticle (rendering it antiphagocytic), which extends its circulation time and allows it to diffuse into the desired tissues. Active targeting provides greater specificity through ligand–receptor or antigen–antibody binding. In cancer treatment, active tumor targeting involves functionalizing nanoparticles to recognize and bind to tumor-associated markers, while tumor-selective activation uses stimuli-responsive materials that release the active substance in response to internal signals from the local microenvironment (such as pH, oxygen levels, enzymatic activity, and redox potential) or external stimuli (such as phototherapy, ultrasound, or irradiation). The multiple tumor-targeted strategy combines active tumor targeting and tumor-selective activation to enhance therapeutic efficacy and minimize adverse reactions [140].

In terms of applications, nanoparticle-based drug-delivery systems are highly useful in combination with tissue engineering, for example, in treating bone cancer, trauma injuries, osteoporosis, and amputations. Ceramic nanoparticles made of PLGA, gelatin, collagen, and chitosan are most commonly used for bone regeneration. Bisphosphonate drugs, some of which promote osteoblast activity and others that decrease osteoclast activity, can combine with regenerating nanoparticles and bone minerals to treat osteoporosis [73]. Polymeric nanovehicles can also improve the efficacy of statin therapy [73].

iv. Cell-based DDS

Immunotherapy is a branch of the biomedical field in which drug-delivery systems (DDS) hold significant potential. There have been efforts to use DDS to initiate an immune response by introducing immune checkpoint inhibitors into the body [139]. Immune checkpoint inhibitors are monoclonal antibodies used in cancer therapy. Immune checkpoints are partner proteins located on immune cells, such as T cells, and on cancer cells, which recognize each other and prevent immune cells from killing tumor cells. This occurs because the proteins on cancer cells are the same as those on healthy cells, preventing the immune system from attacking the body’s own tissues. Immune checkpoint inhibitors block either the T cell receptors or the proteins on cancer cells that bind to these T cell receptors, thereby boosting the immune system to kill cancer cells. They have been used to treat lung cancer, breast cancer, bladder cancer, cervical cancer, colon cancer, head and neck cancer, Hodgkin lymphoma, liver cancer, renal cell cancer, skin cancer including melanoma, stomach cancer, rectal cancer, and any solid tumor that cannot repair DNA errors occurring during genetic material replication [141,142,143].

However, the risk of rapid clearance caused by immunogenic reactions to the materials used in DDS fabrication and concerns about long-term toxicity remain limitations, prompting the search for better solutions. The most promising alternative is the use of endogenous cells themselves, as they are natural vehicles for biomolecules in the body, transporting proteins, enzymes, and nutrients. Toxicity, immunogenicity, biological barriers, biocompatibility, and targeted delivery are much less significant or even nonexistent risks for cell-based drug-delivery systems. These characteristics make them ideal for antiviral and antitumoral therapy (e.g., leukemia, ovarian cancer). Red blood cells (RBCs), neutrophils, and dendritic cells (DCs) are cell types used in cell-based delivery technology [139].

**Figure 21:**
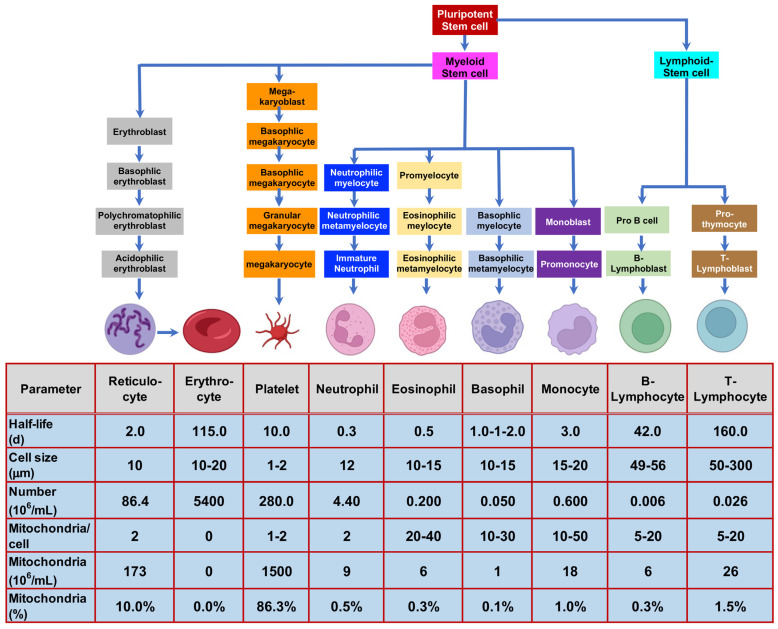
Blood cell characteristics and numbers. Reproduced with permission from [144].

RBCs are advantageous as drug carriers for several reasons: they lack nuclei, providing ample space for drug encapsulation; they circulate throughout the entire body; they have a long half-life (115 days) compared to most other blood cells—platelets (10 days), monocytes (3 days), reticulocytes (2 days), basophils (1–2 days), eosinophils (0.5 days), neutrophils (0.3 days); they are by far the most abundant [144], as shown in Figure 21; and they offer a large surface area for drug assembly. Additionally, the presence of surface markers such as CD47 protects them from phagocytosis. A challenge in using RBCs as drug vehicles is that surface conjugation of drugs reduces their elasticity and deformability, making them more susceptible to accelerated clearance by the reticuloendothelial system [139].

Neutrophils are blood cells that are chemoattracted to sites of inflammation, especially those caused by pathogen invasion, so their natural pathway can be very useful for drug delivery of anti-inflammatory or antimicrobial agents. Drugs are conjugated to their surface or internalized through co-culture methods [139].

Dendritic cells present antigens to T cells and secrete cytokines to promote T cell accumulation and enhance the killing activity against pathogens. Based on their natural functions, dendritic cells are used as cell-based drug-delivery systems to deliver antigens and activate the immune system in cancer [139].

i. DDS interaction with scaffolds

In tissue engineering, scaffolds serve two main purposes: (1) providing temporary support structures that facilitate cellular growth and organization, and (2) acting as vehicles for cells, drugs, and genes [136]. Responsive and autonomous biomaterials are extremely useful for developing controlled-release drug-delivery systems or scaffolds with this function. However, many scaffolds, especially porous ones, deliver biological agents primarily through passive mechanisms such as molecular diffusion, material degradation, and cell migration, rather than through active, dynamically regulated (stimuli-based) processes. The bioactive substances or therapeutic cells loaded into the scaffolds have a specific dispersion pattern (either even or discrete distribution), which must not disrupt the scaffold structure, and their release must ensure effective treatment over the desired period throughout the entire targeted area [136].

ii. DDS interaction with the extracellular matrix (ECM)

As with most components of living cells, the extracellular matrix (ECM) is a double-edged sword: on one hand, its mesh-like structure limits the penetration and distribution of active substances to their targets; on the other hand, it serves as a pool of targeting sites that can be used to facilitate drug delivery. One example of this adjuvant role is seen in the interaction between integrins, a family of cell surface receptors, and the ECM proteins to which they bind. Targeting integrins with ligands (drugs) can enhance drug delivery into the cell, a strategy that has been successfully applied in cancer treatment [136]. ECM-degrading enzymes, such as matrix metalloproteinases (MMPs) that cleave specific ECM proteins, represent a second way to improve drug absorption at the desired site. This has been achieved using liposomes loaded with such enzymes, also for tumors. Biomimetic materials, such as hydrogels made of ECM proteins or glycosaminoglycans that mimic the structures and properties of the ECM, are used as drug-delivery systems, especially in wound healing, tissue engineering, and cancer treatment [136].

iii. DDS interaction with cells

Cell-mediated drug delivery is particularly attractive for its therapeutic specificity and effectiveness, as other drug-delivery systems still face challenges in this area, despite the development of several surface markers and targeting approaches [136]. In cell-based drug-delivery systems, the therapeutic agent can be encapsulated within the cell, attached to the cell surface, or secreted in a controlled manner by a genetically engineered cell. Therapeutic gene networks are now used to develop highly advanced sensory and output devices, which will change how patients receive their medicine, especially those with chronic diseases [136].

Although preclinical studies present nanomedicine as very promising for tumor therapy due to improved pharmacology and toxicology of drugs [145], physiological barriers prevent nano-carried drugs from reaching their site of action in clinical studies, rendering them inefficient. In contrast, cell-mediated drug delivery offers high targeting efficiency and strong specificity; cells easily surpass various biological barriers, exhibit low immunogenicity, and have long circulation times in the bloodstream [136]. Therefore, combining nanomedicine and cell-based drug-delivery systems addresses the limitations of each approach.

For skin applications, various tissue engineering products have been developed as drug-delivery systems, such as patches, injectable devices, and subcutaneous implants, many of which focus on wound healing, prevention of inflammatory processes, and burn treatment [136].

To increase nanoparticle penetration through tissues, PEG- (muco-inert) and TAT-peptide- (cell permeation enhancer) coated nanoparticles were developed, which demonstrated improved traversal of artificial cystic fibrosis mucus and lung epithelial cells [136].

Regenerative and personalized medicine focus on creating tissues, organs, and medicines that are perfectly adapted or tailored to the recipient.

Regenerative medicine involves replacing or regenerating cells, tissues, or organs in the human body to restore their normal function. This branch of medicine includes methods such as cell therapy, gene therapy, tissue engineering, and biomedical engineering techniques, as well as more traditional treatments involving pharmaceuticals, biologics, and medical devices. It has enormous potential to cure diseases, improve quality of life, and generate significant economic benefits [146]. The common methods used to create products for application in regenerative medicine are:

1. Generating a scaffold to guide the architecture of the new tissue;
2. Activation of cells capable of initiating and sustaining regeneration with the help of growth factors (or genetic reprogramming factors);
3. Combining the two methods, in consecutive or simultaneous steps.

Personalized medicine is an emerging medical field that uses an individual’s genetic profile to guide decisions about the prevention, diagnosis, and treatment of their disease.

In cell-based therapy and in cell-based plus smart biomaterial scaffold therapy, tissue engineering has made significant progress in recent decades, with widespread applications across most biological systems.

For the skin, autologous skin substitutes have been developed, including bioengineered bilayered grafts that effectively cover wounds, stimulate regeneration, and reduce scar formation [73,74,75]. Products such as Apligraf and Dermagraft—the latter combining dermal tissue with an artificial epithelium—are already integrated into therapeutic protocols for burns and diabetic foot ulcers [73,74,75]. In parallel, 3D bioprinting enables the controlled deposition of cells and biomaterials, generating layered structures that closely reproduce the complexity of skin [70].

In the skeletal system and associated connective tissues, mesenchymal stem cells (MSCs) have become central to personalized orthopedic therapies, being used to treat fractures, bone defects, and osteoporosis, as well as in the development of bone and dental implants [70,73,75]. Tissue engineering has also enabled the regeneration of cartilage tissue, including the reconstruction of auricular cartilage and laryngeal tissue [73,75].

In the cardiovascular field, vascular grafts have been produced by 3D bioprinting or by concentric wrapping of vascular cells and fibroblasts on biodegradable tubular supports, which are then seeded with endothelial cells, without the use of synthetic materials [74]. Capillaries and cardiac patches based on fibrin or synthetic polymers such as PGS have also been created and used for the regeneration of post-infarction myocardium, with promising clinical results in reducing morbidity and mortality [70,73]. The functional maturation of these constructs is accelerated by electrical stimulation [70].

For endocrine diseases, functional thyroid structures capable of producing thyroid hormones have been obtained [74]. At the hepatic level, current therapies target both hepatic steatosis and the associated muscle atrophy [73]. Liver spheroids generated by combining polyurethane foam with hepatocyte cultures are also used in preclinical drug testing [74]. Bioprinting enables the fabrication of liver tissue with controlled architecture and the integration of a functional vascular network, using hepatocytes or stem cells [70]. In the visual system, tissue engineering has produced innovative therapies for glaucoma [73] and for limbal stem cell deficiency (LSCD), a condition characterized by the inability of the cornea to regenerate. Current strategies include autologous corneal epithelial cell culture (CACE), simple limbal epithelial transplantation (SLET), and the use of hydrogel or plasma-based contact lenses for in vivo culture of transplanted cells, techniques that are at various stages of preclinical and clinical validation [73,147]. For nervous tissue, therapies for peripheral nerve and spinal cord injuries have been developed based on scaffolds made from polymers such as PLGA and chitosan. These facilitate axonal guidance and support neuronal adhesion, combined with growth factors and neural or mesenchymal stem cells capable of differentiating into neurons and glial cells [70].

Smart biomaterials have also produced notable results in other systems, such as the respiratory system, where lung tissue substitutes are used in radiofrequency tumor ablation therapy, improving efficacy and reducing toxicity [73]. In the skeletal system, biodegradable scaffolds such as Pro-Osteon—a coral-derived graft—and composite polymer scaffolds offer effective solutions for fractures and bone defects, with extended lifespan and superior integration in orthopedic applications [74].

Overall, recent advances in tissue engineering and biomaterials demonstrate considerable potential for the development of personalized regenerative therapies capable of reproducing the structure and function of native tissues, thus contributing to the transformation of therapeutic paradigms in modern medicine.

## 5. Market View, Challenges, and Perspectives

The global biomaterials market is projected to reach USD 442 billion by 2035, with an expected annual growth rate of 7.9%, starting from USD 193 billion in 2019 [148]. This projection is supported by the increasing prevalence of chronic diseases and an aging population [2,149,150,151].

Personalized and regenerative medicine, along with advanced drug-delivery systems in the pharmaceutical sector, address the needs arising from these circumstances. As a result, more funding and greater effort are being invested in biomaterials [148], which are key elements in these healthcare sectors.

Despite this progress, there remains a gap in the bench-to-bedside translation of biomaterial-based products, tissue-engineered products, and individually tailored medicines, as well as in achieving the desired long-term stability and integration of biomaterials in the human body [148]. The development of increasingly advanced nano- and micro-fabrication technologies for biomaterial manipulation and customization is making these materials more versatile and effective. The growing number of start-ups and spin-offs (research-oriented start-ups) and the increasing trust they receive from both investors and the public are also positive factors in the effort to improve biomaterials’ properties and deliver innovative healthcare solutions more quickly to patients. These micro-companies focus on rapid growth and fast solutions to specific market needs, and they have a much shorter timeline from minimal viable product (MVP) to final product and actual sales compared to large, industrial, multinational companies. However, they usually operate at the pilot level rather than on an industrial scale, making them less procedural, faster, and more flexible—qualities that the biomedical field needs to accelerate the production and marketing of innovation-based solutions. Additionally, spin-offs are typically launched by academic research groups, so the team’s expertise is central to the company, which greatly benefits the research and development process. Once a spin-off has demonstrated market viability, it is usually acquired by a large-scale production company, allowing its products to be scaled up in mass production, quality management systems, automated production processes, and even applications.

Although biomaterials include metallic, ceramic, polymeric (natural or synthetic), and emerging materials, natural and emerging materials are currently trending [148] due to their increased biocompatibility and advanced functionalities.

The biomaterial industry has various application areas: cardiovascular (guidewires, implantable cardiac defibrillators, pacemakers, sensors, stents, vascular grafts, and others), ophthalmology (contact lenses, intraocular lenses, ocular tissue replacements, synthetic corneas, and so on), dental (bone grafts and substitutes, dental implants, dental membranes, tissue regeneration materials), orthopedic (bioresorbable tissue fixation products, joint replacement biomaterials, orthobiologics, spine biomaterials, visco-supplementation), wound healing (adhesion barriers, internal tissue sealants, skin substitutes, surgical hemostats, fracture healing devices), plastic surgery (acellular dermal matrices, bioengineered skin, soft tissue fillers, peripheral nerve repairs), neurology (cortical neural prosthetics, shunting systems, hydrogel scaffolds for central nervous system repair, neural stem cell encapsulations), and many others. Currently, the wound healing, plastic surgery, orthopedic, dental, and cardiovascular segments lead in market share by application area [148].

Companies manufacturing biomaterials-based products include BASF SE, Berkeley Advanced Biomaterials, Carpenter Technology, Collagen Matrix, CoorsTek, Corbion, Covalon Technologies, CRS Holdings, Dentsply Sirona, Evonik Industries, Invibio, Johnson and Johnson, Medtronic, Stryker, and Zimmer Biomet [148]. Major tissue engineering companies include AbbVie Inc., Acell Inc., Acelity L.P. Inc., Athersys Inc., Baxter International Inc., Becton Dickinson and Company, BioMed X, B. Braun, Cytori Therapeutics, Episkin, Integra LifeSciences Corporation, Medtronic plc, Organogenesis Inc., Osiris Therapeutics, ReproCell Inc., RTI Surgical Inc., Smith and Nephew, Stryker Corporation, Tissue Regenix Group, Vericel Corporation, and Zimmer Biomet Holdings Inc [150,152,153]. As observed, some biomaterial-focused companies are also active in tissue engineering. These companies pursue strategic collaborations, acquisitions, mergers, and product launches to secure a larger market share. For example, in March 2024, HVD Life Science partnered with Poietis to distribute Poietis’ NGB-R platform in Australia and Germany; in July 2024, PacBio partnered with Novogene to advance genomic research, including rare diseases and oncology, which benefit the tissue engineering domain; in January 2023, BioMed X and AbbVie extended their research partnership to focus on immunology and tissue engineering after an initial joint project on Alzheimer’s disease; and in May 2023, Canon acquired cell manufacturer Kyoto Seisakusho to enter the tissue engineering market [153].

The tissue engineering market is undergoing continuous transformation, driven by the development of next-generation biomaterials and scaffolds, technological advancements (such as stem cell technology, gene editing techniques, 3D-4D bioprinting, organs-on-a-chip, and microfluidics), as well as growing applications in various medical fields and increased funding from governments and private entities [153]. The tissue engineering market size is projected to reach unprecedented levels, not only due to technological advances and lower production costs, but also because the need for organ transplantation and solutions for chronic diseases is increasing. The global tissue engineering market size was estimated at USD 19.36 billion in 2024 and is anticipated to reach USD 43.13 billion by 2030, growing at a compound annual growth rate of 14.3% from 2025 to 2030 [153]. North America dominated the tissue engineering market with a share of 51.4% in 2024, followed by Europe, due to its advanced healthcare infrastructures and research facilities. However, Asia–Pacific was the fastest-growing market, supported by a well-funded healthcare system and government initiatives [150,152,153]. In terms of applications, the orthopedics, musculoskeletal, and spine segment generated the most revenue in 2024.

The end users in the biomaterials market include medical device manufacturers, pharmaceutical companies, healthcare providers (hospitals and clinics, including dental clinics), research centers, and universities [148]. All of these are expected to use biomaterials more intensively as research advances and greater focus is placed on minimizing adverse effects, providing tailored biomedical solutions for patients, and creating sustainable products, which is also a target of the 2030 United Nations Agenda for Sustainable Development [154].

To translate a biomaterial into a final biomedical product, several elements are required. Some of these are research-based, such as fabrication technologies or formulation principles, while others are regulatory-based, including quality management systems (GMP, GLP), as well as preclinical, clinical, and post-market studies. The regulatory elements remain the most time-consuming and costly, as research input is advancing more rapidly through collaborations among academia, industry, and research centers, as well as through open science publications and events. The translation process is undoubtedly a multidisciplinary journey, where commitment, agility, problem-solving, communication, innovation, and perseverance are essential for success. The effort to translate early-stage concepts into commercial products for clinical use is entrepreneurial in nature and requires a dedicated team of scientists, economists, legal, marketing, regulatory, and clinical affairs specialists driven by this specific goal. Finance and regulatory considerations need to be integrated into the product development strategy as early as possible, preferably from the beginning of the project, to ensure the process becomes a medical, research, and business success [155]. Although there are many financial, regulatory, intellectual property, and ethical challenges involved, the translation of laboratory discoveries into clinical therapies and marketable products is best achieved through strategic collaboration among academic institutions, research organizations, biotech companies, and even government bodies [148,150,152].

The real challenges encountered in the translation process lie in the market perspective, and scientists are not yet trained to think this way when studying or developing a new idea. The value proposition (what the product uniquely brings to the market, and what unsolved problem it addresses) and the associated business model (who buys and why) [155] are probably the first major milestones in bringing a product from the lab to the market. Many start-ups pivot their ideas when trying to answer these questions because they realize that the need they address is not necessarily well defined. Market studies and feedback on early MVPs help significantly with this process, and the sooner they are conducted, the more cost-efficient and time-efficient the process becomes. Next is the issue of profitability, and in many cases, the product can address more than one medical problem [155]. If the development team succeeds in identifying all the applications and benefits of their invention for all stakeholders involved (patients: different treatable conditions, different ages; caregivers: physicians, nurses, family members; governments: if the product could be reimbursed by a National Health Program), the product becomes more versatile and, as a result, has more potential buyers. In many cases, especially with innovations in the biomedical field, the regulatory environment must be adapted or changed [155], which requires substantial justification. Solid communication among researchers conducting pre-clinical and clinical tests, legal advisors, and auditors from regulatory national agencies is absolutely crucial. Even identifying the category in which the product falls (medical device, combination device, drug, biological product, cosmetic product) [156] can be a hurdle, and even when this is achieved, several factors must be considered for the tests and trials required for commercialization. Additionally, regulations differ from state to state, representing another challenge. Aligning requirements at the continental or even global level would greatly facilitate the translation process and help patients worldwide. Especially for orphan drugs or devices for unaddressed medical problems, it is advisable to start a conversation with international consortia, NGOs, and research societies, as they have contacts, databases, case studies, and generally more resources that, when combined, can build a stronger case or clarify whether the direction of such an effort is justified. Nonetheless, the energy of the families of potential beneficiaries of biomaterial-based products, as well as other innovative medical products, is immense, and these stakeholders have the power to influence decision-makers regarding the speed of the regulatory process or the financing of such projects.

## 6. Conclusions

Biomaterials are undergoing rapid integration in the biomedical field, where they are urgently needed to address medical conditions that place a significant burden on patients, their families, and healthcare systems (including caregivers, institutions, and governments). Among the problems they address, the most prevalent are chronic diseases (cardiovascular, orthopedic, dental, neurological, diabetes), traumatic injuries (burns, open wounds), the need for organs or tissues for transplantation, devices that better integrate into the body and are less prone to rejection, and medicines with fewer adverse reactions, improved patient compliance, and controlled, targeted release (such as in oncology).

Biomaterials are versatile materials that, depending on their generation, exhibit properties such as biocompatibility, injectability, responsiveness to stimuli, and autonomy (self-calibration). Moreover, they can be functionalized and tailored in various ways, making them suitable for diagnostic, therapeutic, and theragnostic tools (devices and medicines).

Their applications range from biosensors for wearable devices or bio-robotics to smart wound dressings, advanced drug-delivery systems (nanocarriers, scaffolds, hydrogels), and tissue engineering (artificial organs, organs-on-a-chip, tissue patches, surgical adhesives).

Not surprisingly, the market share for biomaterials has been growing in recent years and is expected to continue increasing significantly over the next decade, with many companies and governments investing in their research.

Advancements in tissue engineering technologies and cost reductions are helping to create more feasible products more quickly and on a larger scale. The deeper incorporation of personalized and regenerative medicine into medical practice is accelerating the integration of biomaterials into everyday clinical settings. Increased collaboration between academia, industry, and research centers, with a focus on business outcomes, facilitates a multidisciplinary approach to the creation and commercialization of these innovations, accelerating production, quality control, regulatory processes, and marketing.

Although the translation process from bench to bedside is not simple, more spin-offs and strategic mergers between spin-offs and multinational pharmaceutical or biomedical companies are occurring, and the gap between an idea and a final product is narrowing through shared work styles, expertise, accreditations, and resources.

## Figures and Tables

**Table 1:** Dynamics of the top 10 WoS categories in the generated bibliographic databases.

No.	7251 Publications (Db7251)	326 Reviews (Db326)	11 Highly Cited and Hot Reviews (Db11)
1	Engineering Biomedical	Chemistry Multidisciplinary	Nanoscience Nanotechnology
2	Materials Science Biomaterials	Nanoscience Nanotechnology	Polymer Science
3	Materials Science Multidisciplinary	Materials Science Multidisciplinary	Cell Biology
4	Chemistry Multidisciplinary	Engineering Biomedical	Chemistry Multidisciplinary
5	Polymer Science	Pharmacology Pharmacy	Materials Science Biomaterials
6	Nanoscience Nanotechnology	Materials Science Biomaterials	Biochemistry Molecular Biology
7	Pharmacology Pharmacy	Polymer Science	Biotechnology Applied Microbiology
8	Biochemistry Molecular Biology	Biochemistry Molecular Biology	Chemistry Physical
9	Biotechnology Applied Microbiology	Physics Applied	Medicine Research Experimental
10	Physics Applied	Chemistry Physical	Multidisciplinary Sciences

Legend: full underline = the respective domain disappears in the 3rd database; broken underline = the domain disappears in the 2nd database but reappears in the 3rd; yellow highlight = the domain first appears in the 2nd database; blue highlight = the domain appears only in the 3rd database. To make changes in the hierarchy easier to follow, each WoS category has a different font color. Exception: the three WoS categories that only appear in the 3rd database are all written with black font color. Observation: WoS categories represent highly specific academic fields used for bibliometric studies, which subscribe to broader, more general research areas, e.g., Nanoscience Nanotechnology (WoS category): Science and Technology—Other Topics (Research Area).

**Table 2:** Polymers, biopolymers, and advanced materials databases.

No.	Database Name and Link	Description	Type	Start Year	Uses AI/ML
1	Materiom www.materiom.org (accessed on 25 May 2025)	Materiom Commons—database for biobased materials Open-access	Start-up	2023	yes
2	BIOMATDB www.biomatdb.eu Database: biomaterialdatabase.com Marketplace: www.biomaterialmarketplace.com (all accessed on 25 May 2025)	Advanced Database and Marketplace for Biomaterials Open-access	Horizon Europe 2020-founded project	2022	unclear
3	Cloud Materials www.materialscloud.org (accessed on 25 May 2025)	Educational, research, and archiving tools; simulation software and services; curated and raw data in computational materials science Open-access	Swiss National Science Foundation and European Commission project	2020	yes
4	DEBBIE https://projectdebbie.github.io (accessed on 25 May 2025)	Database of experimental biomaterials and their biological effect Open-access	Horizon Europe 2020 Marie Sklodowska-Curie grant	2020	yes
5	NOMAD Website https://nomad-lab.eu/ Database https://nomad-lab.eu/prod/v1/gui/search/entries (both accessed on 25 May 2025)	Free web service (largest database) for browsing, sharing, organizing, analyzing, downloading, and publishing materials science data Open-access	German team	2014	yes
6	MatWeb www.matweb.com (accessed on 25 May 2025)	Searchable online free database of engineering materials and their properties Open-access	A team of engineers founded Automation Creations Company, and from that, MatWeb LLC was spun off	2011	yes
7	The Materials Project https://next-gen.materialsproject.org (accessed on 25 May 2025)	Computed information on known and predicted materials and powerful analysis tools to inspire and design novel materials Open-source	Multi-institution, multi-national efforts with a team formed of professors, researchers, engineers, and experts in materials science and engineering, computing science and operations, materials algorithms, data, and experimental validation	2011	yes
8	Genome Materials Initiative (MGI) www.mgi.gov (accessed on 25 May 2025)	This project aims to discover, manufacture, and deploy advanced materials twice as fast and at a fraction of the cost compared to traditional methods	The Materials Genome Initiative is a federal multi-agency initiative (part of the National Institute of Standards and Technology, US Department of Commerce)	2011	yes
9	PoLyInfo https://polymer.nims.go.jp -> part of MatNavi https://mits.nims.go.jp → part of DICE https://dice.nims.go.jp (all accessed on 26 May 2025)	The polymer database “PoLyInfo” systematically provides various data (properties, chemical structures, IUPAC names, processing methods of measured samples, measurement conditions, used monomers, and polymerization methods) required for polymeric material design	Japanese team of researchers	1995	yes
References [10,11,12,13,14,15,16,17,18,19,20,21,22,23,24]					

**Table 3:** Computational material science platforms.

No.	Database Name and Link	Description	Team	Start Year	Uses AI/ML
Molecular Design and Education					
1	Computer citrine https://citrine.io ! not free (accessed on 27 May 2025)	B2B SaaS platform with data management and virtual lab tools for developing new materials and chemicals used in virtually any industry. The company offers services covering sales, supply chain, scale production, and sustainability.	California-based enterprise	2013	yes
2	Schrodinger www.schrodinger.com ! not free (accessed on 27 May 2025)	Digital chemistry company: computational platform for molecular discovery and design: Materials Science: polymeric materials, metals, alloys, ceramics, pharmaceutical formulations and delivery, etc. Life Science: small molecule drug discovery, biological drug discovery Educational platform: materials science and life science, all paid certification courses.	Offices worldwide	1990	yes
3	VASP www.vasp.at/ (accessed on 27 May 2025)	The Vienna Ab initio Simulation Package (VASP) is a software for modelling atomic scale materials, e.g., electronic structure calculations and quantum–mechanical molecular dynamics, from first principles. It is based on quantum mechanical calculations; it does not use AI, but it can be used in conjunction with ML techniques. It can perform structure relaxation, functionals, magnetism, first derivatives, optical properties, linear response to electric fields and ionic displacements, many bodies perturbation theory, Berry’s phases, Green’s function method, and so on.	Research group at the University of Vienna	1983	no
4	AFLOW https://aflowlib.org/ https://materials.duke.edu/aflow.html (both accessed on 27 May 2025)	Automatic Flow for Materials Discovery is an open-source platform for high-throughput materials discovery, and it led to the creation of the largest materials database in the world, which is available on the website. It provides a variety of tools and modules to analyze structures and predict material properties, including autonomous error corrections for density functional theory calculations (using VASP), accurate crystal symmetry packages and structure prototyping, thermodynamic stability analysis, modeling of chemically disordered materials, and calculating a variety of electronic, thermal, and mechanical properties.	Curtarolo Materials Lab research group Center for Autonomous Materials Design, Materials Science Duke University	2013	yes (ML)
5	BIOVIA www.3ds.com/products/biovia www.technia.com/en/laboratory-management/software/biovia (both accessed on 27 May 2025)	Biovia represents a comprehensive scientific and management collaborative platform that helps organizations develop new materials and products. It delves into life sciences: chemistry (specifically material science, drug design, and formulation design) and biology, informatics (lab and data management, molecular modeling and simulation), quality control, and many more. It offers a space for virtual simulation of all materials, products, and processes that one might need in their lab, and it is backed-up by complementary platforms from the mother company. Dassault Systemes is science and technology based, offering product lifecycle management, digital mockup, 3D design, and virtual twins for any imagined product.	Dassault Systemes (French multinational software company) www.3ds.com/about www.3ds.com/virtual-twin (both accessed on 27 May 2025)	2001	yes (AI, ML)
6	AiiDA www.aiida.net/ (accessed on 27 May 2025)	AiiDA (automated workflows for computational science) is an open-source (MIT license) Phyton platform for automation, management, persisting, sharing, and reproducing data in computational science. It works with user-tailored workflows that can be shared and expended between collaborators.	AiiDA is a joint effort of the Materials Software and Data group (part of the LMS laboratory at Paul Scherrer Institute (PSI), Switzerland), the THEOS laboratory at EPFL (Switzerland), and Bosch RTC in Cambridge MA (USA). Its development is supported by NCCR MARVEL and a number of additional supporting partners and institutions. Data generated with AiiDA are disseminated through www.materialscloud.org. In addition to the AiiDA development team, there are numerous external code contributors as well as plugin developers who help build the Aiida ecosystem.	2019	yes
7	ThermoCalc https://thermocalc.com/ (accessed on 24 July 2025)	Thermo-Calc Software develops computational tools used to predict and understand materials properties, allowing you to generate computational materials data without costly, time-consuming experiments or estimations based on the limited data available. Thermo-Calc can be used to fill the gaps in material property data and make predictions of material behavior throughout the materials life cycle. With Thermo-Calc, you can make better decisions about your products and improve your material processing conditions with accurate, reliable materials data.	Thermo-Calc global company Founded at the Royal Institute of Technology (KTH), Sweden	1997 (roots in 1970s)	yes (AI, ML)
8	Gaussian https://gaussian.com/ (accessed on 24 July 2025)	Gaussian is a computational chemistry software that performs quantum mechanical calculations (especially focused on electronic structure) used in materials discovery.	Developed initially by a research group led by John Pople at Carnegie Mellon University in 1970, since 1987 has been licensed by Gaussian, Inc.	2017 (Gaussian 16) Roots in 1970	no
	MateriApps https://ma.issp.u-tokyo.ac.jp/en/ (accessed on 24 July 2025)	MateriApps represents a platform including several software packages for material science simulations. At the moment, it integrates 330 apps.	Started by a group of researchers at the University of Tokyo, but now it is a collaborative effort, including researchers from different institutions.	2013	some apps yes
9	nanoHUB https://nanohub.org chipsHUB https://chipshub.org (accessed on 25 May 2025)	nanoHUB is an open and free online platform for computational education, research, and collaboration in nanotechnology, materials science, and related fields. It provides modeling and simulation tools and educational materials to the world to have an impact on education and research. At its core, its hub is a website built with many familiar open-source packages—the Linux operating system, an Apache web server, a MySQL database, PHP web scripting, and the Joomla content management system. The HUBzero software builds upon that infrastructure to create an environment in which researchers, educators, and students can access simulation tools and share information. Chipshub is a central place with access to a variety of expert-level tools and apps that can be used in the chip design process.	https://nanohub.org includes two initiatives led by the Network for Computational Nanotechnology at Purdue University, USA. International partners: companies, research centers, organizations	2002	yes
Chemical Databases					
1	ICSD https://icsd.fiz-karlsruhe.de/index.xhtml (accessed on 24 July 2025)	ICSD is the world’s largest database (db) for completely identified inorganic crystal structures. ICSD contains more than 290,000 crystal structures of inorganic substances published since 1913. ICSD data are comprehensive, curated, and provide the perfect basis for finding answers on questions in materials research.	Initiated in 1977 at the University of Bonn and completely taken over by the FIZ Karlsruhe–Leibniz Institute for Information Infrastructure (not-for-profit company) in 2017.	1977	no (the db) yes (separate analysis software)
2	Spider-silkome https://spider-silkome.org (accessed on 26 May 2025)	Spider-silkome is a comprehensive database with information on spider silk, a very attractive protein-based biomaterial, considered one of the toughest natural materials. The database includes genes coding for the silk proteins and physical properties of the silk, structure, morphology, thermal properties, mechanical properties, and wet properties. The database does not use AI for its core; it is actually a resource for training AI to design new spider-silk proteins with tailored properties.	This database was created as part of the ImPACT Suzuki Project (Japan), a collaborative research project on structural proteins conducted in 2014–2018.	2014	no (but it is used with AI)
3	Reaxys http://www.elsevier.com/products/reaxys ! not free (accessed on 24 July 2025)	Reaxys is the largest chemical database, Reaxys combines over a billion chemistry data points with AI to support innovation in drug discovery, chemical R&D, and academia. Chemists can quickly access relevant patent, substance, and bioactivity insights, and an award-winning retrosynthesis tool.	Elsevier BV	2009 Roots in 1771 (Beilstein database)	yes
4	CAS SciFinder-n www.cas.org/solutions/cas-scifinder-discovery-platform/cas-scifinder ! not free (subscription based, some institutions, like universities or companies, provide free access to their students or employees) (accessed on 24 July 2025)	CAS SciFinder-n is a chemical and scientific database, the latest scientific information solution from CAS, a division of the American Chemical Society. In addition to the reference, substance, reaction, and supplier content found in CAS SciFinder^®^, CAS SciFinder-n includes relevance-ranked results, step-by-step procedures and protocols, citation mapping, biosequence searching, retrosynthetic analysis, patent landscape mapping, touch-screen enabled structure drawing, and much more—all accessible in a simple, easy-to-use interface. It is connected to other platforms/services from CAS, thus being a part of an ecosystem for drug and material discovery.	The Chemical Abstracts Service (CAS) is a division of the American Chemical Society (ACS)—an NGO responsible for curating chemical information from various scientific sources. SciFinder is a research tool, produced by CAS, that allows scientists to search the CAS databases.	1995 (software version) 2008 (web-based platform)	yes
5	ChEMBL www.ebi.ac.uk/chembl/ and ChEBI www.ebi.ac.uk/chebi/beta/ (both accessed on 26 May 2025)	Chemogenomic EMBL database (ChEMBL) is a manually curated database of bioactive molecules with drug-like properties. It brings together chemical, bioactivity, and genomic data to aid the translation of genomic information into effective new drugs. The database is not an AI-based system, but researchers use it in conjunction with AI and ML in drug discovery. and Chemical Entities of Biological Interest (ChEBI) is a freely available database of molecular entities focused on “small” chemical compounds relevant to biology and is widely used by other bioinformatics resources.	EMBL-EBI (the European Molecular Biology Laboratory’s European Bioinformatics Institute) is one of the six sites of EMBL, an intergovernmental research organization funded by over 20 member states and prospective and associate member states, located on the Wellcome Genome Campus near Cambridge, UK. It is a non-profit, intergovernmental organization. It integrates a number of databases: protein, genome-wide association studies, chemical, computational models, and so on. www.ebi.ac.uk/services/data-resources-and-tools/ (accessed on 26 May 2025)	EMBL 1974 EMBL-EBI 1980 (1992–1994 relocated to UK) ChEMBL 2009 ChEBI 2014	ChEMBL no (used with AI) and ChEBI yes
6	PubChem https://pubchem.ncbi.nlm.nih.gov/ (accessed on 26 May 2025)	PubChem is a public, open-chemistry database that contains information on chemical molecules, their properties, and biological activities. It actually contains three databases in one: Compounds, 111 million entries—contains pure and characterized chemical compounds; Substances, 293 million entries, contains mixtures, extracts, complexes, and uncharacterized substances. BioAssay, bioactivity results, and high-throughput screening programs with several million values.	The system is maintained by the National Center for Biotechnology Information (NCBI), a component of the National Library of Medicine, which is part of the United States National Institutes of Health (NIH), so it belongs to a governmental agency.	2004	no (it is used to train AI)
References [22,25,26,27,28,29,30,31,32,33,34,35,36,37,38,39,40,41,42,43,44,45]					

! indicates not free.

**Table 4:** Tissue engineering technologies.

No.	Technology	Operation	TE-Associated Applications	References
I. Technologies used to obtain Cell Sheets, Organoids, Tissues, and Organs (+Cells)				
1	Stem cell technology	Obtaining cells capable of differentiating into the types of adult cells necessary for the construction of the desired tissue: pluripotent stem cells—capable of giving rise to the 3 germ layers, in other words able to generate by differentiation any type of adult cell in the body (embryonic cells and induced pluripotent cells (iPSCs)); multipotent stem cells—generate a single germ layer (fetal cells, cells from the 3 germ layers, cells from the placenta or cells from certain organs, such as bone marrow—mesenchymal cells); oligopotent stem cells—generate a maximum of two cell subtypes (from the blood/marrow: hematopoietic, from the cornea: corneal and conjunctival); unipotent stem cells—they renew themselves and generate a single cell subtype (from organs: muscles, lung—type II pneumocytes from the alveoli generate type I pneumocytes, and so on).	➢ Cardiac tissue ➢ Spinal injuries ➢ Diabetes treatment (pancreatic tissue)	[70,76,80,81,82]
2	Cell sheet engineering	It is an advanced scaffold-free tissue engineering technique, developed in 1990, which involves cultivating cells to confluence on a surface covered with a temperature-responsive polymer such as poly(N-isopropylacrylamide) (PIPAAm), which allows cell harvesting without enzymatic treatment. For example, cells are cultivated at 37 °C and spontaneously detach when the temperature reaches 32 °C. This means a harvest which involves not only cells, but also their deposited ECM and intact cell-surface proteins. Moreover, it facilitates direct transplantation or an easier build of 3D constructs. It can be applied using different types of stem cells (embryonic, IPS, mesenchymal), but also adult cells (keratinocytes, fibroblasts, skeletal muscle cells and so on)	Treatment of various diseases/regeneration of various tissues and organs (studies at different levels): ➢ Preclinical in vitro: myocardial infarction, bone regeneration, & diabetic ulcer, ocular trauma, esophageal ulcer, skin defect, burn wound, periodontitis ➢ Preclinical in vivo: myocardial infarction, pancreatic fistula, liver failure, wound ulcer, ischemic limb, stroke & brain damage, esophageal ulcer, periodontitis ➢ Clinical: myocardial infarction, ocular trauma, esophageal ulcer, periodontitis	[70,83,84]
3	Organoid cultures	In vitro obtaining of mini-organs, using stem cells (embryonic or adult)/tissue fragments ± biodegradable matrices. Organoids mimic very well the 3D structure and functions of the organs they represent, except for nerve involvement. The vascular component is sometimes included (by 3D bioprinting), sometimes not. However, the interactions between the different cell types existing in the organ, the capacity for self-assembly and regeneration are faithfully reproduced, unlike the case of spheroids, which essentially represent more of a 3D culture, an aggregate, consisting of a single cell type (with applicability mainly in the study of oncology or stem cells—drug screening and cellular behavior). Usually, matrices are used to obtain organoids for architectural guidance, but there are exceptions. Organoids always exhibit polarity (organ-specific organization), unlike spheroids (which may only exhibit differentiation of concentric cell layers).	➢ Study of organ development ➢ Study of the functioning of organs ➢ mimicking in vitro diseases in a faithful manner (in vivo-like complexity, in vivo-like architecture & function) ➢ Immuno-oncology ➢ Drug discovery, development & testing: hit-to-lead optimization ➢ Personalized medicine in combination with CRISPR (patient-specific) genetic engineering technology ➢ Organoids obtained for: heart, brain (e.g., www.pascalab.org/research), lung, kidneys, intestines, liver, pancreas, prostate, thyroid gland, skin, retina	[70,85,86,87,88]
II. Technologies used to obtain Organoids, Tissues, Organs, and Scaffolds (±Cells)				
1	Bioprinting 3D/4D	Layer-by-layer deposition of bio-inks containing cells and biomaterials (for the purpose of obtaining organoids, tissue, or organs) or only biomaterials (when only matrices are manufactured), obtaining structures with well-defined microarchitecture and ensuring, in the case of bioprinting, a cellular viability of 80–90%. In 4D bioprinting, biomaterials that respond to stimuli are used, thus transforming the product into one that changes over time (hence the fourth dimension).	➢ Skin grafts ➢ Cartilage ➢ Vascularized tissues ➢ Personalized medicine	[70,87,89,90,91]
2	Cell patterning by laser-assisted bioprinting (LAB)	Bioinks are the central pieces of 3D, 4D, or successive laser-assisted bioprinting. A bioink is a combination of cells encapsulated in a biomaterial or in a combination of biomaterials in a hydrogel form. There are two kinds of bioinks currently used in bioprinting: (1) scaffold-based bioinks, consisting of cells and a scaffold (hydrogel/microcarriers/decellularized matrix), and (2) scaffold-free bioinks (no biomaterials are used as cell carriers, only cell aggregates are printed directly). Laser-assisted bioprinting (LAB) makes possible cell or liquid material printing at cell or picolitre resolution, which means one can fabricate artificial cell niches without affecting cellular viability or material characteristics. The technique is based on laser-induced forward transfer (LIFT), which is a method invented for high-resolution patterning of metals for computer chip manufacturing. It can print at high cell density (~10^8^ cells/mL) and high viscosity (10–100 μm) without transmitting mechanical stress to the cells, and it can print cell or cell aggregates per droplet with high accuracy. It is a sought-after technique due to its automation capacity, reproducibility, and high throughput.	➢ Tissue-like structures that have the physiological functionality of their native counterparts	[92,93]
3	Self-assembly	Tissue engineering by self-assembly (TESA) relies on the ability of mesenchymal cells (multipotent stromal cells) and of dermal fibroblasts to produce and assemble their own extracellular matrix upon ascorbic acid and serum supplementation, leading to manipulatable cell sheets and suturable constructs devoid of exogenous biomaterials for clinical and fundamental applications, using only patients’ cells. The obtained scaffolds have physiological strength and are not recognized as foreign in vivo. Fibroblasts can deposit enough ECM within a few days to create a three-dimensional (3D) stromal sheet. The stromal sheets can then be stacked or rolled to create a 3D organ substitute. In the process of tissue reconstruction, cells are extracted from a patient’s biopsy, and the stroma and the epithelium are enzymatically separated using thermolysin. Mesenchymal cells are extracted from the stroma using collagenase, while epithelial cells are extracted from the epithelium using trypsin. Despite the advantages, it is a time-consuming and costly procedure. Improvements in protocol have reduced both time and cost by increasing the rate of ECM formation. They have also increased stromal thickness and improved mechanical properties: mechanical stimulation, cell cultures in dynamic conditions, fiber alignment within engineered tissues (micro-structured surfaces—surface topography), enzymatic reactions and chemical stimulation, biological stimulation, reseeding self-assembly technique, improvement of blood vessel production, organ-specific stroma, and tissue engineering with serum-free medium (to avoid fibrosis). The introduction of vascularization strategies in vitro permits the formation of capillary-like networks within reconstructed tissues. These optimization strategies enable the large-scale production of inexpensive native-like substitutes using the self-assembly technique. These scaffolds have high porosity and high cell viability, but poor control over fiber dimension. Scaffolds made of self-assembling peptides: Instead of using synthetic polymers or natural decellularized matrices, self-assembling peptides (sa-peptides) are peptide monomers that spontaneously aggregate into sheets due to the arrangement of ionic hydrophobic and hydrophilic amino acids. These sheet structures then assemble further to create larger matrix constructs. Sa-peptides have similar cellular ingrowth characteristics to other matrices used for tissue engineering (PLLA: poly-L-lactic acid, PLGA: poly(lactic-co-glycolic acid), PCLA: poly(ε-caprolactone-co-L-lactide), collagen and Matrigel), but they offer the benefit of having no detectable immune response and their degradation products are digestible amino acid monomers that can be reused by the body. Polypeptide sequences larger than 35 amino acids stretch the limits of clinical economic viability. Transgenic organisms appear to be the best option for large-scale synthesis of peptides for use in self-assembling peptide scaffolds. Although sa-peptides are amino acid-based, the sequences used to create self-assembling scaffolds are not biologically relevant signaling sequences promoting cell binding and proliferation. That being said, because they are peptide-based, it is especially easy to integrate bioactive sequences into the sheet structures, which can increase cell proliferation, differentiation, and migration into these hybrid scaffolds compared to those without the integrated bioactive sequences.	➢ Tissue-engineered skin, blood vessel (e.g., used as arteriovenous shunt), heart valve, cornea, adipose tissue, urologic tissue, vaginal mucosa	[94,95,96,97,98]
4	Layer-by-layer (self)-assembly and biomimetic LbL self-assembly	Layer-by-layer (LbL) assembly is a multilayer self-assembly technique that allows for the fabrication of multilayer coatings with controlled architectures and compositions, serving various biomedical applications. It basically builds solid films at molecular level. The LbL assembly process includes the sequential adsorption of complementary molecules on a substrate surface, promoted by electrostatic and/or non-electrostatic interactions. Steps of washing and drying are usually introduced between the steps of deposition to avoid contamination of the next solution and to elute the loose molecules and stabilize them in the formed layers. The composition, thickness, structure, and topography of the assembly and of each nanofilm can be controlled by tunning the solution properties (concentration, ionic strength, pH) and process parameters (temperature, time, drying conditions). Various building blocks used for LbL assembly include, but are not limited to, natural polymers, synthetic polymers, peptides, clays, metal oxides, polymer gels, and complexes of such materials. There are a variety of deposition technologies: dipping, centrifugation, roll-to-roll, calculated saturation, creaming, immobilization, atomization, spinning, spraying, magnetic assembly, high gravity, electrodeposition, electrocoupling, filtration, fluidics, and fluidized beds, which can be classified into five main categories: (i) immersion; (ii) spin; (iii) spray; (iv) electromagnetic-driven; and (v) fluidic assembly. Biomimetic LbL self-assembly: Self-assembly is the autonomous organization of components from an initial state into final pattern or structure without external intervention. Living organisms, in particular the developing embryo, are quintessential self-organizing systems. Histogenesis and organogenesis are examples of self-assembly processes, in which, through cell–cell and cell–extracellular matrix (ECM) interactions, the developing organism and its parts gradually acquire their final shape. It is through cellular self-assembly that the morphologically featureless zygote evolves into the fully developed organism with its numerous structures of widely varied shapes and forms. Although the sequence of morphogenetic processes that underlies early development is under strict genetic control, additional physical mechanisms are mobilized to move mass and make shapes. In turn, the changes brought about by physical processes (e.g., diffusion, changes in shape, molecular and ion concentration) provide feedback to gene expression. The interplay of genetic and physical processes is the hallmark of embryonic development. The morphogenetic mechanisms that are utilized in the bio-fabrication technology are: - Cell sorting: a self-assembly process providing a common mechanism to establish cellular compartments and boundaries between distinct tissues; - Tissue fusion: a self-assembly process in which two or more distinct cell populations make contact and coalesce. Apparent tissue liquidity: during sorting and fusion, the cellular system evolves in time from an initial state to a structurally and energetically more stable final state. As sorting and fusion strikingly resemble, respectively, the phase separation and the coalescence processes observed in liquids, it was proposed that adhesive and motile cell populations have apparent liquid-like properties. Based on this analogy, cell sorting and tissue fusion should be driven by apparent surface and interfacial tension. An apparent tissue surface tension has been measured using methods applicable to liquids for a wide variety of naturally occurring tissues and tissue cell aggregates. Self-assembly approaches may have major impact on the development of the intermediate vasculature, which is the “missing link” for establishing a perfused vascular tree. A method based on the modular assembly of endothelialized microtissues to form macrotissues has been reported, where modular tissue-engineered constructs were assembled from sub-millimeter-sized cylindrical modules of collagen or gelatin seeded with cells and endothelialized at the surfaces. EC-covered modules then randomly self-assembled into a modular construct with interstitial spaces that enabled perfusion with medium or whole blood.	LbL self-assembly: ➢ Nanofilms directing cellular phenotypes ➢ Nanofilms encapsulating cells and tissues ➢ Nano-coatings for cell co-culture ➢ 3D scaffolds for TE with/without cells ➢ Biosensors ➢ Bioreactors ➢ Drug delivery ➢ Medical implants Biomimetic LbL self-assembly: ➢ Vascular networks ➢ Cardiovascular scaffold-free grafts	[72,99]
III. Technologies used to obtain and design Scaffolds (-cells)				
III. 1. Conventional technologies				
1	Decellularization	Removal of cells from donor tissues or organs, leaving behind the ECM, which retains the architecture and mechanical properties of the native tissue, being ideal for recellularization with the patient’s cells	➢ Heart valve ➢ lungs ➢ Liver ➢ Organ transplantation without immune rejection ➢ Personalized medicine	[70]
2	Electrospinning	Solution-based technology => fibrous scaffolds Obtaining micro- or nano-fibrous matrices, which mimic the ECM structure, generating a conductive environment for tissue growth and regeneration. The shape of the matrix is well controlled, and this promotes cell adhesion, proliferation, and differentiation. The electrospinning process involves inducing filaments from a polymeric solution into high-voltage electric current. A high-voltage system connected to a syringe is used, to which a metal needle, a pressure pump, and a grounded, stationary, or rotating manifold are attached. The liquid solution, pumped into the syringe, flows to a drop suspended at the tip of the needle due to the surface tension. An electric current is applied to the needle, which electrifies the solution, in which electrostatic repulsion develops due to the charges of the same sign of the polymer molecules. These repulsive forces overcome the surface tension, so the droplet elongates forming the “Taylor cone” (as the applied current is greater), which is then extruded in the form of a jet of fibers, which are deposited on the collector, while the solvent evaporates. Thus, a network of non-woven fibers is generated at the collector.	➢ Fabrication of fibrous scaffolds ➢ Matrices with low porosity, small pore size, and large volume/area ratio are obtained, and the cells are spread either on the surface of a single fiber of the matrix, or cross several fibers/pores, creating either an aligned or a randomized layout ➢ Wound healing ➢ Nerve regeneration ➢ Vascular grafts (artificial blood vessels)	[70,98,100,101]
3	Layer-by-layer (LbL) electrospinning	Solution-based technology => fibrous scaffolds Layer-by-layer electrospinning uses the principle of classical electrospinning but raises it to a 3D scale by alternating layers of fibers and generating a multilayer structure. Layer-by-layer or multilayer structures are reliable because electrostatic interactions are formed between the layers and, in addition, solve many of the shortcomings of electrospun mono-layers. In the case of wound healing dressings, a sustained release of the drug can be ensured by loading it into the central/intermediate layer or the upper layer (as such, or into functionalized nano-carriers). This eliminates contaminants from the wound area (bacteria, viruses, toxins, metal ions) and avoids immediate and uncontrolled release from the layer in contact with the skin. Moreover, the layered configuration mimics the skin much better at the tissue level, but also the extracellular matrix at the cellular level. Each layer can be customized in terms of fiber diameter, composition, and porosity. At the same time, the topography (surface characteristics) can be adjusted by varying the composition, pH, extrusion time, and ionic strength of the polyelectrolyte. All these customizations facilitate the obtaining of superior drug-delivery systems, as well as superior matrices of artificial tissue. In tissue engineering, a different biomaterial can be used for each layer, with distinct properties and signaling molecules incorporated to serve different functions, and the 3D configuration promotes differentiated cell expression while reducing cell scattering.	➢ Fabrication of LbL (multilayered) fibrous scaffolds ➢ Wound healing ➢ Advanced drug-delivery systems/patches ➢ Artificial skin	[101]
4	Crosslinking	Crosslinking is a method of improving the mechanical properties and maintaining the morphology of a matrix and is generally applied after electrospinning, i.e., fibrous matrices. Basically, the polymer mesh crosslinks additionally, fixing the structure of the matrix. This also avoids the collapse of the pores and the swelling of the matrix in contact with water. The methods are: physical—by radiation (gamma or UV), dehydrothermal treatment (increasing the temperature under vacuum to eliminate free water and water bound by hydrogen bonds); and chemical—with synthetic (glutaraldehyde) or natural (genipin, citric acid) agents. However, crosslinking also represents a range of methods of direct gelling of biopolymer matrices (used in bioinks or to obtain dressings). The methods are: physical—the above + crystallization, stereo-complexing, heating-cooling, hydrogen interactions and/or ionic interactions (polymeric combinations), addition of a hydrophobic agent, and maturation by heating; chemical—as above; and enzymatic—the use of transglutaminase for cross-linking peptide hydrogels or horseradish peroxidase for cross-linking polysaccharide hydrogels.	➢ Mechanically strengthened and water-resistant fibrous scaffolds ➢ Electrospun fibers of zein (plant protein) ➢ Bone tissue engineering	[98,102,103]
5	Freeze-drying (freeze-drying) and emulsion freeze-drying	Solution-based technology => foam-like scaffolds Freeze-drying The technique of manufacturing 3D matrices by removing water from a biological (natural) or synthetic material but maintaining its structural integrity (complex geometry) and uniform pore morphology. It involves four steps: pretreatment (improving stability with bulking and buffering agents, collapse temperature modifiers, tonicity modifiers, anti-microbial agents, surfactants, solvents, and complexing agents), freezing (turning water content into ice, for example using liquid nitrogen), primary drying (sublimation—95% of the water removed; the polymer creates an arranged network within the interstitial spaces), and secondary drying (desorption/evaporation: water molecules bound to the material are removed by further reducing the pressure and slightly increasing the temperature). Freeze-drying emulsion: It uses a solvated polymer emulsified in a non-miscible liquid. The homogeneous mixture is poured into a mold and freeze-dried. The higher the amount of the emulsifier, the higher the porosity of the scaffold. Varying parameters are the type of polymer and the quantity of the emulsifier: the higher the amount, the higher the porosity of the final scaffold. Matrices produced using this method tend to have good connectivity of the internal pores.	➢ Fabrication of foam-like scaffolds and other biomaterials (e.g., polysaccharide scaffolds made of collagen or chitosan). The scaffolds present high porosity (>90%) and large pore size (20–400 μm), characteristics that enhance the formation of 3D large cell aggregates among the fibers/pores ➢ Scaffolds with organ-specific geometries ➢ Natural biomaterial patient-specific geometries that preserve the bioactivity and the regeneration potential of the scaffold	[70,95,100,104,105,106]
6	Phase separation methods: thermally induced phase separation (TIPS) and diffusion induced phase separation (DIPS)	Solution-based technology => foam-like scaffolds Phase separation, when applied in dual or multiple consecutive steps, is very promising in the fabrication of 3D nanofibrous scaffolds with uniform pore structures, compared to electrospinning, and it can be used together with other fabrication techniques, such as solid free-form (SFF), also known as rapid prototyping. Thermally induced phase separation (TIPS) is a cost-efficient and relatively easy to apply technique for scaffold fabrication in TE, which has tunable parameters (polymeric system and concentration, solvent and non-solvent system and the ratio between them, cooling temperature and rate) that allow for a controllable architecture of the resulting membrane (pore dimensions, pore arrangement: fibrillar—nano/micro-fibrous, isotropic—random-pore, anisotropic—oriented/aligned-pore or microtubular, bilayer, biphasic structures, lamellar platelets, lamellar stacks, axialites, spherulites, or a mixture of these structures). The scaffold maintains its biocompatibility because all the solvents used in the process can be completely removed (using freeze-drying at the end). The method involves reaching the demixing temperature (a thermodynamically unstable condition) in a homogenous system made of polymer, solvent, filler, drug, and other components. Generally, the polymer solution is brought from a higher to a lower temperature. This results in a separation of two phases: a polymer-rich (with a high polymer concentration) and a polymer-lean phase (with a low polymer concentration). The polymer-rich phase is then solidified, forming the skeleton of a porous scaffold (made of a thermoplastic crystalline polymer), and the polymer-lean phase generates pores by solvent removal. The scaffolds fabricated this way have a porosity over 95% and pore diameters from ~1 to 100 μm. Being a low-temperature method, it allows the addition of bioactive molecules into the scaffold. Compared to TIPS, in the DIPS method, the first step is the molding of a thin layer of the polymer solution onto a solid support (i.e., tube, fiber), followed by immersion in a nonsolvent bath to induce the demixing. Both TIPS and DIPS usually involve a mold that the foams are poured into to form the scaffolds, which is removed at the end of the process. The scaffold is washed in distilled water and then dried in a desiccator to remove any trace of solvent. Also, both techniques can and will usually be combined with other methods, such as electrospinning, porogen leaching, 3D printing, and so on. For example, DIPS 3D printing inks consist of polymers, crosslinkers, water, and urea (reversible hydrogen-bonding dissociate) in a homogeneous phase solution. Basically, DIPS 3D printing inks are developed by adding a hydrogen bond dissociate (HBD) into hydrogen-bonded cross-linked hydrogels. If extruded into water and subsequent diffusion of HBD, these inks cure rapidly due to the regeneration of hydrogen bonds, so their self-healing property is activated. The introduction or diffusion of urea acts as a switch, allowing for ink phase separation and rapid curing in water while maintaining exceptional form fidelity. DIPS-printed scaffolds are also self-repairing, and therefore they can be recycled and reprinted, causing very little waste of materials.	➢ Applications for regeneration of cartilage, bone, osteochondral, dermal, cardiovascular, neural tissues, and so on ➢ Chemical-induced topological modification of surfaces used for better spreading of human fibroblasts ➢ DIPS 3D printing inks: scaffolds for dynamic tissue reconstruction, wearable devices, soft robots	[90,100,107,108,109]
7	Porogen leaching (also named solvent casting and particulate leaching—SC/PL)	Porogen leaching is the process of introducing pores into a polymer scaffold by utilizing a sacrificial substance, a porogen (inorganic or organic), like salt or sugar, which is chemically or physically incompatible with the scaffold (the porogen is dissolved away at the end of the process). Examples of inorganic porogens are salts (including sodium chloride, sodium hydrogen carbonate, ammonium carbonate, calcium carbonate, ammonium bicarbonate, and ammonium chloride), silica, and supercritical carbon dioxide. Examples of organic porogens are starch, polystyrene, poly-ethylene glycol (PEG), polyvinyl pyrrolidone (PVP), polyvinyl alcohol (PVA), and polymethacrylate (PMA). The homogenous solution of salt/sugar particles of a certain size is used to dissolve the polymer solution. The solvent is then evaporated, leaving a matrix containing the salt/sugar particles. This matrix is then soaked in water or a specific solvent for the particles, and so they are leached away, leaving a structure with high porosity (some say 50–90%, others >90%). The technique is easy and low cost; however, it is suitable only for thin membranes, and because of this, it is a time-consuming method. By modifying the size, geometry and concentration of the porogen in the polymer solution, one can adjust the size, geometry and density of pores.	➢ Large, three-dimensional, porous scaffolds for metabolically active tissues (they allow for rapid diffusion of nutrients, good waste removal, large surface area/volume for cell attachment and proliferation) ➢ Thin membranes	[90,95,107,110]
8	Melt molding (or simply, molding)	Melt molding is a method that shares similarities with TIPS and SC/PL because it uses temperature and porogens to adjust the 3D morphology of a thermoplastic polymer scaffold. In this process, the polymer combined with the porogen is heated above the glass transition temperature or melting point of the mixture, at high pressure, becoming liquid. The high temperature used impairs the addition of bioactive molecules. After this step, the mix is poured into a mold and allowed to cool in order to solidify. Unlike in TIPS, the polymer used is solid, granulated, or powdered, and unlike in SC, there is no need for polymer solvents, so there are no residual toxic solvents in the matrix. However, similarly to PL, the porogen is leached out using a suitable solvent for it (usually non-toxic), and the scaffold becomes porous. The method is convenient and rapid, producing scaffolds with various shapes and sizes, which makes it adequate for industrial applications and scale-up of laboratory prototypes. However, it can create non-porous layers on the surface of the scaffold, and it can leave particles of the porogen behind.	➢ Scaffolds with various shapes and sizes ➢ Industrial applications	[95,111]
9	Gas-foaming	This method does not use solvents, so it allows the incorporation of sensitive bioactive molecules into the obtained scaffolds, preserving most of their bioactivity. Firstly, a polymer is compressed at high temperature to become a solid disc. After that, it is placed in a high-pressure CO2 or N2 chamber, so that the inert gas infiltrates and saturates the mold with bubbles, creating the pores. Generally, the resulted scaffolds look like sponges with a pore size of 30 to 700 μm and a porosity up to 85%.	➢ Porous scaffolds with good cell adherence for 3D tissues	[90,95]
10	Lithography, microlithography nanolithography photolithography and advanced methods of lithography	Lithography is a micro- and nano-fabrication technique that enables the formation of precise and complicated two-dimensional or three-dimensional structures at extremely small scales. Microlithography includes any manufacturing process that generates thin films, with a microscopic pattern (minutely patterned thin films), with dimensions of a maximum of 10 micrometers/shape. Nanolithography is the same technique, but the level at which the model is generated is below 100 nm/shape. Photolithography (optical lithography) is a subclass of microlithography, a process by which thin films with a certain pattern (patterned thin films) are generated. It is a technique taken from the integrated circuits (microchips) industry. It involves the use of light radiation (UV, deep UV, extreme UV, X-ray) for the transfer of a pattern on a substrate. A photosensitive (photoresist) material is applied over the substrate. Over the photoresist, a photomask (an opaque material with transparent areas) is applied, which contains the pattern. The photomask is illuminated, exposing the photoresist in certain areas, which will undergo a chemical transformation that will make them either soluble (direct version) or insoluble (indirect version) compared to a developer. After development, the pattern is transferred to the substrate (by etching, chemical vapor deposition, or ion implantation). Also, after development, a thin layer of bioactive molecules is sometimes deposited, such as peptides, proteins, or bioactive polymers. Substrates in tissue engineering can be photo-linkable biodegradable polymers. Dry etching means unmasking the pattern in a material by removing the excess substance through exposure to a bombardment of ions (plasma of reactive gases, such as oxygen, chlorine, or fluorocarbons). Chemical vapor deposition (CVD) is a method that uses deposition under vacuum to create high-quality, performant, solid materials. The substrate, in reaction with volatile compounds, generates the desired deposit. Ion implantation is a low-temperature process by which ions of one element are accelerated into a solid target, thereby changing the target’s physical, chemical, or electrical properties. Advanced methods of lithography: soft lithography (working on “soft-matter”: organic materials, polymers, complex biochemicals), nanoimprint lithography, nano-molding PRINT particles (particles fabricated through to a top-down scalable and good manufacturing practice (GMP)-amenable nanofabrication approach: “Particle Replication in Non-wetting Templates”, which combines lithography with the processes from the photographic film industry to make micro and nano particles with perfectly controlled size, shape, chemical composition, cargo, modulus, and surface properties, which is extremely useful in materials science and biomedicine).	➢ All kinds of micro- or nano-patterned scaffolds for tissue engineering ➢ Advanced drug-delivery systems (e.g., drug-loaded microneedles—fabricated using the advanced methods of lithography) ➢ Drug-loaded and imaging agents-loaded nanoparticles made of hydrogels, polymers or proteins (PRINT technique)—used in nanotheragnostics	[79,112,113,114,115]
11	Microcontact printing (μCP)	The most used soft lithography technique due to its simplicity, flexibility, and costs. Pour an elastomeric die over a microstructured base. In the direct version, the stem imprints a protein cell receptor, while in the indirect version, a secondary bioconjugate is used, which is functionalized with another protein (e.g., biotin–avidin). The technique activates geometric, topographical indices. Generally, μCP induces various cellular responses by stimulating cell surface receptors and activating biochemical pathways. In the case of obtaining myocardial constructs, this approach resembles both micro-topography (because cell alignment is done by constraining cells in the patterned area) and nano-topography (because cell alignment is achieved by the interaction of surface receptors).	➢ Modeling of laminin on soft polyacrylamide hydrogels light-cured on glass ➢ Cardiomyocyte-forming patterns that form myofibers ➢ Study platform for mechanical interactions that modulate cytoskeletal tension (e.g., between cardiomyocytes and ECM) ➢ Specific vascular differentiation (immobilized VEGF modeling on IV collagen, on a layer of chitosan, non-cellular adhesive) ➢ Obtaining multicomponent surfaces with multiple molecular species (using multiple bioinks on chemically modeled scent, micro-alignment, and modeling) ➢ Functionalization of multicomponent surfaces	[113]
12	Microfluidics/microfluidic patterning (μFLT)	Manipulation of fluids at the microscale, following their flow and surface tension, to mimic physiological conditions. In μFLP, microfluidic channels created by the stub are used to deliver fluids to selected areas of a substrate. Excess fluid and mold are removed from the surface, leaving behind molecules that are physio-absorbed according to the pattern.	➢ Microfluidic chips ➢ Organs-on-a-chip for testing drugs’ cytotoxicity (liver-on-a-chip) ➢ Angiogenesis study (vascularized tissues) ➢ Contractile cardiac organoids, obtained by preferential adhesion of newborn mouse cardiomyocytes to fibronectin deposited over hyaluronic acid.	[70,113]
13	Spin-coating	By the spin-coating method, thin films of equal thickness (less than 1 μm) can be obtained on flat surfaces. The liquid material is dripped onto the center of a substrate (a flat surface that is stationary or spinning at low speeds), then the substrate is rotated (with a spinner) at speeds of up to 10,000 rpm so that the liquid is scattered by centrifugal force. The solvent used is usually volatile and dries out, or the spinner simultaneously heats the film to evaporate the solvent. There is a transition point where the thinning of the film by flow is equal to that by evaporation, then the film immobilizes on the substrate and only thinning by evaporation occurs. The spinner is then turned off and the film is final. The films can thicken depending on the viscosity of the solution with which they work and depending on the number of layers that are applied. In photolithography, the photoresist layers (1 μm thick) are made at 20–80 rpm and 30–60 s. It is a technique with very high reproducibility, and it is simple and efficient in terms of time, but inefficient in terms of the use of the material (a lot is lost in the process, 95–98%). The thickness of the films obtained varies from tens of nanometers to a few micrometers, and their surfaces cannot exceed 30 cm because the homogeneity of the film is lost. The technology provides from the microelectronics industry. In the biomedical field, it is applied for the functionalization of surfaces, wound patches, substrates for cell cultures, and drug-delivery systems. The combination of spin-coating with LbL technology leads to the creation of customized multi-layered films. Spin-assisted LbL (SA-LbL) assembly or spin self-assembly or self-assembly has replaced the dip-coating technique (the adsorption method by which layers are generated in a few minutes, while spin-coating takes a few seconds). The adsorption of different materials on a substrate is facilitated by electrostatic interactions, Van der Waals forces, and hydrogen bonds. In general, polyelectrolytes with opposite charges are used in consecutive layers to facilitate the bonding of the layers, although the entanglement between the polymers plays a preponderant role. The thickness of the films obtained by spin-coating is usually less than 1μm, and in biomedical applications less than 100 nm (the products obtained are called nanosheets). In order to use these nanosheets independently (not just as coatings of a substrate), techniques have been developed to detach them from the substrate. Therefore, they can now be attached to other surfaces, and can be part of filter membranes, drug-delivery systems, matrices, or biological tissues. Nanosheets are biocompatible and resorbable membranes, with unique interfacial and mechanical properties, transparency, flexibility, high adhesiveness (<100 nm), and large surface area-to-aspect ratio (cm size—nm thickness), and are made of natural or synthetic polymers (e.g., PLA, PLGA).	➢ Monolayer and multilayer thin coatings ➢ Functionalized surfaces ➢ Cell culture substrates/platforms which can direct cell behavior, including spatial arrangement and differentiation (spin-coating + micropatterning techniques => topographic micro-motifs => regenerative applications: skin, heart tissue, etc.) ➢ Wound dressings (sheet-type, glue-type biomaterials for wound dressing and tissue regeneration, nano-adhesive plasters) ➢ Drug-delivery systems/patches ➢ Biosensing membranes, filtering membranes ➢ Biomimetic micropatterned films for LbL structures ➢ Freestanding (FS) nanosheets and membranes: bioactive and drug-delivery nanosheets (small molecule drugs, proteins, nucleic acids) ➢ Combination of spin-coated adhesive nanosheets with bioactive drug-delivery nanosheets for burns or surgical sites that are difficult to close, that need to be sealed to prevent infection, and where local delivery of drugs is needed to avoid their side effects ➢ Injectable magnetic nanosheets for cell transplantation, potentially useful in age-related macular degeneration (AMD), an ophthalmic disease that causes visual impairment	[116,117,118]
14	Dip-coating	Through the dip-coating method a substrate is firstly immersed into a melt or a solution of the coating material and secondly extracted using a controlled speed. This way, a thin layer of the coating material is deposited on the substrate. Polymers, ceramics and metals can be coated this way, and it is a cost-effective and simple method for which extraction speed and coating solution viscosity and concentration are tunable parameters.	➢ Biosensors ➢ Drug delivery ➢ Surface-modified scaffolds ➢ Thin films	[109,119]
15	Laser patterning	An important class of (bio)material processing methods is represented by laser techniques, which are extremely precise and make minimal disruptions. These techniques are commonly grouped into three categories: polymerization (use of a laser to induce cross-linking between biomaterial polymer chains), ablation (use of a laser to selectively remove part of the biomaterial by thermal, photo-physical, or photo-chemical effects), and activation (use of a laser to activate certain parts on the polymer chains for specific applications). Cell response is dependent on surface topography, and biomaterials’ (and implants) performance inside the body can be improved by selectively modifying their surface properties, for example, by depositing thin films of biomaterials one over another. To obtain thin coatings from biomaterials, one can use deposition techniques: pulsed laser deposition (PLD) or matrix-assisted pulsed laser evaporation (MAPLE). Compared to other methods, laser processing of biomaterials has reduced surface contamination and mechanical damage and the capability to produce three-dimensional components with complicated geometries by controlling the surface structuring. Surface modification or topography patterning is critical for the integration of TE substituents in the body, as it influences cell fate through chemical and physical signals, as presented earlier. An in vivo study outlined the strategic importance of modifying the topography of implants with lasers by showing a stronger bone-to-implant bond. Femtosecond (fs) lasers allow for the biological response tailoring of surface morphology by controlling the surface pattern geometry down to a 100 s-nm scale thanks to their unique characteristics (laser patterning).	➢ Bone prosthetics	[79,120,121]
16	Microfabrication techniques	They control the microarchitecture of the scaffold and provide a platform for studying cell–matrix interactions.	➢ Applications described above for each technology that works at micrometer scale	[79,113]
17	Nanofabrication techniques	They control the nanoarchitecture of the scaffold (topography down to the molecular level) and generate matrices with a thickness of <100 nm, generally fibrous (electrospinning, lithography, spin-coating).	➢ Applications described above for each technology that works at nanometer scale	[113]
III. 2. Rapid prototyping (solid free-form) technologies				
	Rapid prototyping (RP) or solid free-from fabrication (SFF) technologies can generate direct forms directly from computer-aided design (CAD) models of an object without other specific tooling or knowledge. The RP systems combine powder, liquid, and sheet materials to build the model using a layer-by-layer approach. They offer precise spatial control over polymer structure, and they allow for personalized, patient-specific scaffolds. The main RP techniques are 3D printing (3DP), fused deposition modeling (FDM), selective laser sintering (SLS), and stereolithography. Three-dimensional printing encompasses (uses) the other three technologies mentioned.			[90]
1	Stereolithography (SLA)	It is used to fabricate solid 3D objects by consecutively (layer-by-layer) printing a thin layer of ultraviolet (UV) curable material. At the end, the uncured resin is cleaned off, and the scaffold is post-cured under UV light. It overcomes the challenges related to wastage in subtractive fabrication methods, and it offers high resolution and uniformity in pore interconnectivity, but the feature size of a scaffold that can be fabricated is limited to the beam width of the laser, and it requires massive amounts of monomers and post-polymerization treatment to improve monomer conversion.	➢ Scaffold structures of sophisticated design at high resolution ➢ Different types of cellular machines for biosensing, environmental remediation, drug discovery, and energy harvesting	[90]
2	Selective laser sintering (SLS)	It uses a laser to sinter a powdered material in thin layers, according to a 3D model. Because it is using a laser, it can process polymers, metals, or ceramics. It provides user excellent control over the microstructures of the produced scaffold by adapting the process parameters. The drawbacks are that it uses a high temperature and an additional procedure is needed to remove the injected powder.	➢ Scaffolds using ultrahigh-molecular-weight polyethylene ➢ Bio-nanocomposite microspheres	[90]
3	Solvent-based extrusion free forming (SEF)	It offers precise control of scaffold structure at the micron level, and it can strictly follow the structure of the natural tissue and the mechanical characteristics of the scaffold	➢ Ceramic, metal, and metal/ceramic composite parts	[90]
4	Fused deposition modeling (FDM)	A solid polymer is cast into a hot extrusion nozzle to be melted and extruded on the surface of a 3D object using computer-controlled extrusion and deposition processes; the scaffold is made from multiple layers of adjacent microfilaments. It is a low-temperature deposition technique, but it has limitations in its application to biodegradable polymers.	➢ Nonwoven scaffolds ➢ New scaffold structures with controlled mechanical properties	[90]
5	3D printing	Three-dimensional printing is a method for prototype manufacturing using computer models. Powdered material is selectively fused by inkjet (the adhesive is also added), and the layers of the construct are layered one by one, creating a 3D complex object with high speed, low cost, and high accuracy.	➢ All kinds of scaffolds	[90]

**Table 5:** Roles and applications of polymer scaffolds [77].

Role	Applications	Examples
Space fillers	Fillers used in implants for urinary incontinence or vesicourethral reflux, plastic surgery, and reconstruction	porous scaffolds with alginate modified with arginine–glycine–aspartate (RGD) adhesion peptide glutaraldehyde crosslinked collagen matrices dextranomer and hyaluronic acid scaffolds PEG matrices with embedded bone morphogenetic protein 2 (BMP-2)
	Prevention of post-operative adhesion	
	Biological glue for soft tissues	
Bioactive molecule delivery vehicles	Stimulation of angiogenesis	
	Encapsulation of secretory cells	
	Drug-delivery systems	degradable gels made of poly(ethylene glycol)-poly-L-lactic acid (PEG-PLLA) PEG-PLLA temperature-responsive gels glutaraldehyde-crosslinked chitosan microspheres PEG bilayers encapsulation of cells for sustained and controlled release of enzymes or hormones in the human body (examples: pancreatic, hepatic, adrenal cortical cells, cells transfected to release certain enzymes or growth factors); polymers used for this purpose: ionic crosslinked alginate because the cells can be mixed with the polymer before gelling and crosslinking occurs under relatively mild conditions, PEG to coat the alginate microspheres containing cells or to directly encapsulate cells
Three-dimensional structures (scaffolding) of cell organization and signaling for obtaining tissues (almost all types of tissues from the human body) or for cell delivery	Cartilage tissue *	photo-crosslinked PEG dies chitosan treated by freeze-thawing hyaluronic acid alone or combined with alginate alginate combined with chondrocytes and injected at the target site or shaped into the required shape and implanted
	Bone tissue *	hyaluronic acid scaffolds seeded with bone marrow stromal cells, possibly preincubated with basic fibroblast growth factor (bFGF) alginate matrices modified with RGD or BMP-2
	Smooth muscle tissue	photo-crosslinked PEG hydrogels with proteins + growth factors + embedded enzyme degradation sites (used as scaffolds for smooth muscle cell production and proved more effective than plain PEG hydrogels; enzyme degradation sites aid in cell migration and in collagen production, compared to non-degradable gels)
	Striated muscle tissue	collagen scaffolds peptide-modified alginate scaffolds
	Liver tissue	
	Nervous tissue	alginate hydrogels as scaffolds for Schwann cells used as nerve grafts
	Lipid tissue	freeze–thaw collagen sponges seeded with preadipocytes
	Vascular tissue (blood vessels)	collagen scaffolds

* Note: In the cartilaginous and bone tissue examples, the scaffolds were used as cell delivery systems, and no effort was made to control the fate of the cells or their subsequent organization.

## Data Availability

All the generated databases (Excel and plain text files) mentioned in the bibliometric study (Chapter 2: Methodology), as well as the thesaurus file used to create the bibliometric maps with the VOSviewer software and Table S3 from the Supplementary Materials (Excel, filterable format) can be accessed publicly using the following link: https://drive.google.com/drive/folders/19LBQp6KECYJlAl8-GcRjCD3stQ-fLsUu (accessed on 15 April 2026). Further inquiries can be directed to the corresponding authors.

## Supplementary Materials

The following supporting information can be downloaded at: https://www.mdpi.com/article/10.3390/jfb17050214/s1 [48,49,157,158,159,160,161,162,163,164,165,166,167,168,169,170,171,172,173,174,175,176,177,178,179,180,181,182,183,184,185,186,187,188,189,190,191,192,193,194,195,196,197,198,199,200,201,202,203,204,205,206,207,208,209,210].
