# Supplementary material for: Biomaterials’ Role in Improving Patient Care from Drug Testing and Delivery to Theragnostics and Regenerative Medicine
Source: J Funct Biomater. 2026 May 1;17(5):214. doi: 10.3390/jfb17050214 (PMC13207391; doi:10.3390/jfb17050214)
Supplement: Supplementary file 1 [file jfb-17-00214-s001.zip › jfb-4209616-Supplementary Materials.pdf]

Supplementary materials

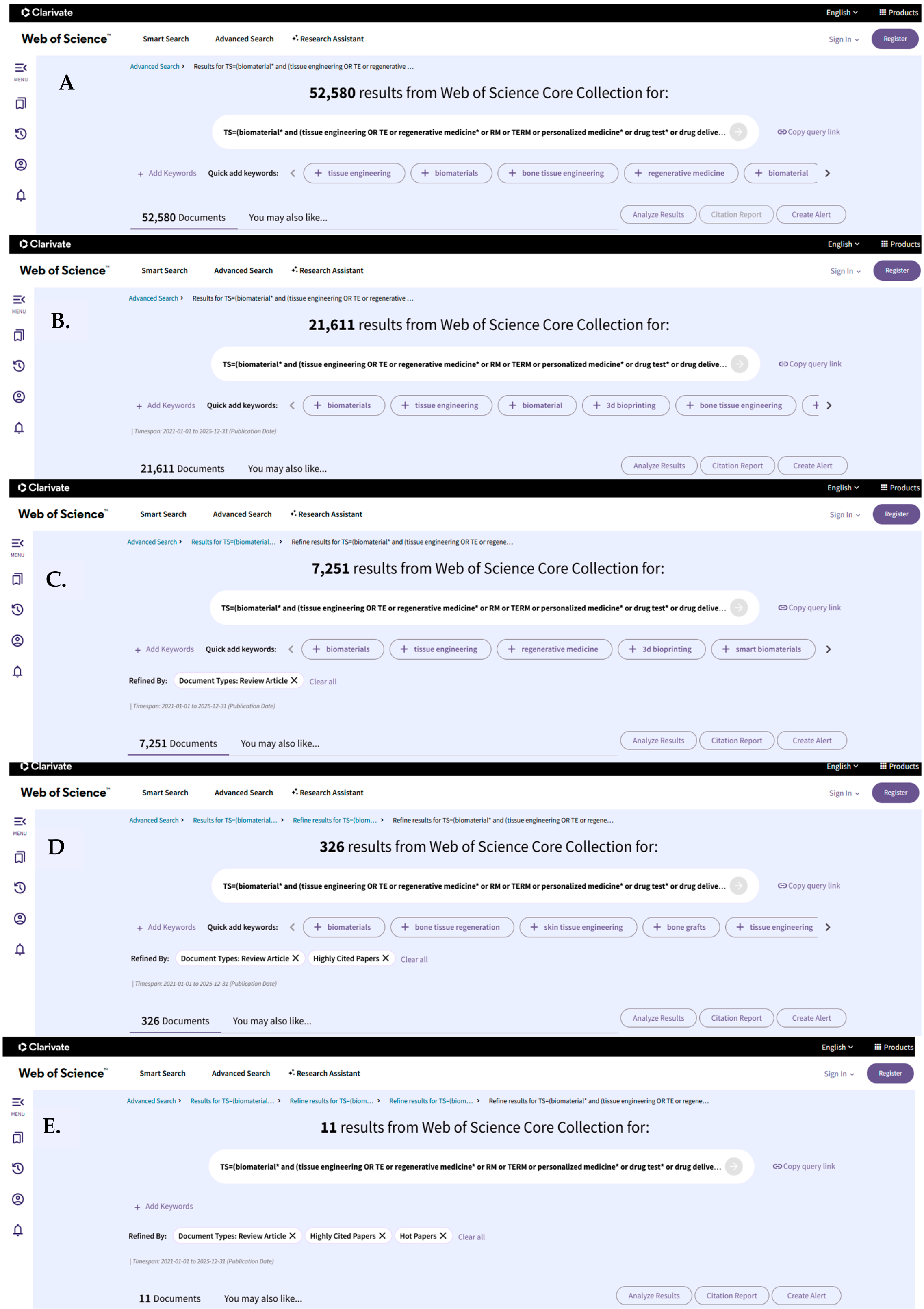

Figure S1. Print screens showing the number of generated records from WoS database, after advanced search and filtering. **A.** Database (Db) with 52 580 results, after query; **B.** Db with 21 611 results (2021 – 2025 filter); **C.** Db with 7 251 results (document type: review filter); D. Db with 326 results (highly cited papers filter); E. Db with 11 results (hot papers filter). Filters are connected by the "AND" operator, being applied consecutively.

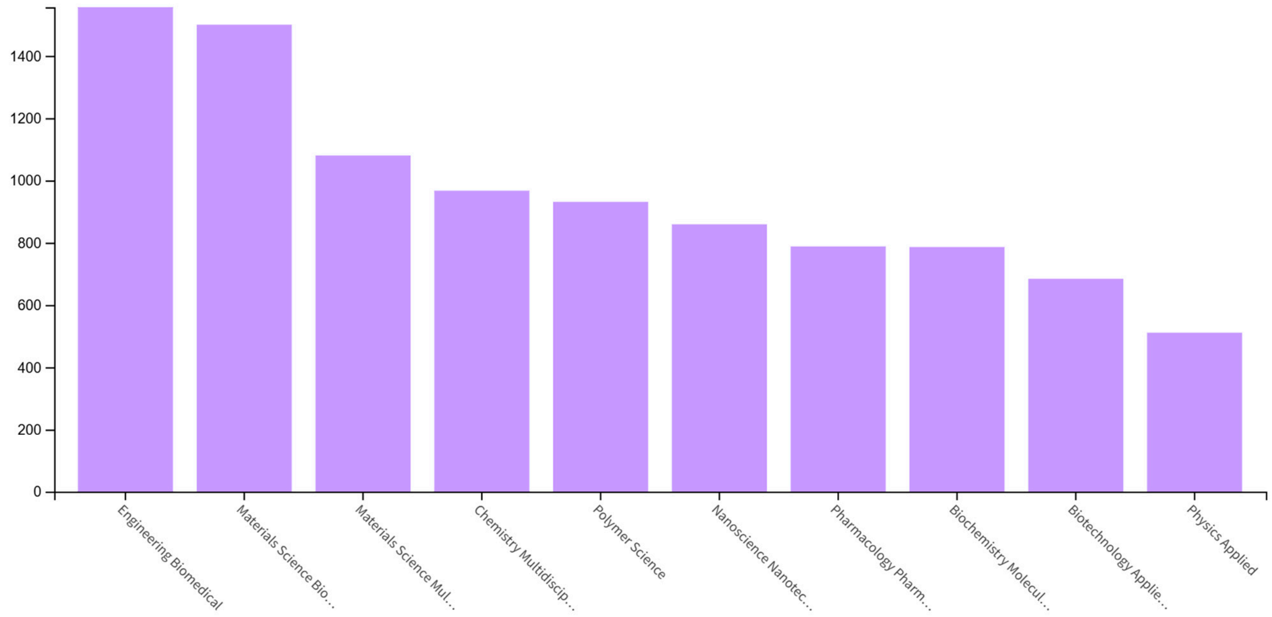

Figure S2. Bar-chart showing Top 10 WoS Categories (specific research fields) approached in the papers belonging to Db7251

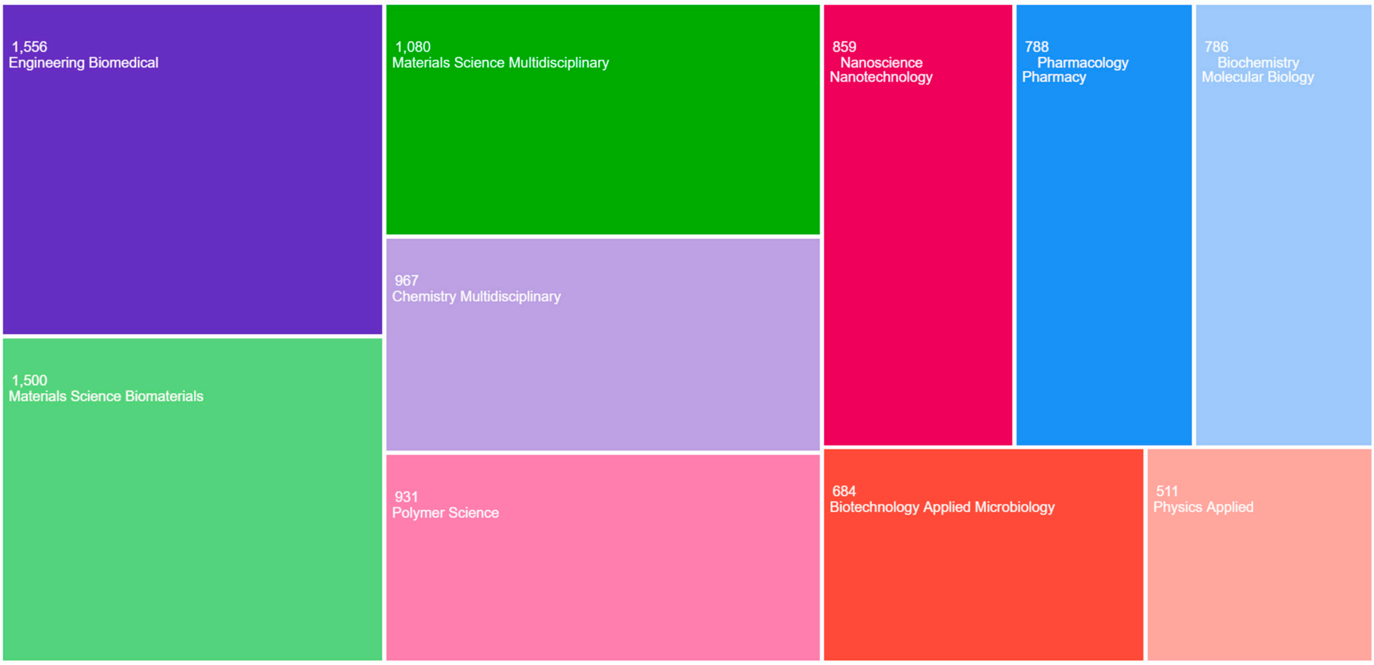

Figure S3. Tree-map chart showing Top 10 WoS Categories (specific research fields) approached in the papers belonging to Db7251. The areas on the chart are not strictly proportional to the values of each entry.

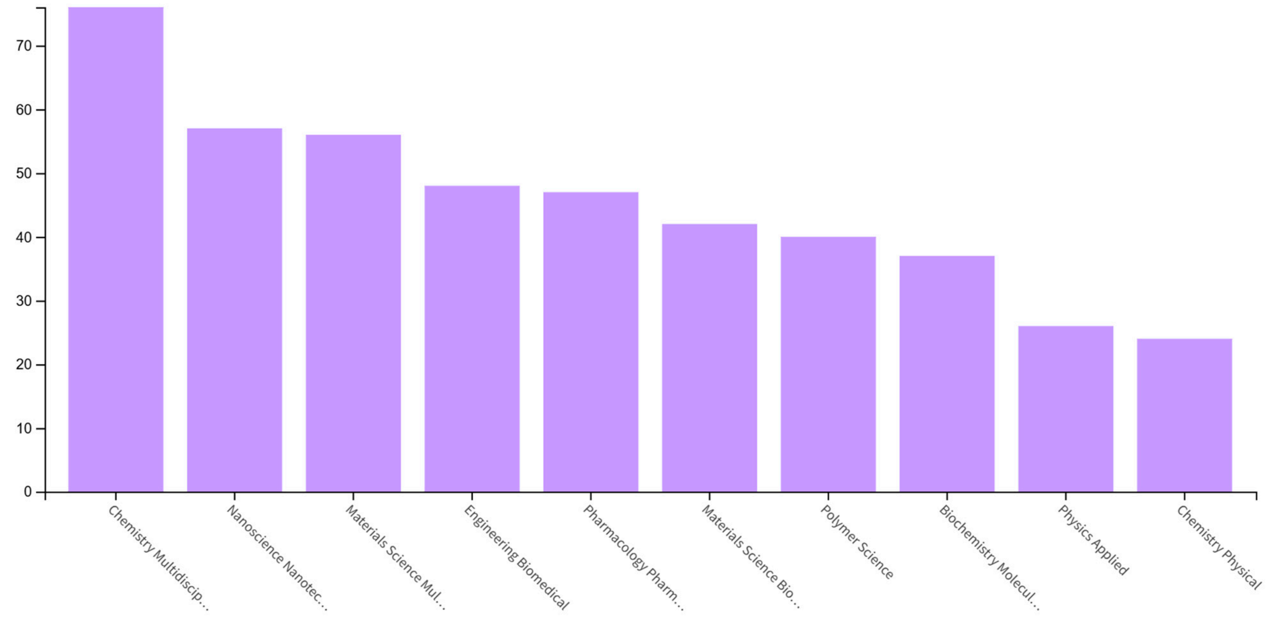

Figure S4. Bar-chart showing Top 10 WoS Categories (specific research fields) approached in the papers belonging to Db326

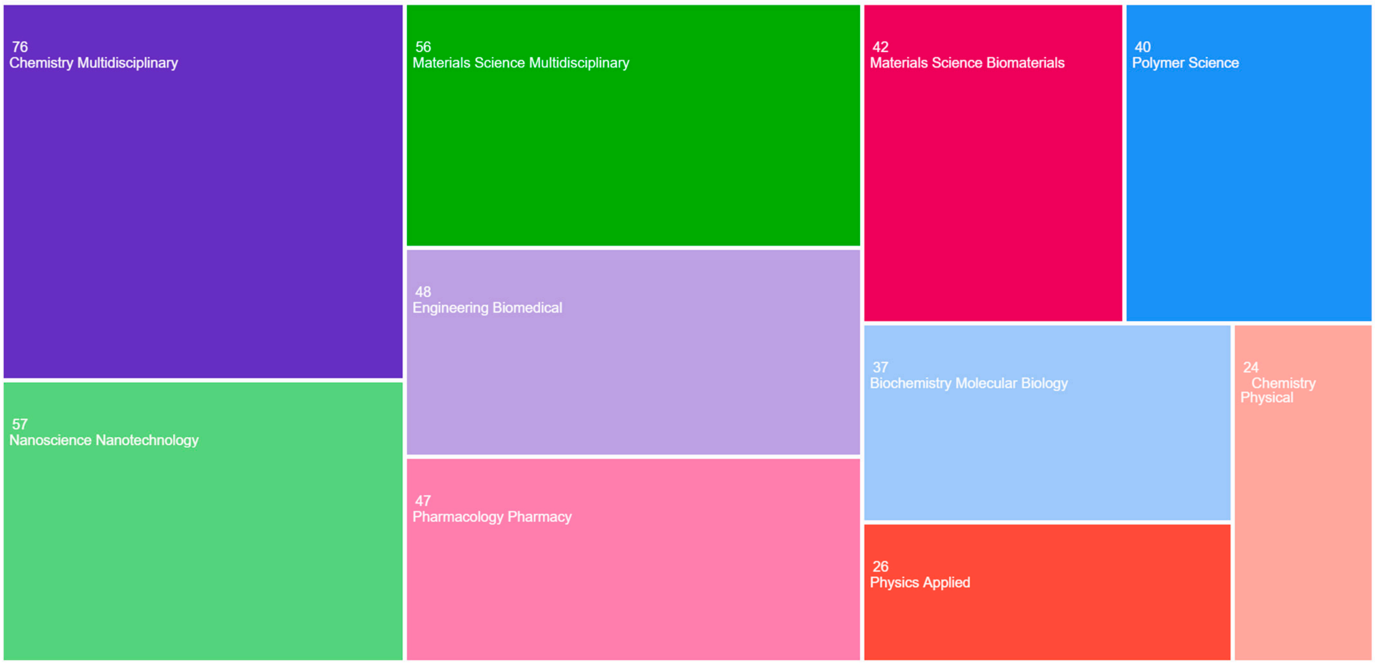

Figure S5. Tree-map chart showing Top 10 WoS Categories (specific research fields) approached in the papers belonging to Db326. The areas on the chart are not strictly proportional to the values of each entry.

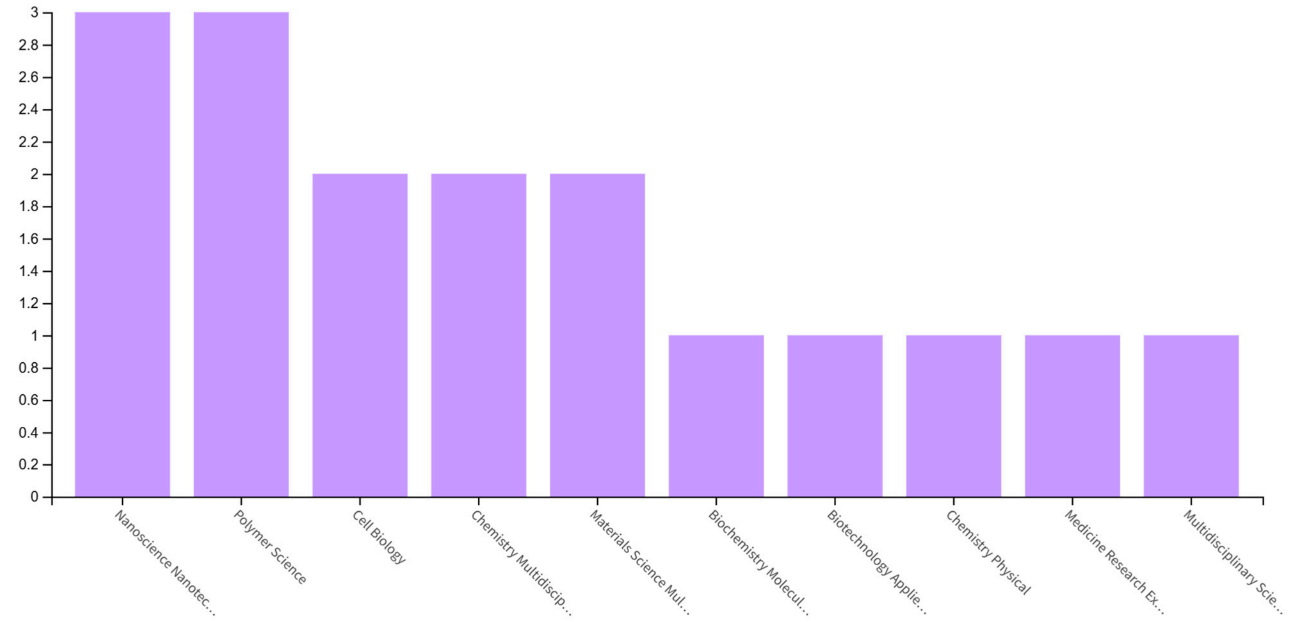

Figure S6. Bar-chart showing Top 10 WoS Categories (specific research fields) approached in the papers belonging to Db11

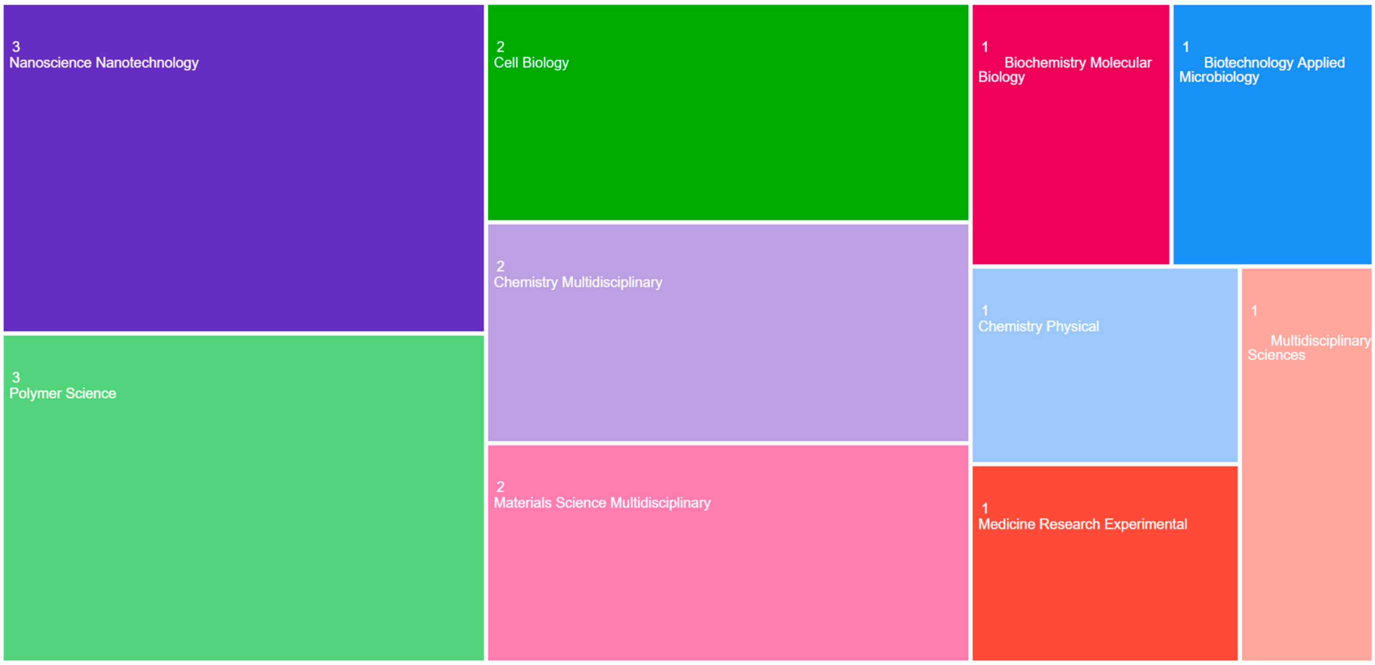

Figure S7. Tree-map chart showing Top 10 WoS Categories (specific research fields) approached in the papers belonging to Db11. The areas on the chart are not strictly proportional to the values of each entry.

Table S1. Db11 – WoS generated bibliometric database containing 11 highly cited & hot reviews

No.	Authors	Article Title	Source Title	Publisher	Publication Year	DOI Link	WoS Categories	Research Areas
**1**	Satchanska, G; Davidova, S; Petrov, PD	Natural and Synthetic Polymers for Biomedical and Environmental Applications	POLYMERS	MDPI	2024	http://dx.doi.org/10.3390/polym16081159	Polymer Science	Polymer Science
**2**	Segneanu, AE; Bejenaru, LE; Bejenaru, C; Blendea, A; Mogosanu, GD; Bita, A; Boia, ER	Advancements in Hydrogels: A Comprehensive Review of Natural and Synthetic Innovations for Biomedical Applications	POLYMERS	MDPI	2025	http://dx.doi.org/10.3390/polym17152026	Polymer Science	Polymer Science
**3**	Yuan, X; Zhu, W; Yang, ZY; He, N; Chen, F; Han, XX; Zhou, K	Recent Advances in 3D Printing of Smart Scaffolds for Bone Tissue Engineering and Regeneration	ADVANCED MATERIALS	WILEY-V C H VERLAG GMBH	2024	http://dx.doi.org/10.1002/adma.202403641	Chemistry, Multidisciplinary; Chemistry, Physical; Nanoscience & Nanotechnology; Materials Science, Multidisciplinary; Physics, Applied; Physics, Condensed Matter	Chemistry; Science & Technology - Other Topics; Materials Science; Physics
**4**	Zhao, JX; Zhu, MY; Jin, W; Zhang, JL; Fan, GY; Feng, YF; Li, Z; Wang, SM; Lee, JS; Luan, GX; Dong, ZQ; Li, Y	A comprehensive review of unlocking the potential of lignin-derived biomaterials: from lignin structure to biomedical application	JOURNAL OF NANOBIOTECHNOLOGY	BMC	2025	http://dx.doi.org/10.1186/s12951-025-03604-7	Biotechnology & Applied Microbiology; Nanoscience & Nanotechnology	Biotechnology & Applied Microbiology; Science & Technology - Other Topics

**5**	Boufidis, D; Garg, R; Angelopoulos, E; Cullen, DK; Vitale, F	Bio-inspired electronics: Soft, biohybrid, and living neural interfaces	NATURE COMMUNICATIONS	NATURE PORTFOLIO	2025	http://dx.doi.org/10.1038/s41467-025-57016-0	Multidisciplinary Sciences	Science & Technology - Other Topics
**6**	Gonciarz, W; Balcerczak, E; Brzezinski, M; Jelen, A; Pietrzyk-Brzezinska, AJ; Narayanan, VHB; Chmiela, M	Chitosan-based formulations for therapeutic applications. A recent overview	JOURNAL OF BIOMEDICAL SCIENCE	BMC	2025	http://dx.doi.org/10.1186/s12929-025-01161-7	Cell Biology; Medicine, Research & Experimental	Cell Biology; Research & Experimental Medicine
**7**	Lu, PL; Ruan, DX; Huang, MQ; Tian, M; 8Zhu, KS; Gan, ZQ; Xiao, ZC	Harnessing the potential of hydrogels for advanced therapeutic applications: current achievements and future directions	SIGNAL TRANSDUCTION AND TARGETED THERAPY	SPRINGER-NATURE	2024	http://dx.doi.org/10.1038/s41392-024-01852-x	Biochemistry & Molecular Biology; Cell Biology	Biochemistry & Molecular Biology; Cell Biology
**8**	Yang, J; Zeng, HY; Luo, YS; Chen, Y; Wang, M; Wu, CB; Hu, P	Recent Applications of PLGA in Drug Delivery Systems	POLYMERS	MDPI	2024	http://dx.doi.org/10.3390/polym16182606	Polymer Science	Polymer Science
**9**	Guo, XH; Luo, WK; Wu, LY; Zhang, LL; Chen, YX; Li, T; Li, HG; Zhang, W; Liu, YW; Zheng, J; Wang, Y	Natural Products from Herbal Medicine Self-Assemble into Advanced Bioactive Materials	ADVANCED SCIENCE	WILEY	2024	http://dx.doi.org/10.1002/advs.202403388	Chemistry, Multidisciplinary; Nanoscience & Nanotechnology; Materials Science, Multidisciplinary	Chemistry; Science & Technology - Other Topics; Materials Science
**10**	Javid, H; Oryani, MA; Rezagholinejad, N; Esparham, A; Tajaldini, M; Karimi-Shahri, M	RGD peptide in cancer targeting: Benefits, challenges, solutions, and possible integrin-RGD interactions	CANCER MEDICINE	WILEY	2024	http://dx.doi.org/10.1002/cam4.6800	Oncology	Oncology
**11**	Baral, KC; Choi, KY	Barriers and Strategies for Oral Peptide and Protein Therapeutics Delivery:	PHARMACEUTICS	MDPI	2025	http://dx.doi.org/10.3390/pharmaceutics17040397	Pharmacology & Pharmacy	Pharmacology & Pharmacy

	Update on Clinical Advances				

Table S2. Polymers, biopolymers & advanced materials databases

No.	Database name & link	Description	Team	Start Year	Uses AI/ML
1	**Materiom**<br>www.materiom.org	The Materiom Commons is an open-access database and community platform supporting biobased materials innovation for packaging, textiles, durable goods, the built environment and more.	Materiom is a start-up founded by interdisciplinary researchers, engineers, AI and communication specialists focused on materials which enable circular economy (have a net-positive impact on the planet). They offer open data and AI software. The stakeholders form a network across the materials value chain, including scientists, commercial material developers, consumer brands, retailers, recyclers, composters and local communities.	2023	yes
2	**BIOMATDB**<br>www.biomatdb.eu<br><br>Database:<br>biomaterialdatabase.com<br>Marketplace:<br>www.biomaterialmarketplace.com	BIOMATDB aimed to create an Advanced Database and Marketplace for Biomaterials, providing detailed information on their properties. It also aimed to support selling companies, especially SMEs, in properly presenting themselves and their products at global scale. Another target of the database is to show the label of biocompatibility to define the suitability of a biomaterial for use in a medical device or advanced therapy, in order to guide buyer companies in need for such products in their fabrication line.	The BIOMATDB consortium brings together 12 partners from 8 countries (Austria, Finland, Ireland, Italy, Norway, Poland, Portugal, Spain) with expertise in polymer and biomaterials science, biomechanics and tissue engineering, medicine and health technology, materials science and engineering, medical device research, emerging therapies and technologies in medicine, cooperation and international relations, as well as ethical and social impact assessments. The partners represent Universities, enterprises, public research centers, hospitals, pioneer independent researchers (consultants), research-focused NGOs, business councils/ chambers of commerce (governmental parties), health clusters covering SMEs, big companies, regional stakeholders and digital health international organizations<br>Horizon Europe 06/2022 – 11/2024	2022	yes
3	**Cloud Materials**<br>www.materialscloud.org	Materials Cloud is built to enable the seamless sharing and dissemination of resources in computational materials science, offering educational, research, and archiving	Today, Materials Cloud is supported by a consortium of partners, including a Go-FAIR implementation network. It is a recommended repository of the Swiss National Science Foundation, European Commission through Open	2020	yes

		tools; simulation software and services; and curated and raw data. It uses Quantum Mobile (a Virtual Machine for computational materials science) virtual machine and AiiDA (a python framework for automated workflows in materials' informatics and provenance tracking).	Research Europe and of Nature Scientific Data, and remains open to further partnerships.		
**4**	**DEBBIE** https://projectdebbie.github.io	The Database of Experimental Biomaterials and their Biological Effect was meant to be an open access database of biomaterials automatically curated from the scientific literature. The project behind the database also aimed to create and optimize text mining tools for the biomaterials domain.	EU researchers from different Universities and Research Centers. The project received funding from the European Union's Horizon 2020 research and innovation programme under the Marie Sklodowska-Curie grant agreement No 751277.	2020	yes
**5**	**NOMAD** Website https://nomad-lab.eu/ Database https://nomad-lab.eu/prod/v1/gui/search/entries	NOMAD is a free web-service for browsing, sharing, organizing, analyzing, downloading and publishing materials science data. NOMAD is the largest database of its kind, combining data from popular sources like the Materials Project, AFLOW, and OQMD.	German team	2014	yes
**6**	**MatWeb** www.matweb.com	Searchable online free database of engineering materials and their properties (thermoplastic and thermoset polymers, ceramics, semiconductors, fibers, metals and so on). Contains over 180,000 data sheets and spec sheets from manufacturers and distributors. Includes convertors and calculators for engineering tasks.	It was started in the mid-1990's by a group of engineers in need of a comprehensive database with material properties to avoid searching through articles, books and faxes. In time, they founded Automation Creations. In 2011, MatWeb LLC was spun off from that to focus solely on the engineering materials database business.	2011	Yes
**7**	**The Materials Project** https://next-gen.materialsproject.org	The Materials Project provides open web-based access to computed information on known and predicted materials as well as powerful analysis tools to inspire and design novel materials. It aims to compute the properties of all inorganic materials and provide the data and associated analysis algorithms for every materials researcher free of charge. The project was established in 2011 with an emphasis on battery research, but includes property calculations for many areas of clean energy systems such as photovoltaics, thermoelectric materials, and catalysts. It offers several apps to explore and search and several free and open-source software projects that both power the	The Materials Project is a multi-institution, multi-national effort. Its team is formed by Professors, researchers, engineers and experts in Materials Science and Engineering, Computing Science and Operations, Materials Algorithms, Data and Experimental Validation.	2011	yes

		Materials Project apps and support the scientific community at large.			
8	**Genome Materials Initiative (MGI)**<br>www.mgi.gov	This project aims to discover, manufacture and deploy advanced materials twice as fast and at a fraction of the cost compared to traditional methods. Its goals are: 1. To unify the Materials Innovation Infrastructure; 2. To harness the power of materials data; 3. To educate, train, and connect the materials R&D workforce.	The Materials Genome Initiative is a federal multi-agency initiative (part of National Institute of Standards and Technology, US Department of Commerce).	2011	yes
9	**PoLyInfo**<br>https://polymer.nims.go.jp -><br><br>part of **MatNavi**<br>https://mits.nims.go.jp<br>-><br><br>which includes 8 sub-databases:<br>1. The Polymer Database (PoLyInfo)<br>2. The Inorganic Materials Database (AtomWork)<br>3. The Computational Phase Diagram Database (CPDDB)<br>4. The Computational Electronic Structure Database (CompES-X)<br>5. The Diffusion Database (Kakusan)<br>6. The Thermophysical Property Database<br>7. The Metallic Material Database (Kinzoku)<br>8. The CCT Diagram Database (CCTD)<br><br>part of **DICE**<br>https://dice.nims.go.jp	Polymer Database "PoLyInfo" systematically provides various data (properties, chemical structures, IUPAC names, processing methods of measured samples, measurement conditions, used monomers and polymerization methods) required for polymeric material design.<br>It is one of the databases encompassed by DICE, a digital innovative collaborative ecosystem for materials research provided by the NIMS Materials Data Platform (MDPF). NIMS is the National Institute for Materials Science in Japan.<br>DICE includes "MatNavi," one of the world's largest materials databases; "RDE," which structures, stores, and shares research data; "MDR," a repository of materials-related literature and data; "pinax," an analysis system with AI functions; and "Mint," an integrated platform for industry-academia-government collaboration. We provide data services to encourage efficiency, speed, and sophistication in materials development.	Japanese team of researchers	1995	yes

References **[1–15]**

Table S3. Wound dressing solutions: skin substituents

Model	Composition	Indications for use	Status	Company	Fabrication Country	Marketing Authorization	Temporary (removable) / Temporary (bioresorbable, biodegradable) / Permanent (integrating graft)	Acellular / Cellular	Cryo-preserved / Fridge / Room temp (RT)	Type of skin substituent
**I. Acellular skin substitutes**										
I.1 Acellular dermal replacement from donated human dermis										
AcelloDerm™	AcelloDerm™ is a pre-hydrated, human acellular dermal matrix allograft sourced from human skin. SAL of 10^{-3}. It undergoes aseptic processing and terminal sterilization to maintain the natural collagen microstructure, while effectively removing immunogenic cells and epidermis. AcelloDerm™ is the closest to unprocessed dermis and supports a lower inflammatory response and superior tissue regeneration compared to other commercially available acellular dermal matrices.<br>2-4 years shelf life	It preserves the structural integrity of ECM components, including collagen, elastin, and glycosaminoglycan (GAG). These components create an ideal microenvironment for tissue remodeling in regenerative medicine applications, providing mechanical strength, collagen integrity, and bioactive elements akin to unprocessed dermis. It also maintains the basement membrane to prevent adhesion formation and a basement	Marketed	Berkeley Biologics LLC (a subsidiary of GNI Group), following acquisition of the orthobiologics business unit from Elutia (formerly Aziyo Biologics) in 2023	USA	USA	permanent (integrating)	acellular	RT	hADM

		membrane complex of blood vessels to promote angiogenesis.								
AlloDerm™ regenerative tissue matrix<br><br>AllodermSelect™<br><br>Alloderm Select Restore™<br><br>Alloderm Select Duo™<br><br>Alloderm Select Restore™	Decellularized donated human cadaveric dermis, processed to remove cells while preserving biologic component and the structure of dermal matrix. Acellular dermal matrix (ADM) with SAL of 10^{-3} that supports regeneration.<br>LifeCell Corporation developed freeze-dried AlloDerm™ RTM in 1994. AlloDerm SELECT™ RTM, a sterile and ready-to-use product, which utilizes the same core processing, first became available in 2010.<br>2-year shelf life	Primarily used in reconstructive and plastic surgery, including breast and dental reconstruction (gums – periodontology). Is is also used in hernia repair.	Marketed	manufacturer: LifeCell Corporation, an AbbVie company<br>distributor for dental applications: BioHorizons	USA	USA	permanent (integrating)	acellular	RT	RTM
AlloMend®<br><br>AlloMend® Duo<br><br>AlloMend® Ultra Thick<br><br>AlloMend® Extra-Large	AlloMend ADM offers a flexible and reliable graft for demanding soft tissue applications. It features ultimate tensile strength exceeding that of leading acellular matrices, and high suture retention strength for more confidence in surgical repair of soft tissue.	Used in: breast reconstruction, pelvic organ prolapse, tendon augmentation, rotator cuff repair, superior capsular reconstruction, fat pad replacement, hernia repair, bicep tendon repair.	Marketed	AlloSource	USA	USA	permanent (integrating)	acellular	RT	hADM

AlloMend® Mesh Shaped	Terminal sterilization with e-beam technology to a Sterility Assurance Level (SAL) of 10-6. Available in a variety of thicknesses and sizes for a wide range of surgical applications, pre-hydrated.	The DUO variant represents a dual-sided reticular dermal matrix for non-directional implantation. The Ultra-Thick variant is a flexible and reliable graft for demanding soft tissue applications such as superior capsule reconstruction. The Extra-Large variant features the largest size available in an area of 320cm2 for demanding soft tissue applications. The meshed variant with 1:1 meshing ratio increases surface area 97.5% for faster fluid egress and potential incorporation.								
AlloPatch® Pliable<br><br>AlloPatch® Pliable Meshed	AlloPatch is human allograft skin minimally processed to remove epidermal and dermal cells. Aseptically processed, not terminally sterilized. The process utilized preserves the extracellular matrix of the dermis.	They serve as a frameworks to support cellular repopulation and vascularization at the surgical site; chronic or acute wound covering.	Marketed	MTF Biologics, a registered trademark of the Musculoskeletal Transplant Foundation	USA	USA	permanent (integrating)	acellular	RT	hADM

	3-year shelf life at ambient temperature									
Alloskin™ AC	AlloSkin AC is a meshed dermis-only human skin graft that has been decellularized while preserving the natural biologic components and structure of the dermal matrix.<br>2-year shelf life	The graft provides a favorable microenvironment for bio-ingrowth to begin revascularization and cellular repopulation. Single application is often sufficient to potentially help stimulate the wound healing process. AlloSkin AC is especially suited for permanent incorporation in a debrided burn or wound bed to fill deficits, aiding in contouring and cosmesis, and covering exposed bones and tendons. Pliable and stretchable for contouring to wound topography and maintenance of wound bed contact. Robust enough to suture or staple. Meshed (1:1) encouraging fluid drainage from wound.	Marketed	AlloSource	USA	USA	permanent (integrating)	acellular	RT	hADM

Coll-e-derm™	Coll-e-derm is a prehydrated human acellular dermal matrix that retains angiogenin and collagen type IV, thus supporting revascularization in wound healing. Terminally Sterile at 10^{-6} SAL with low dose precision gamma irradiation, lowering risk of infection.<br>> 2-year shelf life	Coll-e-Derm maintains the strength of human skin and provides a natural scaffold for cellular and capillary remodelling of a damaged site, which can expedite the healing process. Coll-e-Derm™ is broadly indicated for the management of topical wounds, including diabetic foot ulcers and venous leg ulcers, but also for rotator cuff repair, lateral ankle stabilization, and hip labral repairs.	Marketed	Parametrics Medical	USA	USA	permanent (integrating)	acellular	RT	hADM
DermACELL® Human Acellular Dermal Matrix<br><br>DermACELL AWM	DermACELL® Human Acellular Dermal Matrix (hADM) acts as a permanent, bioactive scaffold that is incorporated into the body's tissue rather than being a temporary covering. It is a hydrated matrix, remodeled and replaced by the patient's own cells and collagen over time. It provides a long-term, structural matrix for tissue regeneration. It is terminally sterilized-rendering the graft	DermACELL (General): A portfolio of processed human tissue grafts (dermal matrices) used for various applications, including surgical reconstruction, breast reconstruction, and chronic wounds.<br>DermACELL AWM: Specifically engineered and marketed for chronic wound care,	Marketed	LifeNet Health	USA	USA	permanent (integrating)	acellular	RT	hADM

	sterile to medical device-grade standards with a SAL of 10-6.<br>1.5-4 years shelf life depending on product configuration (meshed vs unmeshed)	such as diabetic foot ulcers (DFUs), venous stasis ulcers (VSUs), arterial ulcers and pressure ulcers, as well as dehisced surgical wounds and traumatic burns. It can be used over exposed tendon, bone, joint capsule, and muscle. It is optimized for rapid cellular infiltration and re-vascularization.								
Dermapure®<br><br>DermaPure® Non-Oriented<br><br>DermaPure® Meshed	Decellurized human dermis product, using the patented dCELL® Technology process to create a natural biological scaffold that is up to 99% DNA-free. It provides a scaffold into which the recipient's cells can grow, becoming vascularized and supporting the generation of a new epithelial layer.<br>2-year shelf life	It is used for native skin regeneration / wound healing in: traumatic injuries, burns, head / neck reconstruction, DFUs/VLUs, pilonidal cysts, hidradenitis suppurativa, necrotizing fasciitis, diabetic limb salvage, acute and chronic wounds, orthopaedics, podiatry, foot and ankle surgery, sports medicine, pelvic floor surgery (Uro-Gyn, Urology, ColoRectal)	marketed	Tissue Regenix Group	USA	USA<br>UK<br>EU	permanent (integrating)	acellular	RT	hADM

DermaSpan™ Acellular Dermal Matrix	Sterile, decellularized dermal allograft product.<br>2-3 years shelf life	It can be used in orthopedics, plastic surgery, and general surgery, to repair or replace damaged or inadequate integumental tissue (wound coverage). DermaSpan ACD can also be used for supplemental support, protection, reinforcement, or covering of tendon.	Marketed	manufactured by Biomet Orthopedics marketed by Zimmer Biomet	USA	USA	permanent (integrating)	acellular	RT	hADM
FlowerDerm™	FlowerDerm is a meshed dermis-only decellularized human skin graft that preserves the natural biologic components and structure of the dermal matrix.<br>2-year shelf life.	A single application of this product is often sufficient to stimulate the wound healing process. The pliable, flexible material adheres to wound topography and maintains contact with the wound bed. It's also durable enough to suture or staple and has a mesh construction to encourage fluid drainage. FlowerDerm™ Meshed form is intended for diabetic foot ulcer, venous foot ulcer etc.	marketed	Flower Orthopedics	USA	USA	permanent (integrating)	acellular	RT	hADM

GraftJacket™ RTM	It is a human dermal collagen matrix that is readily incorporated into the body. The matrix undergoes a patented process that renders the material essentially acellular and is freeze-dried with a proprietary process that prevents the formation of ice crystals to preserve the intact matrix, including vascular channels.<br>2-year shelf life.	GraftJacket Matrix is used to provide supplemental support, protection, and reinforcement of tendon and ligamentous tissue; to be used as a periosteal patch or covering; or for protection and support of bone and tendons in foot and ankle and hand surgery.<br>An excellent scaffold to reinforce primary soft-tissue repairs throughout the body while eliminating morbidity associated with harvesting autograft.	Marketed	Wright Medical Group N.V.	USA	USA	permanent (integrating)	acellular	RT	RTM
hMatrix® ADM	hMatrix ADM is an allograft derived from donated human skin. The dermis is processed using a proprietary method to remove the cells to maximize graft incorporation. hMatrix ADM is provided as a frozen, sterile product with a device-level (SAL) of 10-6.<br>5-year shelf life in frozen storage	The successful treatment of deep wounds often requires the use of a dermoconductive graft material to facilitate formation of granulation tissue and aid in wound closure. hMatix ADM offers superior suture retention strength, flexible matrix for precise	Marketed	Bacterin International, Inc.	USA	USA	permanent (integrating)	acellular	cryo-preserved	hADM

		placement and lower inflammatory response vs. competitors.								
InteguPly®	InteguPly is a dehydrated human acellular dermis (ACD) processed to maintain the biologic and structural integrity of the tissue's extracellular matrix components, while delivering a SAL of 10-6.<br>5-year shelf life	Supports the repair or replacement of integumental tissue, as well as closure of chronic diabetic foot ulcers, venous leg ulcers and pressure wounds (wound care and orthopedic sports medicine). It provides support, protection and reinforcement in a number of orthopedic sports medicine applications including tendon, ligament, capsule and rotator cuff.	Marketed	Berkeley Biologics LLC (a subsidiary of GNI Group), following acquisition of the orthobiologics business unit from Elutia (formerly Aziyo Biologics) in 2023	USA	USA	permanent (integrating)	acellular	RT	dhADM
Matrix HD® Allograft	Matrix HD allograft is an acellular human dermis allograft sterilized using the Tutoplast™ tissue Sterilization process. This proprietary process retains the 3-dimensional intertwined multidirectional fibers and mechanical properties of the native dermis tissue. Terminally sterilized to a SAL of	The Matrix HD graft provides a natural scaffold to support the body's regenerative process.<br>Biocompatible: Preserved vascular channels. Preserved key components of the native matrix.	Marketed	RTI Surgical	USA	USA	permanent (integrating)	acellular	RT	hADM

	10–6.<br>5-year shelf life	Simple, single-step rehydration.								
SimplyDerm®	SimpliDerm is a pliable, easy-to-handle hydrated human acellular dermal matrix (hADM). This allograft comes in a perforated and a non-perforated option, with a SAL of 10^{-6}.<br>5-year shelf life	It is designed specifically to promote rapid repopulation and revascularization for enhanced healing and integration. With its perforated and non-perforated options, it offers an innovative allograft portfolio designed to meet diverse surgical needs and promote healthy tissue remodeling.	Marketed	Elutia (formerlyAziyo Biologics)	USA	USA	permanent (integrating)	acellular	RT	hADM
SomaGen® Meshed	SomaGen® Meshed is an acellular human reticular dermal allograft with a unique meshed design that provides a scaffold to support the treatment of a variety of complex wounds.<br>>2 years shelf life	SomaGen Meshed is designed for large and complex wounds. The lattice-based slit design allows the graft to conform and expand based on the wound size and topography.	Marketed	AlloSource	USA	USA	permanent (integrating)	acellular	RT	hADM

I.2 Acellular dermal replacement from human placental membrane										
AlloWrap®<br>AlloWrap® DS<br>AlloWrap® Dry	AlloWrap is a human amniotic membrane containing bioactive proteins that support wound healing. Strong, pliable tissue conforms to wound topography. Available in a moist configuration, especially suitable for endoscopic and minimally invasive surgical applications (AlloWrap DS), or dry / dehydrated, for precision open-surgical placement (AlloWrap Dry).<br>2-year shelf life	It provides an immune-privileged barrier to support the patient's body in preventing inflammation and scar tissue generation.<br>While it remains in the surgical site for an extended period—lasting greater than eight weeks (persisting during the proliferation and remodelation phases of wound healing)—it is not considered a permanent, lifetime implant.	Marketed	AlloSource	USA	USA	temporary (bioresorbable)	acellular	RT	hAM / dhAM
AmnioBand®	AmnioBand is a temporary (biodegradable), single-use, dehydrated human amniotic/chorion allograft designed to act as a scaffold for wound healing, not a permanent implant, that retains the structural properties of the extracellular matrix. It is used as a covering that eventually breaks down as the underlying tissue heals. It can be used in the hydrated	Uses: wound healing and host tissue remodeling<br>It serves as a wound covering. It is typically applied to the wound weekly or as needed. It is meant to be used for 12 weeks to facilitate closure.<br>It helps bridge the gap to healing by providing a, scaffold that,	Marketed	MTF Biologics, a registered trademark of the Musculoskeletal Transplant Foundation	USA	USA	temporary (bioresorbable)	acellular	RT	dhACM

	or dehydrated state.<br>3-year shelf life	promotes tissue regeneration and, reduces inflammation.								
AmnioExcel® Amniotic Allograft Membrane<br><br>AmnioExcel® Plus Placental Allograft Membrane	AmnioExcel® is dehydrated human amnion-derived tissue allograft with intact extracellular matrix, which acts as a scaffold and protective barrier in skin repair and regeneration. It eventually breaks down and get absorbed by the body as the wound heals.<br>AmnioExcel® Plus is an allograft tissue intended for homologous use as a protective barrier covering during the repair of soft tissue wounds at the direction of a physician and is 2x strong as AmnioExcel®.<br>AmnioExcel®: 2-year shelf life<br>AmnioExcel® Plus: 5-year shelf life	Both are intended for use as a wound covering to aid in closing chronic wounds. AmnioExcel is often applied in a series of sessions to chronic wounds that have failed to respond to standard care, with total treatment lasting for a limited period, typically up to 16 weeks.	Marketed	Integra LifeSciences Corp., following acquisiton of Derma Sciences in 2017	USA	USA	temporary (bioresorbable)	acellular	RT	dhAM

AmnioFill® Human Placental Tissue Allograft	AmnioFill is a nonviable cellular tissue matrix allograft (human collagen matrix derived from the amnion and chorion) that contains multiple extracellular matrix proteins, growth factors, cytokines, and other specialty proteins present in placental tissue to help enhance healing. Terminally sterilized for enhanced patient safety.<br>5-year shelf life	It is used to manage acute and chronic wounds, or as a soft tissue covering, to reduce inflammation and scar formation, facilitating faster healing before being absorbed. It has a versatile tissue form.	Marketed	MiMedx Group	USA	USA	temporary (bioresorbable)	acellular	RT	dhACM
AmnioFix® Amnion/Chorion Membrane Allograft	Group AmnioFix is a temporary (biodegradable) product, a bioactive tissue matrix allograft composed of dehydrated human amnion/chorion membrane (dHACM) that preserves and contains multiple extracellular matrix proteins, growth factors, cytokines, and other specialty proteins. It gets reborbed / remodeled in 3-4 week of application. Terminally sterilized for enhanced patient safety.<br>5-year shelf life	AmnioFix acts as a temporary barrier that supports the body's natural healing process, reduces inflammation, and minimizes scar tissue formation. It enhances surgical wound healing and acts as a barrier membrane.	Marketed	MiMedx Group	USA	USA	temporary (bioresorbable)	acellular	RT	dhACM

Amniomatrix® Human Amniotic Suspension Allograft	It is a a cryopreserved, liquid-based, injectable biologic product derived from the human amniotic membrane and amniotic fluid. It is cryopreserved using the patented CryoPrime™ processing method that preserves the structural properties of the collagen, cytokines, growth factors, ECM, and viable cellular materials. Should be stored at -65°C or below.<br>3 months shelf life	It is designed for homologous use in the repair, reconstruction, and replacement of soft tissue, particularly for treating complex, deep, or tunneling wounds.<br>It is generally applied to stimulate the healing process rather than to provide permanent structural replacement. Similar to other amniotic tissue grafts, the material is generally incorporated or resorbed within a few weeks (often within 4-6 weeks) to a few months, rather than becoming a permanent part of the tissue.	Marketed	Integra LifeSciences Corp., following acquisition of Derma Sciences in 2017	USA	USA<br>Canada<br>Latin America<br>Asia	temporary (bioresorbable)	acellular	cryo-preserved	hAMS

Artacent® Wound<br><br>Artacent® Wound C<br><br>Artacent® Wound Trident<br><br>Artacent® Wound AC<br><br>Artacent® Wound VeriClen<br><br>Artacent® Wound Velos	Dehydrated, sterilized, human amniotic or amniotic-chorion membrane allografts. They are available in single, dual, and triple-layer formats (e.g., Artacent AC is a tri-layer graft). They are designed as sterile, dry-storage, cellular-based tissue substitutes for acute and chronic wounds, allowing for shelf-stable, room-temperature storage without needing refrigeration.<br>C: single layer chorion membrane<br>Trident: triple layer human amniotic membrane allograft<br>AC: tri-layer, human amniotic graft<br>VeriClen: full thickness amniotic tissue graft sterilized via e-beam<br>Velos: dual layer human amniotic membrane allograft<br>5-year shelf life	These grafts are used as protective coverings for various wounds, including diabetic ulcers and surgical sites.<br><br>Artracent is the only wound-specific amniotic patch that can be applied with either side facing the wound. It is applied as a patch, remains in place without sutures, and gradually helps the body's own tissue regenerate, rather than replacing it permanently.	Marketed	Tides Medical	USA	USA	temporary (bioresorbable)	acellular	RT	dhAM / dhACM
BioDFactor® Viable Tissue Matrix	It is a flowable tissue allograft derived from morselized amniotic tissue and components of the amniotic fluid.<br>2+ years shelf life (even 5+)	It is marketed for use as a wound covering in the treatment of complex chronic wounds, acute wounds, and localized areas of	Marketed	Integra LifeSciences Corp., following acquisition of	USA	USA	temporary (bioresorbable)	acellular	cryo-preserved	hAMS

		inflammation, as well as for filling soft tissue defects or voids.<br>Applications: wound care, orthopedics, and surgical applications.		Derma Sciences in 2017						
BioDDryFlex® Amniotic Tissue Membrane	BioDDryFlex is a single-layer amniotic allograft for applications in which bulk may not be optimal. This extracellular membrane may be hydrated prior to, or following, placement.<br>2-year shelf life	Management of damaged dermal tissue or soft tissue defect	marketed	Integra LifeSciences, following acquisition of BioD, LLC & its subsidiary, bioDlogics in 2017 (they were already part of Derma Science since 2016)	USA	USA	temporary (bioresorbable)	acellular	RT	dhAM
BioDfence® G3 Placental Tissue Membrane	BioDFence G3 is a dehydrated, tri-layer amnion-chorion-amnion allograft providing enhanced handling characteristics.<br>5-year shelf life	Amnion-chorion-amnion surgical use, where adjustments and manipulation are required	marketed	Integra LifeSciences, following acquisition of BioD, LLC & its subsidiary, bioDlogics in 2017 (they were already part of	USA	USA<br>Canada	temporary (bioresorbable)	acellular	RT	dhACM

				Derma Science since 2016)						
BioDFence® Sentry Placental Tissue Membrane	BioDFence® Sentry Placental Tissue Membrane is a dehydrated, tri-layer amnion, chorion, amnion allograft providing enhanced handling characteristics for surgical application where *in vivo* adjustments and manipulation are required.<br>5-year shelf life	It serves as a soft tissue barrier for use during surgical procedures.	Marketed	Integra LifeSciences, following acquisition of BioD, LLC & its subsidiary, bioDlogics in 2017 (they were already part of Derma Science since 2016)	USA	USA<br>Canada	temporary (bioresorbable)	acellular	RT	dhACM
Biovance® Amniotic Membrane Allograft	BIOVANCE® is a decellularized, dehydrated human amniotic membrane allograft that provides a protective cover from the surrounding environment. It offers wound visualization.<br>10-year shelf life	Biovance is an allograft intended for use as a biological membrane covering that provides the ECM while supporting the repair of damaged tissue. It is intended for chronic wounds.	Marketed	Cellularity, Inc.	USA	USA<br>UAE	temporary (bioresorbable)	acellular	RT	dhAM
Cellesta Amniotic Membrane	It is a single-layered, amnion (chorion-free) graft that is typically affixed to a poly mesh backing, which is	Used as a natural human tissue scaffold for wound care,	marketed	Ventris Medical	USA	USA	temporary (bioresorbable)	acellular	RT	dhAM

	removed at application.<br>2-5 years shelf life	orthopedics, and surgical applications								
Cygnus® Amnion Patch Allografts<br><br>Cygnus® SOLO<br><br>Cygnus® DUAL<br><br>Cygnus® MATRIX<br><br>Cygnus® DISK<br><br>Cygnus® MAX	The Cygnus® Amnion Patch Allografts are designed in 6 configurations:<br>- SOLO: a traditional single-layer amniotic membrane graft<br>- DUAL: a dual-layered amniotic graft<br>- MATRIX: a flexible, multi-layer allograft comprised of the amnion layer, its intermediate/spongy layer and the chorion layers of the amniotic sac, providing improved handling and increased workability<br>- DISK: a multi-layer allograft available in circular shapes<br>- MAX: a more substantial natural thickness graft derived from the umbilical cord that can be sutured<br>E-Beam sterilization provides a sterility assurance level (SAL) of 10-6.<br>5-year shelf life	For use in advanced wound care, orthopedics, spine surgery, dermatology, ophthalmology, and oral surger	marketed	Vivex Biomedical	USA	USA	temporary (bioresorbable)	acellular	RT	dhAM / dhACM

Dermavest® Human Placental Connective tissue Matrix & Plurivest® Human Placental Connective tissue Matrix	Dermavest®: Typically presented as a particularized sheet that can be applied to wounds, and it is known to enhance collagen synthesis, promoting accelerated wound healing. Plurivest®: Functions as a placental-derived connective tissue matrix (CTM), which can be used to manage deep or cavity wounds, maintaining contact wit the wound bed without floating off. 4-year shelf life	Used for diabetic foot ulcers, venous ulcers, and various surgical, acute, or chronic wound applications. Both products act as a bridge for tissue repair, providing a structural matrix to facilitate healing in compromised tissue	marketed	Aedicell	USA	USA	temporary (bioresorbable)	acellular	RT	hPCTM
EpiCord®	EpiCord® is a dehydrated human umbilical cord (DHUC) allograft developed to provide a protective, biocompatible extracellular matrix (ECM) rich in hyaluronic acid, collagen, and over 250 regulatory proteins. 5-year shelf life	Designed to support the healing of chronic or complex wounds, this thick, malleable, and suturable membrane aids granulation tissue development, supports tissue repair, and is available in standard and expandable configurations. It is compatible with negative pressure wound therapy (NPWT) and hyperbaric oxygen therapy (HBOT). It should not be used in	Marketed	MiMedx Group	USA	USA	temporary (bioresorbable)	acellular	RT	dHUC

		areas with active or latent infection.								
Epifix®	Epifix® is composed of human amnion/chorion membrane (dHACM), processed to retain extracellular matrix proteins, growth factors, and cytokines.<br>5-year shelf life	Applied to non-infected, cleaned wounds to promote granulation tissue, often weekly or bi-weekly. Primarily used for chronic wounds such as diabetic foot ulcers (DFUs), venous leg ulcers, arterial ulcers, and pressure ulcers.	Marketed	MiMedx Group	USA	USA<br>Asia (Japan)<br>Australia<br>Middle East<br>Europe	temporary (bioresorbable)	acellular	RT	dhACM
FlowerAmnio Patch™ and<br><br>FlowerAmnio Flo™ (or Flower-Flo)	FlowerAmnioPatch™ is a dehydrated, dual-layer human amniotic membrane allograft intended as a "Ready-for-Surgery™" product to aid wound healing and tissue regeneration.<br>FlowerAmnioFlo™ is a flowable, acellular liquid amniotic fluid allograft.<br>5-year shelf life	FlowerAmnioPatch™ functions as a protective, natural covering that acts as an anti-adhesive barrier and minimizes inflammation and scar tissue formation.<br><br>FlowerAmnioFlo™ is intended to help regenerate soft tissue in non-healing wounds, including diabetic lower extremity ulcers, venous ulcers, and partial-to-full-thickness burns. It is also used in	marketed	Flower Orthopedics	USA	USA	temporary (bioresorbable)	acellular	RT	dhACM / hAMS

		orthopedic applications, such as joint or tendon treatment								
Genesis Amniotic Membrane	Genesis Amniotic Membrane is derived from human placental membrane donated by healthy mothers after elective cesarean section deliveries.<br>2-year shelf life	Applications for Genesis Amniotic Membrane include: Wound Care, Acute And Chronic Wounds, Burns, Cesarean Site Recovery, Lacerations, Skin Ulcers, Surgical Incisions, Laminectomy, Muscle Tears, Surgical Reconstruction, Tendon and Nerve Covering.	Marketed	Genesis Biologics	USA	USA	temporary (bioresorbable)	acellular	RT	hAM
Integra® BioFix® Amniotic Membrane Allograft	Integra® BioFix® is a sterile, dehydrated, and decellularized amniotic membrane allograft derived from human placental tissue: human amniotic membrane (BioFix) or amniotic/chorion tissue (BioFix Plus).<br>5-year shelf life.	It is used as a protective wound covering for surgical sites and tissue defects, aiding in inflammation modulation and tissue healing. Suitable for various wounds, including surgical, chronic, or, in the case of BioDOptix®, ocular surface defects. Can be applied on either side (omnidirectional), adheres	Marketed	Integra LifeSciences Corporation	USA	USA<br>Canada<br>Asia-Pacific region (Japan and China)	temporary (bioresorbable)	acellular	RT	dhAM / dhACM

		without fixation, and can be used wet or dry.								
Integra® BioFix® Flow Placental Tissue Matrix Allograft	The product is a, decellularized, flowable, particulate tissue matrix designed to be used as a wound covering or to fill tissue defects. It is supplied in a vial, which can be reconstituted with sterile saline to create a injectable material.<br>5-year shelf life.	Contains essential collagen, hyaluronic acid, fibronectin, laminins, and growth factors that support cellular proliferation, differentiation, and tissue repair. It addresses the management of complex wounds and surgical tissue defects, including: chronic wounds (diabetic foot ulcers, venous leg ulcers), deep or tunneling wounds, surgical soft tissue voids, tendinitis and other musculoskeletal applications.	Marketed	manufactured by Human Regenerative Technologies, LLC distributed by: Integra LifeSciences Corporation	USA	USA<br>Canada<br>Asia-Pacific region (Japan and China)	temporary (bioresorbable)	acellular	RT	hAMS
Interfyl™ Human Connective Tissue Matrix	It is a product derived exclusively from the chorionic plate of the placenta. The tissue is decellularized, minimally manipulated, and processed to remove cells, cellular material, and contamination while preserving the extracellular matrix (ECM)	Interfyl is used to fill voids, tunnels, and irregular spaces caused by trauma or surgery; support soft tissue augmentation during complicated closures, such as covering exposed bone, tendon, or ligaments; treat wounds,	Marketed	manufactured by Celularity Inc., following acquisition of Aliqua Biomedical in 2018 distributed	USA	USA<br>Middle East<br>North Africa	temporary (bioresorbable)	acellular	RT	dhPCTM

	components.<br>10-year shelf life	like diabetic foot ulcers, venous leg ulcers, and pressure wounds; offer soft tissue support such as damaged periarticular structures, including joint capsules. It is typically used when standard treatments have failed.		by Arthex, Versea Biologics and by the CH Trading Group to Middle East and the broader islamic world						
Neox® Wound Allografts<br>Neox® 1K<br>Neox® 100<br>Neox® RT	They are hydrated membranes, primarily composed of human amniotic membrane and umbilical cord tissue, not the chorion. They are, specifically, cryopreserved, thicker, and often described as containing amniotic membrane (AM) and/or umbilical cord (UC) matrix, focusing on heavy extracellular matrix (ECM) components.<br>Neox® (including 100 and 1K) is stored at -80°C to 4°C, which allows for storage in a standard freezer or refrigerator for up to 2 years. The product has a 6-hour room temperature stability, meaning it can be removed from cold storage for up to 6 hours	**Neox® 1K**: An "ultra-thick" cryopreserved allograft designed for deep or complex wounds.<br>**Neox® 100**: A thinner, cryopreserved allograft intended for shallow wounds, often provided on a gridded, non-implantable backing for easier handling.<br>**Neox® RT**: A room-temperature-stable, hydrated, and sterilized version of the umbilical cord/amniotic membrane, designed for easier storage without the need for specialized freezing.	marketed	Amniox Medical, Inc., a Biotissue company	USA	USA	temporary (bioresorbable)	acellular	cryo-preserved<br>fridge<br>RT	hAM<br>hUC

	and safely returned if the packaging remains unopened and intact. Unlike other cryopreserved products, Neox does not require special thawing equipment. There is a specific version called Neox® RT (Room Temperature) that is designed for ambient storage (20°C to 25°C).	Applications: diabetic foot ulcers (DFUs), venous and arterial ulcers, pressure ulcers, traumatic wounds and burns, surgical wounds and dehisced wounds .								
NuShield®	NuShield is an effective choice as a natural dehydrated human placental allograft that may be used to support wound management for a variety of partial- and full-thickness acute and chronic wounds, with a unique preservation method that analytical testing has shown retains all the native amnion and chorion layers with the spongy layer intact, unlike many placental allografts on the market.<br>5-year shelf life	NuShield® is an allograft intended for use in the management of acute and chronic wounds as well as appropriate surgical applications. NuShield® may be applied as a wound covering to a variety of partial- and full-thickness acute and chronic wounds and wounds with exposed tendon, muscle, joint capsule and bone. NuShield® can be applied from the onset and for the duration of the wound, weekly or at the discretion of the	Marketed	Organogenesis	USA	USA	temporary (bioresorbable)	acellular	RT	dhACM

		health care practitioner. It can't be applied on infected wounds.								
PalinGen® Membrane (dry)<br><br>PalinGen® Hydromembrane (hydrated)<br><br>PalinGen® X-Plus Membrane (crosslinked, dry)<br><br>PalinGen® X-Plus Hydromembrane (crosslinked, hydrated)	PalinGen® Membranes are human allograft wound coverings derived only from the amnion layer, specifically chorion-free. Using their proprietary Advantec® process, Amnio Technology produces these membranes without the chorion layer to avoid potential immunogenic reactions.<br>5-year shelf life	Wound coverings and support for native tissues. Just as the amniotic membrane acts as a physical barrier to cover and protect the fetus during pregnancy, PalinGen® Membranes provide a biological and physical overlay to support and protect the wound *in vivo*. Indicated for full and partial-thickness, acute, and chronic wounds.<br>PalinGen® X Plus Membranes usage:<br>Wound Management: Used for covering full and partial-thickness wounds, including venous ulcers, diabetic foot ulcers, burns, and post-surgical wounds.<br>Surgical Barrier (Anti-adhesion): Act as a	Marketed	Amnio Technology	USA	USA	temporary (bioresorbable)	acellular	RT fridge	dhAM / hAM

		physical barrier to prevent adhesions (scar tissue) and protect nerves, particularly in hernia repairs, tendon/ligament repairs, and neurosurgery. Orthopedic & Spinal Surgery: Used for repairing or augmenting tendons, fasciae, ligaments, and covering nerve bundles to reduce fibrosis. Dental Surgery: Applied as a protective dressing after procedures to manage tissue, such as in gum repair. Ocular Applications: Utilized to treat severe eye conditions, including corneal ulcers and chemical burns, as it is non-immunogenic.								
Restorigin™ Amniotic Tissue Patches	Restorigin amniotic membranes are procured through voluntary donation from scheduled cesarean procedures of full-term, live births. Restorigin is a dual layer amnion that offers the flexibility	Restorigin is recognized as a tissue product, often used within surgical, orthopedic, and wound care settings: chronic, non-healing wounds	Marketed	Parametrics Medical	USA	USA	temporary (bioresorbable)	acellular	RT	hAM

	of placing either side toward the wound, adheres naturally to the patient's tissue without sutures or other fixation.<br>5-year shelf-life	(diabetic ulcers, venous leg ulcers).								
Revita®	Revita® amniotic membrane is manufactured using the company's proprietary Clearify® technology, which processes full-thickness human placental membrane to create a full-thickness, lyophilized (freeze-dried) amniotic membrane, for use in wound care. It is a full-thickness, intact placental membrane containing all three layers: amnion, intermediate layer, and chorion.<br>5-year shelf life	It is commonly used for complex wounds, including diabetic, venous, and pressure ulcers.	Marketed	StimLabs (USA)<br>Surgical Biofix (Australia) – licenced by StimLabs	USA<br>Australia	USA<br>Australia	temporary (bioresorbable)	acellular	RT	dhCPTM
Salera® Membrane<br>Salera® Mini Membrane	Salera® Placental Membrane is minimally processed dehydrated amnion chorion and retains the structural properties of the extracellular matrix (ECM). The resulting dehydrated allograft serves as a wound covering.<br>3-year shelf life	This bi-layer placental allograft can aid in native tissueCrestoration and remodeling, while providing optimal-Ccoverage in a wide variety of sizes.	Marketed	MTF Biologics, a registered trademark of the Musculoskeletal Transplant Foundation	USA	USA	temporary (bioresorbable)	acellular	RT	dhACM

WoundEx® Membrane<br><br>WoundEx® Flow	WoundEx® Membrane is a dehydrated human amniotic/chorionic membrane allograft derived from human placental tissue.<br>5-year shelf life	Used for diabetic foot ulcers and venous leg ulcers to modulate inflammation and reduce scar tissue.	Marketed	Skye Biologics	USA	USA	temporary (bioresorbable)	acellular	cryo-preserved	dhACM / hAMS
Xwrap® Amniotic Membrane-Derived Allograft	XWRAP® is an amniotic, chorion-free membrane.	It is intended for homologous use as a protective barrier or cover applied to partial and full thickness acute and chronic wounds such as diabetic, venous, arterial, pressure and other ulcers, as well as traumatic and complex wounds, burns, surgical and Mohs surgery sites.	Marketed	Applied Biologics	USA	USA	temporary (bioresorbable)	acellular	RT	dhAM
I.3 Acellular dermal replacement from animal tissue source or algae										
Architect® stabilized collagen matrix	Decellularized equine pericardial tissue-derived dermal substitute<br>3-year shelf life	It is used for wound management and soft tissue repair (various chronic and acute wounds, including diabetic ulcers, surgical wounds, and burns).	Marketed	Harbor MedTech, Inc	USA	USA	temporary (biodegradable)	acellular	RT	
Biobrane®	Biosynthetic dressing made of a silicone film bonded to a nylon mesh and coated with	Biobrane® is a temporary, biosynthetic wound dressing used to cover clean partial-	Marketed	Smith & Nephew Medical Ltd., UK	UK	UK<br>USA<br>Australia	temporary (biodegradable)	acellular	RT	

	porcine Type I collagen<br>3-year shelf life	thickness burns and skin donor sites. It acts as a protective, artificial skin layer that stays in place for up to 14 days, or until the underlying wound heals and the dressing naturally lifts.		developed by: Woodroof Laboratories, USA						
Bio-ConneKt® Wound Matrix	Reconstituted collagen derived from equine tendon<br>6-year shelf life	Wound dressing for acute, chronic, traumatic, surgical, and burn wounds	marketed	MLM Biologics, Inc.	USA	USA	temporary (biodegradable)	acellular	RT	
CollaWound collagen sponge	Porcine collagen<br>2-year shelf life	It acts as a temporary matrix (scaffold) to facilitate wound healing, cell migration, and fluid absorption before being resorbed by the body.<br>These sponges are designed to be broken down, typically within 28 days, and absorbed into the tissue. Wound dressings.	Marketed	Collamatrix Co., Ltd.	Taiwan	Asia<br>Italy<br>Brazil<br>Malaysia<br>Argentina	temporary (biodegradable)	acellular	RT	

Cytal® wound matrix<br><br>Cytal® burn matrix<br><br>MicroMatrix® Flex<br><br>MicroMatrix® UBM Particulate	Porcine-derived urinary bladder matrix, non-cross-linked<br>Cytal® wound matrix: 2-year shelf life<br>Cytal® burn matrix: 3-year shelf life<br>MicroMatrix® Flex: 2-year shelf life, use 2h after preparation<br>MicroMatrix® UBM Particulate: 2-year shelf life	Cytal® wound matrix is used for managing complex, full-thickness wounds and aiding tissue regeneration. It acts as a temporary framework to support tissue remodeling and angiogenesis before being fully resorbed by the body. It is not a permanent implant. It is intended to be applied once, though it may require reapplication as it breaks down. During healing, it may form a gel-like substance as it is absorbed. Thicker versions of the device (e.g., 2-layer, 3-layer) may persist longer in the wound bed than thinner versions.<br>Cytal Burn Matrix is appropriate for the management of second degree burns.<br>MicroMatrix® Flex is a dual-syringe system designed to enable convenient mixing and	Marketed	Integra LifeSciences Corp, following acquisition of Acell, Inc. in 2021	USA	**GLOBAL:**<br>USA<br>Canada<br>China	temporary (bioresorbable)	acellular	RT	UBM

		delivery of MicroMatrix® UBM Particulate to hard-to-reach wound areas. MicroMatrix® UBM Particulate offers a wound management solution for irregular wounds. Applied as either a powder or paste, the particulate solution provides intimate contact with all areas of the wound bed.								
Endoform™ dermal template: Endoform™ Natural Endoform™ Antimicrobial	Ovine collagen (derived from ovine forestomach matrix) 2-year shelf life	Management of acute and chronic wounds, such as diabetic and venous ulcers (usually applied following soaking in saline. It is designed to be naturally incorporated into the wound bed over time, requiring reapplication every 5-7 days. It is not necessary to remove residual gel (broken-down material) during dressing changes, as this is part of the healing process.	Marketed	Manufacturer: Aroa Biosurgery Ltd. Originally distributed in the US by Hollister Wound Care (2013-2018), after 2018 distributed via Appulse Medical (Aroa/Hydrofera joint venture)	New Zealand	USA	temporary (biodegradable)	acellular	RT	

Excellagen®	Bovine-derived collagen wound matrix<br>It is a flowable gel (flowable fibrillar collagen matrix) supplied in prefilled single-use syringes (available in 0.1cc, 0.5cc, or 4.0cc configurations) for easy application.<br>1-2 years shelf life	It is designed for treating hard-to-heal wounds such as diabetic foot ulcers, partial and full-thickness wounds, and pressure ulcers. It is designed to be used in conjunction with standard wound care.	Marketed	Manufacturer: Oleregen Therapeutix, Inc.<br>Former manufacturers: Gene Biotherapeutics, Taxus Cardium Pharmaceuticals Group<br>Manufacturing Partner: Collagen Solutions PLC	USA	USA<br>Germany<br>Switzerland<br>South Korea	temporary (biodegradable)	acellular	fridge	
EZ Derm®	Dehydrated porcine dermis (xenograft), 41 ompany 41 -treated<br>2-year shelf life	Used in partial-thickness burns, skin loss injuries, and as a covering for wounds. It acts as a protective barrier and is designed to adhere to the wound, eventually separating from the underlying skin as it heals.	Marketed	Mölnlycke Health Care	USA	USA<br>Europe	temporary (biodegradable)	acellular	RT	
Geistlich Derma-Gide™	Porcine -derived advanced wound care matrix<br>3-year shelf life	It acts as a scaffold for tissue ingrowth, promoting regeneration. It is applied to cleaned, debrided wounds and	Marketed	Manufacturer: Geistlich Pharma North America Inc.	Switzerland	USA	temporary (biodegradable)	acellular	RT	

		it is designed to break down as the body repairs the wound. It is used for various wounds, including diabetic ulcers, venous ulcers, and surgical wounds. It is not indicated for third-degree burns or in patients with known allergies to collagen or porcine materials.		Exclusive US distributor: StimLabs						
Helicoll™	Bovine collagen (bioengineered high purity Type-I collagen: >97% pure)<br>3-year shelf life	It is a highly bioactive and cell conducive acellular skin substitute construct. It enhances tissue generation for wound management. Treatment course typically involves 1 to 4 applications.	Marketed	Manufacturer: EnColl Corporation Exclusive Distributor: Kadiri Health	USA	USA	temporary (biodegradable)	acellular	RT	
Integra® Matrix Wound Dressing = Integra® Wound Matrix (originally Avagen wound dressing)	Cross-linked bovine tendon collagen and glycosaminoglycan (wound care device, a biodegradable porous matrix which works like a scaffold for cellular invasion and capillary growth)<br>2-year shelf life.	It provides coverage over exposed bone, tendon, cartilage, and joints. Integra Matrix Wound Dressing is indicated for the management of wounds including: partial and	Marketed	Integra LifeSciences Corp.	USA	**GLOBAL**	temporary (biodegradable)	acellular	RT	

		full-thickness wounds, pressure ulcers, venous ulcers, diabetic ulcers, chronic vascular ulcers, tunneled/undermined wounds, surgical wounds (donor sites/grafts, post-Moh's surgery, post-laser surgery, podiatric, wound dehiscence), trauma wounds (abrasions, lacerations, second-degree burns, skin tears) and draining wounds.								
Integra Flowable Wound Matrix	Granulated cross-linked bovine tendon collagen and glycosaminoglycan<br><br>Integra® Flowable Wound Matrix is a transformative wound care product that utilizes the Integra® technology in a flowable delivery system. It offers a 2-syringe mixing and delivery system. The product syringe contains granulated particulate of ultra-pure bovine collagen and chondroitin-6-sulfate matrix specifically engineered with optimized pore size,	When mixed with sterile saline, Integra Flowable can be applied to difficult to access wound sites and complex anatomical geometries, such as undermined or tunneled wounds. Integra Flowable Wound Matrix is indicated for the treatment of tunneling and/or undermined wounds including surgical wounds (donor sites/grafts, post-Moh's surgery, post-laser	marketed	Integra LifeSciences Corp.	USA	**GLOBAL**	temporary (bioresorbable)	acellular	RT	

	porosity, and structure to allow for cells to infiltrate, proliferate, and aid in the healing process.<br>18 months shelf life	surgery, podiatric, wound dehiscence) and diabetic ulcers of both partial and fullthickness varieties								
Kerecis® Omega3 Wound	A sterile, omega-3 rich collagen scaffold (acellular dermal matrix) derived from North Atlantic cod skin, that retains lipids and proteins to support tissue regeneration, used as wound dressing<br>3-5 years shelf life	Used for partial/full-thickness wounds, diabetic foot ulcers, burns, and post-Mohs surgery repairs	marketed	Coloplast A/S, following acquisition of Kerecis in 2023	Iceland	USA<br>Europe<br>Southeast Asia	temporary (biodegradable)	acellular	RT	FSG
Matriderm®	A bovine-derived acellular matrix composed of collagen and elastin.<br>It is a dry, collagen-elastin matrix that is shelf-stable and requires rehydration in sterile saline or Ringer's solution immediately before application to the wound bed.<br>5 years shelf life	Used in burn, trauma, chronic wound management, for reconstruction following cancer excision and other types of reconstructive surgery (in combination with split-thickness skin grafts).	Marketed	manufacturer: MedSkin Solutions Dr. Suwelack<br>distributor US: Access Pro Medical<br>distributor India: GRH	Germany	**GLOBAL:**<br>Europe<br>Middle East<br>Asia<br>US<br>Latin America	temporary (bioresorbable)	acellular	RT	
MicroMatrix® Flex	Porcine-derived extracellular matrix (porcine urinary bladder matrix)<br>2-3 years shelf life	MicroMatrix® can be applied as a powder or mixed with saline to form a paste. MicroMatrix® Flex is a specific, easy-to-mix, dual-	Marketed	Integra LifeSciences Corp, following acquisition of Acell, Inc. in 2021	USA	USA<br>China, inckuding Hong Kong<br>South Korea	temporary (biodegradable)	acellular	RT	UBM

		syringe system designed for this purpose, featuring a flexible tip to reach deep or irregular wound areas (dental, irregular wound bed, wounds against gravity: foot/sacral region, deep wounds, wounds with tunneling/undermining). The paste should be used within 2 hours of preparation. A non-adherent dressing is typically placed over the application, and the resulting caramel-colored gel (a normal sign of hydration) should be left on the wound during dressing changes. Indicated for partial and full-thickness wounds, including chronic ulcers (diabetic, venous, pressure), surgical sites, and trauma wounds (abrasions, burns, lacerations). UBM technology helps shift the								

		wound environment from inflammatory to one that facilitates healing and remodeling.								
Miroderm® Fenestrated MiroDerm® Fenestrated Plus	Biologic wound matrix derived from porcine liver 3-year shelf life	MiroDerm® Biologic Wound Matrix is indicated for the management of wounds including partial- and full-thickness wounds; pressure injuries; venous ulcers; chronic vascular ulcers; diabetic ulcers; tunneled, undermined wounds; trauma wounds (abrasions, lacerations, second-degree burns, skin tears); draining wounds; and surgical wounds (donor sites/grafts, post-Mohs surgery, post-laser surgery, podiatric, wound dehiscence).	Marketed	Reprise Biomedical, spinned off from Miromatrix Medical, Inc. in 2019	USA	USA	temporary (bioresorbable)	acellular	RT	
NovoMaix	Acellular porcine collagen-sponge scaffold, received Conformité Européenne (CE) mark certification in 2013. long-term shelf life, but there	Designed for use with autologous skin graft, for "one-step" surgical interventions. It is used to treat full-thickness wounds and serves as a	Ended clinical trials received CE	manufactured by: Matricel GmbH developed by:	Germany	EU EEA	temporary (bioresorbable)	acellular	RT	

	are no details about current production for sale.	base for split-thickness skin grafts.	? marketed	EuroSkinGraft Consortium						
Oasis® Wound Matrix	An acellular matrix derived from porcine small intestinal submucosa<br>2-year shelf life	Management of various wound types	marketed	Smith & Nephew, Inc.	USA	USA	temporary (biodegradable)	acellular	RT	
Ologen™ Collagen Matrix	Porcine type I atelocollagen (>90%) and glycosaminoglycans (<10%)<br>3-year shelf life	Wound scaffold for ophthalmological, reconstructive, and general surgeries<br>Distributed for use in over 100,000 glaucoma surgeries worldwide. While it usually degrades within 180 days, some studies have noted its presence for up to 1 year, but it is fundamentally designed to be replaced.	Marketed	Aeon Astron Corporation	Taiwan	**GLOBAL:**<br>43 countries<br>USA<br>Europe<br>Asia	temporary (biodegradable)	acellular	RT	
Permacol™ surgical implant	Porcine dermal collagen implant (3D collagen matrix cross-linked for enhanced durability)<br>3-year shelf life	This biologic mesh is designed for hernia repair and soft tissue reconstruction, such as wall hernia repair.	Marketed	Sofradim Production, a subsidiary of Covidien, which is part of Medtronic	France	Europe<br>Asia<br>USA<br>Canada	temporary (biodegradable)	acellular	RT	

PriMatrix®<br>PriMatrix Ag (antimicrobial with ionic silver)<br>Dermal Repair Scaffolds	Acellular dermal tissue matrix derived from fetal bovine (calf) dermis.<br>5-year shelf life	Partial and full thickness wounds, pressure, diabetic, and venous ulcers, second-degree burns, surgical wounds—donor sites/grafts, post-Moh's surgery, post-laser surgery, podiatric, wound dehiscence, trauma wounds —abrasions, lacerations, and skin tears, tunneled/undermined wounds and raining wounds	marketed	Integra LifeSciences Corp., following acquisition of TEI Biosciences/TEI Medical in 2015	USA	USA<br>Europe<br>Middle East<br>Africa	temporary (biodegradable)	acellular	RT	
Puracol®<br>Puracol® Plus<br>Puracol® Plus Ag+<br>Puracol® Ultra Powder<br>Collagen Wound Dressings	Bovine collagen (type I 100% native collagen)<br>2-year shelf life	It is a biodegradable, native collagen dressing that is replaced every 1 to 7 days depending on the level of wound exudate. It acts as a scaffold to jump-start healing in stalled, non-healing wounds.<br>Puracol Plus Ag+ inhibits bacterial growth<br>Puracol Ultra Powder conforms to irregularly shaped wound sites	marketed	Medline Industries	USA	USA	temporary (biodegradable)	acellular	RT	

PuraPly® Anti-microbial Wound Matrix (PuraPly® AM)<br>PuraPly XT (cross-linked)	Porcine intestinal collagen<br>5-year shelf life (frozen)	They are used to manage the wound environment, disrupt biofilm, and promote healing, often for chronic wounds. They are indicated for the management of: partial- and full-thickness wounds, pressure ulcers, venous ulcers, diabetic ulcers, chronic vascular ulcers, tunneled/undermined wounds, surgical wounds (donor sites/grafts, post-Mohs surgery, post-laser surgery, podiatric, wound dehiscence), trauma wounds (abrasions, lacerations, second-degree burns, skin tears) and draining wounds.	Marketed	Organogenesis, Inc.	USA	USA	temporary (biodegradable)	acellular	RT/ cryo-preserved	
Talymed®	Fibers of poly-N-acetyl glucosamine isolated from microalgae<br>3-year shelf life	Talymed is indicated for the management of a range of serious, complex wounds including: diabetic ulcers, venous ulcers, pressure wounds, dehisced surgical wounds, ulcers caused by mixed	marketed	Marine Polymer Technologies, Inc.	USA	USA	temporary (biodegradable)	acellular	RT	

		vascular etiologies, full-thickness and partial-thickness wounds, second degree burns, surgical wounds/donor sites/grafts, post-Mohs surgery, post-laser surgery, abrasions, lacerations, traumatic wounds healing by secondary intention, chronic vascular ulcers								
TheraForm™ Standard/Sheet Absorbable Collagen Membrane	Porcine collagen absorbable wound 50ompany50<br>5-year shelf life	It is designed for wound management, specifically to aid healing in chronic, non-infected partial and full-thickness wounds. It functions as a wound covering that helps manage exudate, reduces scar tissue, and promotes healing in venous stasis, diabetic, and surgical wounds, or as a soft tissue barrier	marketed	Sewon Cellontech Co.	Republik of Korea	USA	temporary (bioresorbable)	acellular	RT	

I.4 Acellular dermal replacement from synthetic materials										
Hyalofast®	HYALOFAST® is composed of a single 3D fibrous layer of HYAFF®, a benzyl ester of hyaluronic acid (HA), which is a natural component of the extracellular matrix and a major component of human cartilage. HA from bacterial fermentation, non-animal-based product.<br>3-4 years shelf life	Cartilage regeneration: once implanted, its non-woven 3D structure supports MSC adhesion and 3D organization, facilitating the recovery of the normal tissue anatomy. As the HYAFF fibres degrade, HA is released into the lesion creating a micro-environment favorable to regeneration. It is effective for chondral and osteo-chondral defects; it can be used both as scaffold for bone marrow aspirate or chondroprotective coverage after bone marrow stimulation procedures such as NanoFx®. It is easily applied in mini-arthrotomy or in arthroscopy and it can be applied on either side. It is resorbed following the natural pathway of endogenous hyaluronic acid.	Marketed	Anika Therapeutics, Inc. (USA), following acquisition of Fidia Advanced Biopolymers (Italy) in 2009	Italy	**GLOBAL:**<br>USA<br>Europe<br>Asia<br>Middle East<br>Latin America	temporary (biodegradable)	acellular	RT	

Hyalomatrix® (in ths USA)<br>Hyalomatrix® (Non-Silicone or NS) (in the U.S.)<br>Hyalomatrix® PA (in other international markets)	It is a bilayered wound dressing composed of a nonwoven pad made of HYAFF® (a long-lasting benzyl ester of hyaluronic acid) ± a semipermeable silicone membrane (found only in the original product), so it is a derivatized hyaluronic acid scaffold. Upon wound contact, HYAFF® transforms into a hydrophilic gel and integrates with the surrounding tissue. It is a tissue reconstruction matrix (TRM)<br>2-4 years shelf life	Hyalomatrix® usages: Partial and full-thickness wounds, Second-degree burns, Pressure ulcers, Venous ulcers, Diabetic ulcers, Chronic vascular ulcers<br><br>Hyalomatrix® NS usages: Tunneled/undetermined wounds, Surgical wounds (donor sites/grafts, post-Mohs surgery, post-laser surgery, podiatric, wound dehiscence), Trauma wounds (abrasions, lacerations, skin tears), Draining wounds	marketed	current manufacturer: Anika Therapeutics, Inc. (USA)<br>first manufacturer: Fidia Advanced Biopolymers (Italy)<br>current distributors in the US: Medline Industries, Inc. (main), Misonix, Inc.<br>online supliers in the US: Pipeline Medical, Grayline Medical, WoundCareShop.com, Tabeek Medical Supply, Total Care Medical Supply	USA	Europe<br>USA<br>Latin America	permanent (integrating): the HYAFF component<br><br>temporary (removable): the silicone layer	acellular	RT	

NovoSorb® Biodegradable Temporising Matrix (BTM)	It is comprised of:<br>1. A sealing membrane (a temporary non-biodegradable layer that closes the wound, limiting moisture loss while also serving as a barrier to outside bacteria)<br>2. Adhesive between the sealing membrane and the buodegradable matrix<br>3. A synthetic, biodegradable and biocompatible polyurethane foam matrix (open-cell)<br>3-year shelf life	It is primarily used for treating complex burns, surgical wounds, and deep traumatic wounds. BTM acts as a synthetic, bioresorbable dermal scaffold to regenerate the dermis in deep, complex wounds.<br>When it is fully integrated (after 2-3 weeks), the sealing membrane is removed, leaving a fully vascularized dermis, ready for definitive closure (secondary treatment). BTM progressively biodegrades and is fully absorbed in approximately 18 months	marketed	manufacturer: PolyNovo Biomaterials Pty Ltd developed at the Royal Adelaide Hospital	Australia	**GLOBAL:**<br>USA<br>Australia<br>New Zealand<br>South Africa<br>Europe<br>Middle East<br>Asia	temporary (removable): sealing membrane<br><br>temporary (bioresorbable): the polyurethane matrix	acellular	RT	
Restrata™	It is a sterile, synthetic, electrospun and bioresorbable wound matrix (polyglactin 910 and polydioxanone) produced using a proprietary electrospinning process to create nanofabricated scaffolds.<br>2-year shelf life	It is utilized by surgeons to support tissue regeneration in complex wounds, trauma cases, and plastic/reconstructive surgeries as it provides temporary, structural, synthetic scaffolding that mimics the	Marketed	Solventum (aquired previous manufacturer, Acera Surgical, in Nov 2025)	USA	USA	temporary (bioresorbable)	acellular	RT	

		extracellular matrix (ECM) before fully resorbing.								
I.5 Acellular epidermal and dermal replacement from combined natural and synthetic materials										
Integra Bilayer Matrix Wound Dressing (BWM)	A semi-synthetic bilayer consisting of a dermal-like layer (porous matrix of cross-linked bovine tendon collagen and glycosaminoglycan) and an epidermal-like layer (synthetic polysiloxane polymer).<br>2-year shelf life	Bilaminar membrane that acts as a scaffold for cellular invasion and capillary growth (new dermal tissue growth = neodermis) in the treatment of diabetic foot ulcers, complex burns, and reconstruction following trauma or surgery.	Marketed	Integra LifeSciences Corp.	USA: originally; manufacturing facilities worldwide	**GLOBAL:**<br>Europe<br>Asia<br>Australia<br>US<br>Latin America	permanent (integrating): collagen (dermal) layer<br><br>temporary (removable): silicone (epidermal) layer	acellular	RT	BWM
Integra Dermal Regeneration Template (IDRT)	Composed of cross-linked type I collagen (bovine) and chondroitin-6-sulfate (shark): dermal substitute, plus a silicone layer (epidermal substitute).<br>2-year shelf life	Used to treat deep-partial to full-thickness burns and in reconstructive surgery. The first and only FDA-approved skin substitute for the treatment of third degree burns and scar contracture proven to regenerate dermis. It is indicated for: the post-excisional treatment of life-threatening full-thickness or deep partial-thickness thermal injuries where	Marketed	Integra LifeSciences Corp.	USA: originally; manufacturing facilities worldwide	USA	permanent (integrating): collagen (dermal) layer<br><br>temporary (removable): silicone (epidermal) layer	acellular	RT	DRT

		sufficient autograft is not available at the time of excision or not desirable due to the physiological condition of the patient; repair of scar contractures when other therapies have failed or when donor sites for repair are not sufficient or desirable due to the physiological condition of the patient; and treatment of partial and full-thickness neuropathic diabetic foot ulcers that are greater than six weeks in duration with no capsule, tendon or bone exposed, when used in conjunction with standard diabetic ulcer care.								
Omnigraft® Dermal Regeneration Matrix (DRM)	Omnigraft® Dermal Regeneration Matrix is an advanced bilayer (two-layer) matrix for dermal regeneration. Integra's exclusive bilayer technology offers a novel bioengineered	Integra® Omnigraft Dermal Regeneration Matrix is indicated for use<br>in the treatment of partial and full-thickness neuropathic diabetic	Marketed	Integra LifeSciences Corp	USA	USA	permanent (integrating): collagen (dermal) layer<br><br>temporary (removable):	acellular	RT	DRM

	collagen and chondroitin-6-sulfate matrix combined with a protective silicone outer layer.<br>3-year shelf life	foot ulcers that are greater than six weeks in duration, with no capsule, tendon or bone exposed, when used in conjunction with<br>standard diabetic ulcer care.					silicone (epidermal) layer			
I.6 Acellular epidermal and dermal replacement from human sources										
AltiPly® (placental membrane)<br>AltiPly® Lite (amniotic membrane)	AltiPly® is a structural, multi-layer, opaque, amniotic membrane allograft, designed as a temporary wound covering to support healing in acute or chronic wounds. It is a growth factor-rich matrix, with an outer basement membrane and epithelial layer, that supports re-epithelialization.<br>5-year shelf life	Next generation allograft that offers easy-to-use wound care solutions: natural physiological barrier for various applications, optimal preservation of endogenous growth factors, supports wound closure of chronic diabetic foot ulcers, venous leg ulcers and pressure wounds, enables rapid, non-pathologic healing and remodeling of surgical and reconstructive wounds.	Marketed	Berkeley Biologics LLC (a subsidiary of GNI Group), following acquisition of the orthobiologics business unit from Elutia (formerly Aziyo Biologics) in 2023	USA	USA	temporary (biodegradable)	acellular	RT	dhACM / dhAM

GammaGraft™	GammaGraft is a gamma-irradiated temporary human skin allograft storable at room temperature, used to cover wounds, such as burns and chronic ulcers, acting as a bridge to promote healing or to provide temporary covering before a permanent, autologous (patient's own) skin graft can be applied. It includes an epidermal and a dermal layer. It can be removed if necessary, or it may naturally peel off as the wound heals.<br>2-year shelf life	Used as a temporary skin graft on burns and chronic wounds (Venous Stasis Ulcers, Diabetic Foot Ulcers, Full Thickness Ulcers, Mohs Surgery Sites, Skin Graft Donor Sites, Areas of Dermabrasion, Partial Thickness Burns, Partial Thickness Wounds, Temporary Coverage for Exposed Abdominal Viscera including Small Bowel and Liver, Exposed Pericranium and Cranium, Fasciotomy Sites, Areas of Excision which are not closed pending final pathology report). GammaGraft can be used without any other wound coverings after the GammaGraft has dried on the wound. This is possible because GammaGraft has a natural keratin layer that acts as a vapor barrier for the wound. This	Marketed	Promethean LifeSciences	USA	USA	temporary (removable)	acellular	RT	hADM

		allows for moist wound healing and a moist wound bed, even though the GammaGraft is dry.								
**II. Cellular skin substituents**										
II.1 Cellular dermal replacement from donated human skin										
AlloSkin™ RT	AlloSkin RT meshed human dermal graft is a sterile skin graft (electron beam sterilized).<br>2-year shelf life	For homologous use on any skin defect and is appropriate for use in traumatic and chronic wounds, including those where substructures such as bone, ligament, nerve or muscle are exposed. It is used as a biological dressing for both acute and chronic wounds, including deep, full-thickness, or partial-thickness wounds.	Marketed	AlloSource	USA	USA	temporary (removable)	cellular	RT	allograft
AlloSkin™ split-thickness dermis	AlloSkin is a meshed human dermal allograft<br>5-year shelf life	For clinical treatment of acute, traumatic/surgical and chronic wounds, including diabetic foot ulcers and venous wound ulcers. It provides a biologic protective cover, that allows the patient's	Marketed	AlloSource	USA	USA	temporary (removable)	cellular	cryo-preserved	allograft

		body to successfully heal. It is also useful for critical burn applications prior to autografting to ensure the wound bed is adequately prepared for successful closure.								
PureSkin™ split-thickness dermis PureSkin™ XL split-thickness dermis	PureSkin, human skin allografts, are temporary wound dressings containing cells and biologic factors native to human skin.<br>Optimized shelf life through cryopreservation	They are used with critical burn patients to aid in infection control, fluid maintenance and pain control, as well as to conserve the use of precious autograft skin.<br>The XL variant sizes up to 1800cm2 and greater, for large TBSA burns and for other critical dermal conditions, such as necrotizing fasciitis. Meshing of 2:1 allows exudate management, flexibility or diverse anatomical coverage and maximum expansion, if needed.	Marketed	AlloSource	USA	USA	temporary (removable)	cellular	cryo-preserved	allograft

II.2 Cellular dermal replacement from human placental membrane										
Affinity® Human Amniotic Allograft	It is a fresh, hypothermically stored amniotic membrane (maintained under refrigeration, specifically between 1 °C and 10 °C).<br>+2 years shelf life	It is used as a regenerative, living-cell scaffold for chronic wound management	marketed	manufacturer: Organogenesis, Inc. processor: LifeLink® Tissue Bank (AATB accredited and FDA registered)	USA	USA	permanent (integrating)	cellular	fridge	
FlōGraft® Amniotic Fluid-Derived Allograft	FlōGraft® is a cryo-preserved, injectable amniotic fluid-derived allograft. It is a processed, flowable tissue product designed to maintain the integrity of the extracellular matrix and growth factors.<br>5-year shelf life	It is used to treat soft tissue pain, inflammation, and injury, particularly for enhancing repair, reconstruction, and regeneration in orthopedics, sports medicine, and wound care. It is frequently applied to address osteoarthritis, tendonitis, ligament tears, and chronic wounds.	Marketed	Applied Biologics	USA	**GLOBAL:**<br>USA<br>Canada<br>Mexico<br>Europe<br>Asia-Pacific<br>Brazil<br>South Africa<br>Saudi Arabia	temporary (bioresorbable)	cellular	cryo-preserved	

Grafix® Prime / Core (crypreserved) Grafix® PL (lyopreserved)	Grafix placental membranes are advanced, cryopreserved (or lyopreserved = RT) human tissue allografts derived from amniotic membrane These flexible, conforming covers contain live, endogenous cells (such as mesenchymal stem cells and fibroblasts) and growth factors.<br>2-3 years shelf life for the cryopreserved product<br>2-year shelf life for the RT product	Grafix® placental membranes are used as a covering for chronic and acute wounds, including diabetic foot ulcers (DFUs), venous leg ulcers, pressure injuries, and surgical wounds. They are applied directly to wounds to promote healing by providing a scaffold for cellular repair, reducing inflammation, and covering exposed bone or tendon.	Marketed	Smith & Nephew, Inc., following acquisition of Osiris Therapeutics, Inc. in 2019	USA	USA	temporary (bioresorbable)	cellular	cryo-preserved / RT	
II.3 Cellular dermal replacement from combined natural and synthetic materials										
Dermagraft®	Dermagraft® is a cryo-preserved human fibroblast-derived dermal substitute. It must be thawed prior to application. It contains living fibroblasts, extracellular matrix, and a bioabsorbable scaffold (allo hF on poly-galactin mesh).	Full-thickness diabetic foot ulcers	marketed	Organogenesis, Inc.	USA	USA<br>Canada<br>UK	temporary (bioresorbable)	cellular	cryo-preserved	

II.4 Cellular epidermal and dermal replacement from donated human dermis or autologous skin sample										
EpiCel®	Cultured epidermal autograft multi-layer sheet (cultured auto hK multi-layer sheet)<br>Unlike temporary dressings or allografts (donor skin), EpiCel is engineered to permanently replace the outer layer of skin. It uses the patient's own skin cells, grown in an incubator, which reduces the risk of rejection.<br>24h from 62ompany62 shelf life	Deep dermal or full-thickness burns	marketed	Vericel Corporation	USA	USA	permanent (integrating)	cellular	cool RT (13-23 ^{o}C)	auto hK
ReCell®	Uncultured suspension of auto hK, delivered as a spray;<br>It is a medical device that enables healthcare professionals to create a regenerative epithelial suspension (Spray-On Skin™ Cells) from a patient's own skin cells at the point of care. A small, thin sample of the patient's own skin is taken and processed within the device to create a suspension of skin cells (keratinocytes and melanocytes). This mixture is then	Partial-thickness burns & trauma wounds<br>Vitiligo/pigmentation	marketed	AVITA Medical	USA	United States<br>Europe<br>Australia<br>Japan<br>China	permanent (integrating)	cellular	controlled RT (max 27°C for all components)	auto hK

	sprayed or dripped directly onto the wound, covering a much larger area than the original donor sample.<br>21 months shelf-life									
SkinTE™	SkinTE™ is designed as a regenerative, autologous (patient-derived) cellular therapy aimed at creating permanent, functional skin rather than serving as a temporary covering. SkinTE is an investigational, autologous heterogeneous skin construct designed to initiate the wound healing process by providing healthy cells and chemokines in the wound bed. Following application, multicellular segments may engraft and serve as a cellular source within the wound bed. In clinical studies, SkinTE has demonstrated the ability to initiate cellular activity associated with tissue repair and re-pigmenting, although it is	It is used for the regeneration of full-thickness skin, particularly for chronic wound healing and diabetic foot ulcers.<br>It is primarily approved and marketed for use in the U.S. for treating complex wounds, including chronic wounds and burns. It has received FDA breakthrough therapy designation for diabetic foot ulcers and is used across various U.S. medical facilities. The product is in advanced stages of clinical development, with Phase III trials	Clinical trials<br><br>registered as a 361 human cell, tissue, or cellular or tissue-based product (HCT/P), in transition to a 351 BLA (Biologics License Application) for specific	PolarityTE	USA	USA	permanent (integrating)	cellular	controlled RT<br>cool RT according to the clinical protocol	autograft

	positioned for regeneration.<br>Yet to be defined shelf-life	completed for diabetic foot ulcers. While research and trials are focused on U.S. applications, SkinTE is designed for broad application in regenerative skin therapy.	chronic wounds							
TheraSkin®	TheraSkin is an all-human, split-thickness skin allograft with living cells and retained endogenous growth factors, and a native extracellular matrix. TheraSkin can be vascularized by the recipient following transplantation to support development of granulation tissue, which aids in epithelialization to support wound closure.<br>5-year shelf life	It is used to treat chronic wounds like diabetic foot ulcers (DFUs), venous leg ulcers (VLUs), and pressure sores. It contains living fibroblasts and keratinocytes that promote healing and can be applied over exposed bone, tendon, or muscle. The white, collagen-rich dermal side is placed directly onto the wound, typically every 2 weeks. The graft is usually covered with a non-adherent, moist-regulating dressing (e.g., TheraGauze).	Marketed	LifeNet Health Biologics	USA	USA	temporary (biodegradable)	cellular	cryo-preserved	autograft

II.5 Cellular epidermal and dermal replacement from combined human and animal sources										
Apligraf®	It is a living, bi-layered skin substitute composed of human neonatal foreskin-derived cells (keratinocytes and fibroblasts) and bovine type 1 collagen (allo hF collagen gel plus stratified to hK)<br>15-day shelf life from the time of packaging	It is used for treating chronic venous leg ulcers and diabetic foot ulcers.	Marketed	Manufacturer: Organogenesis, Inc. Distributor: Novartis has been involved in distribution	USA	USA<br>Canada<br>Mexico<br>Singapore<br>South Africa<br>Saudi Arabia<br>Kuwait	temporary (biodegradable)	cellular	cool RT (20-23 ^{o}C)	
StrataGraft®	StrataGraft® is an allogeneic cellularized scaffold product containing metabolically active cells that produce and secrete a variety of growth factors and cytokines (allogeneic cultured keratinocytes and dermal fibroblasts in murine collagen-dsat = allo hF collagen gel plus stratified to hK)<br>1 year (cryopreserved) and 8 days (freezed) shelf life	It is approved for treating deep partial-thickness burns and other complex skin defects. It provides a durable closure of the wound in the majority of patients without requiring further surgical procedures.<br>StrataGraft® is considered a temporary bioengineered skin substitute, designed to act as a scaffold that supports the body's own healing process and is eventually replaced by the patient's own skin cells, often reducing or eliminating the need for	Marketed	Stratatech Corporation, a Mallinckrodt 65 ompany utilizing facilities on the University of Wisconsin-Madison campus for manufacturing under FDA-mandated Good Manufacturing Practices (GMP)	USA	USA	temporary (biodegradable)	cellular	cryo-preserved	

		autografting (taking skin from another part of the body). It does not remain permanently engrafted; it is gradually replaced by the patient's own cells, with no DNA from the graft detected after 3 months.								
References [16–71]										

References

1. SYNYO GmbH. BIOMATDB [Internet]. [cited 2026 Apr 13]. Available from: https://www.biomatdb.eu/
2. HEALTH CLUST-ER. Associazione Clust-ER Industrie della Salute e del Benessere [Internet]. [cited 2026 Apr 13]. BIOMATDB Project . Available from: https://health.clust-er.it/en/biomatdb-2/
3. Tarliz L, Kumbhar S, Passaro E, Yakutovich A, Granata V, Gargiulo F, Borelli M, Uhrin M, Huber SP, Zoupanos S, Adorf CS, Andersen CW, Schutt O, Pignedoli CA, Passerone D, VandeVondele J, Schulthess TC, Smit B, Pizzi P, Marzari N. Materials Cloud, a platform for open computational science. Nature - Scientific Data [Internet]. 2020 [cited 2025 May 25]. Available from: https://www.nature.com/articles/s41597-020-00637-5
4. Open Source - Github. DEBBIE Project [Internet]. [cited 2026 Apr 13]. Available from: https://projectdebbie.github.io/
5. NIMS Japan. MatNavi [Internet]. 2019 [cited 2026 Apr 13]. Available from: https://mits.nims.go.jp/
6. National Institute of Standards and Technology U. Genome Materials Initiative [Internet]. 2011 [cited 2026 Apr 13]. Available from: www.mgi.gov
7. Swiss National Science Foundation and European Commission. Cloud Materials [Internet]. 2020 [cited 2026 Apr 13]. Available from: https://www.materi-alscloud.org/
8. The Materials Project [Internet]. 2021 [cited 2026 Apr 13]. Available from: https://next-gen.materialsproject.org
9. MatWeb LLC. MatWeb [Internet]. 2011 [cited 2026 Apr 13]. Available from: https://www.matweb.com/
10. Open Source. NOMAD database [Internet]. 2014 [cited 2026 Apr 13]. Available from: https://nomad-lab.eu/nomad-lab/
11. NIMS Japan. DICE [Internet]. 2019 [cited 2025 May 25]. DICE. Available from: https://dice.nims.go.jp/link.html
12. NIMS Japan. PoLyInfo [Internet]. [cited 2025 May 25]. Available from: https://polymer.nims.go.jp/
13. Huber SP, Zoupanos S, Uhrin M, Talirz L, Kahle L, Häuselmann R, Gresch D, Müller T, Yakutovich A V., Andersen CW, Ramirez FF, Adorf CS, Gargiulo F, Kumbhar S, Passaro E, Johnston C, Merkys A, Cepellotti A, Mounet N, Marzari N, Kozinsky B, Pizzi G. AiiDA 1.0, a scalable computational infrastructure for automated reproducible workflows and data provenance. Sci Data. 2020 Dec 1;7(1). doi:10.1038/s41597-020-00638-4 PubMed PMID: 32901044.
14. Sewell C, Pizzi G, Talirz L. NCCR Marvel Switzerland [Internet]. 2020 [cited 2025 May 25]. Quanum Mobile (virtual machine). Available from: https://quantum-mobile.readthedocs.io/en/latest/
15. NCCR Switzerland. NCCR Switzerland [Internet]. 2020 [cited 2025 May 25]. MARVEL project. Available from: https://nccr-marvel.ch/project/about
16. Integra LifeSciences Corporation. Integra products [Internet]. 2026 [cited 2026 Mar 2]. Available from: https://products.integralife.com/engineered-colla-gen/category/wound-reconstruction-engineered-collagen
17. StimLabs LLC. StimLabs products [Internet]. 2026 [cited 2026 Mar 2]. Available from: https://stimlabs.com/
18. Musculoscheletal Transplant Foundation. mtfbiologics products [Internet]. 2025 [cited 2026 Mar 2]. Available from: https://www.mtfbiologics.org/who-we-serve/healthcare-professionals/wound-care-professionals
19. AlloSource. AlloSource Products [Internet]. 2022 [cited 2026 Mar 2]. Available from: https://allosource.org/our-products/?tax_tissue=dermis&unfilter=1
20. Polarity Bio. SkinTE [Internet]. 2026 [cited 2026 Mar 2]. Available from: https://www.polaritybio.com/
21. Medtronic. Permacol [Internet]. 2025 [cited 2026 Mar 2]. Available from: https://www.medtronic.com/animal-health/en-us/products/hernia-repair/permacol-surgical-implant.html#:~:text=In%20soft%20tissue%20repair%2C%20reliability,cross%2Dlinked%20for%20enhanced%20durability.

22. Kerecis. Kerecis Omega3 Wound [Internet]. 2025 [cited 2026 Mar 2]. Available from: https://www.kerecis.com/omega3-wound/
23. Zimmer Biomet. DermaSpan [Internet]. 2026 [cited 2026 Mar 2]. Available from: https://www.zimmerbiomet.com/en/products-and-solutions/specialties/biologics/dermaspan-acellular-dermal-matrix.html#:~:text=DermaSpan%20Acellular%20Dermal%20(ACD)%20Matrix,reinforcement%2C%20or%20covering%20of%20tendon.
24. Berkeley Biologics. Berkeley Biologics products [Internet]. 2026 [cited 2026 Mar 2]. Available from: https://berkeleybiologics.com/#products-warp2
25. Life NetHealth. LifeNet Health products [Internet]. 2026 [cited 2026 Mar 2]. Available from: https://www.lifenethealth.org/wound-management-surgical-reconstruction/theraskin/theraskin#:~:text=TheraSkin%20is%20an%20all%2Dhuman,epithelialization%20to%20support%20wound%20closure.
26. Marine Polymer Technologies Inc. Talymed. Public Library of Science; 2025.
27. WoundReference Inc. WoundReference [Internet]. 2026 [cited 2026 Mar 2]. Available from: https://woundreference.com/p/contents
28. Anika Therapeutics Inc. HyaloFast [Internet]. 2026 [cited 2026 Mar 2]. Available from: https://hyalofast.anikatherapeutics.com/product-information/
29. Amnio Technology LLC. PalinGen XPlusMembrane [Internet]. 2024 [cited 2026 Mar 2]. Available from: https://amniotechnology.com/index.php/palingen-xplus-membrane/
30. Acera Surgical Inc. Restrata [Internet]. 2026 [cited 2026 Mar 2]. Available from: https://acera-surgical.com/pages/products/detail/1/14#:~:text=Restrata%20is%20a%20fully%20resorbable%2C%20electrospun%20fiber,wounds%20*%20Full%2Dthickness%20wounds%20*%20Traumatic%20wounds
31. Tissue Regenix Group. DermaPure [Internet]. 2025 [cited 2026 Mar 2]. Available from: https://www.tissueregenix.com/en-uk/products/dermapure/
32. Mallinckrodt Pharmaceuticals. StrataGraft Brochure. Arch Otolaryngol Head Neck Surg [Internet]. 2021 [cited 2026 Mar 2]. Available from: https://www.multivu.com/players/English/8801751-mallinckrodt-stratagraft/docs/StratagraftPDF_1623793345102-1437039794.pdf
33. AlloSource. PureSkin-Brochure. 2023.
34. Anthem Inc., American Medical Association. Allogeneic, Xenographic, Synthetic, Bioengineered, and Composite Products for Wound Healing and Soft Tissue Grafting - Medical Policy. 2024.
35. NiMedx Group. AmnioFix presentation. 2011.
36. Musculoskeletal Transplant Foundation. SomaGen brochure. 2020.
37. Schlottmann F, Obed D, Bingöl AS, März V, Vogt PM, Krezdorn N. Treatment of Complex Wounds with NovoSorb® Biodegradable Temporising Matrix (BTM)—A Retrospective Analysis of Clinical Outcomes. J Pers Med. 2022 Dec 1;12(12). doi:10.3390/jpm12122002 PubMed PMID: 36556223.
38. Musculockeletal Transplant Foundation. AlloPatch allograft dermal matrix Pliable. Nature Publishing Group; 2020.
39. ParametricsMedical, Extremity Care. Wound Care Solutions [Internet]. 2025. Available from: www.parametricsmedical.com
40. Organogenesis. Allograft Tissue Information and NuShield® Instructions for Use Contents. 2024.
41. Musculockeletal Transplant Foundation. MTFBiologics Wound Care - Tissue & Product Catalog. 2024.
42. Musculockeletal Transplant Foundation. Salera allograft placental membrane. 2022.
43. Ben-Nakhi ME, Eltayeb HI. Three-year experience with Integra dermal regenerative template as a reconstructive tool. The Egyptian Journal of Surgery. 2020;(39):567–73. doi:10.4103/ejs.ejs_17_20

44. Integra LifeSciences Corporation. Integra Matrix Wound Dressing Instr for use. 2020.
45. Berkeley Biologics LLC. InteguPly acellular dermis. 2024.
46. Medline Industries Inc. PURACOL ® Collagen Wound Dressings. 2017.
47. Integra LifeSciences Corporation. Integra® BioFix® Amniotic Allografts. 2015.
48. Integra LifeSciences Corporation. Omnigraft dermal regeneration matrix. 2020.
49. Integra LifeSciences Corporation. Integra ® BioFix ® Flow Placental Tissue Matrix Allograft. 2015.
50. Integra LifeSciences Corporation. Integra™Flowable Wound Matrix. 2011.
51. Medline Industries Inc. Hyalomatrix ® (Non-Silicone) Hyaluronic Acid Wound Device [Internet]. 2019. Available from: www.medlinecorius.com
52. Flower Orthopedics. FlowerDerm acellular dermal allografts. 2019.
53. Medicaid, Sentara Health Plans. Skin and Tissue Substitutes, Surgical 73 Policy. 2025.
54. Aziyo Biologics. SimpliDerm hydrated acellular dermal matrix. 2019.
55. Integra LifeSciences Corporation. AmnioExcel amniotic allograft membrane. 2018.
56. Integra LifeSciences Corporation. AmnioMatrix ® Amniotic Allograft Suspension. 2019.
57. AlloSource. AlloSkin RT. 2017.
58. AlloSource. AlloSkin AC. 2025.
59. Berkeley Biologics LLC, Elutia Inc., American Association of Tissue Banks. SimpliDerm perforated - instructions for use.
60. Berkeley Biologics LLC. AltiPly Lite Lyophilized Amniotic Membrane. 2024.
61. Dermal AA. ALLOMEND® ACELLULAR DERMAL MATRIX [Internet]. 2025. Available from: www.aaos.org/news/aaosnow/jun09/research3.asp.
62. AbbVie. ALLODERM products - instructions for use. 2025.
63. Berkeley Biologics LLC. ACELLODERM. 2024.
64. Macadam SA, Lennox PA. Acellular dermal matrices: Use in reconstructive and aesthetic breast surgery. Can J Plast Surg. 2012;20(2). doi:10.1177/229255031202000201 PubMed PMID: 23730154.
65. MIHALEČKO J, BOHÁČ M, DANIŠOVIČ L, KOLLER J, VARGA I, KUNIAKOVÁ M. Acellular Dermal Matrix in Plastic and Reconstructive Surgery. Physiol Res. 2022 Dec 5;71:S51–7. doi:10.33549/physiolres.935045 PubMed PMID: 36592440.
66. Suh H, Hong JP. One Stage Allogenic Acellular Dermal Matrices (ADM) and Split-Thickness Skin Graft with Negative Pressure Wound Therapy. In: Skin Grafts. InTech; 2013. doi:10.5772/53304
67. Integra LifeSciences Corporation. AmnioExcel & AmnioMatrix. 2018.
68. AlloSource. AlloSkin split-thickness dermis. 2025.
69. Samaniego A. ALLOWRAP® SURGICAL BARRIER REMAINS IN THE BODY THROUGH THE CELL PROLIFERATION PHASE OF THE HEALING PROCESS WHILE OTHER SURGICAL BARRIERS ARE RESORBED. 2013.
70. Snyder D, Sullivan N, Margolis BD, Schoelles K. Technology Assessment Program-Technical Brief Skin Substitutes for Treating Chronic Wounds [Internet]. 2020. Available from: www.ahrq.gov

71. Boyce ST, Lalley AL. Tissue engineering of skin and regenerative medicine for wound care. Burns Trauma. 2018 Dec 1;6. doi:10.1186/s41038-017-0103-y PubMed PMID: 30009192.
